# A fungal ABC transporter FgAtm1 regulates iron homeostasis via the transcription factor cascade FgAreA-HapX

**DOI:** 10.1371/journal.ppat.1007791

**Published:** 2019-09-23

**Authors:** Zhihui Wang, Tianling Ma, Yunyan Huang, Jing Wang, Yun Chen, H. Corby Kistler, Zhonghua Ma, Yanni Yin

**Affiliations:** 1 State Key Laboratory of Rice Biology, Zhejiang University, Hangzhou, China; 2 Institute of Biotechnology, Key Laboratory of Molecular Biology of Crop Pathogens and Insects, Zhejiang University, Hangzhou, China; 3 United States Department of Agriculture, Agricultural Research Service, St. Paul, Minnesota, United States of America; Purdue University, UNITED STATES

## Abstract

Iron homeostasis is important for growth, reproduction and other metabolic processes in all eukaryotes. However, the functions of ATP-binding cassette (ABC) transporters in iron homeostasis are largely unknown. Here, we found that one ABC transporter (named FgAtm1) is involved in regulating iron homeostasis, by screening sensitivity to iron stress for 60 ABC transporter mutants of *Fusarium graminearum*, a devastating fungal pathogen of small grain cereal crops worldwide. The lack of FgAtm1 reduces the activity of cytosolic Fe-S proteins nitrite reductase and xanthine dehydrogenase, which causes high expression of FgHapX via activating transcription factor FgAreA. FgHapX represses transcription of genes for iron-consuming proteins directly but activates genes for iron acquisition proteins by suppressing another iron regulator FgSreA. In addition, the transcriptional activity of FgHapX is regulated by the monothiol glutaredoxin FgGrx4. Furthermore, the phosphorylation of FgHapX, mediated by the Ser/Thr kinase FgYak1, is required for its functions in iron homeostasis. Taken together, this study uncovers a novel regulatory mechanism of iron homeostasis mediated by an ABC transporter in an important pathogenic fungus.

## Author summary

Essential element iron plays important roles in many cellular processes in all organisms. The function of an ATP-binding cassette (ABC) transporter Atm1 in iron homeostasis has been characterized in *Saccharomyces cerevisiae*. Our study found that FgAtm1 regulates iron homeostasis via the transcription factor cascade FgAreA-HapX in *F*. *graminearum* and the function of FgHapX is dependent on its interaction with FgGrx4 and phosphorylation by the Ser/Thr kinase FgYak1. This study reveals a novel regulatory mechanism of iron homeostasis in an important plant pathogenic fungus, and advances our understanding in iron homeostasis and functions of ABC transporters in eukaryotes.

## Introduction

Iron is an essential element for growth, and can be present in various forms such as iron ions, heme and iron sulfur clusters that play critical roles in respiration, DNA synthesis and repair, ribosome biogenesis, metabolism and other cellular processes in all organisms [1–3]. In mammals, iron deficiency anemia is the most extended and common nutritional disorder in the world [4, 5]. In pathogenic fungi, the defects in iron uptake lead to decreased virulence [6, 7]. However, excess iron has the ability to generate toxic reactive oxygen species (ROS) through Fenton's reaction resulting in damage to cellular components [8]. Iron overload in liver and other organs from hepcidin regulation disorder is associated with hereditary hemochromatosis [9, 10]. Consequently, all organisms have developed tightly homeostatic regulatory mechanisms to balance uptake, consumption and storage of iron.

ATP-binding cassette (ABC) transporters that contain transmembrane domains (TMDs) and structurally conserved nucleotide-binding domains (NBDs) actively transport a wide variety of compounds across biological membranes [11]. ABC transporters play important roles in transporting compounds and regulating various physiological processes, including fatty acid metabolism, ribosome biogenesis, and mRNA translation [12, 13]. Recently, ABC transporters have been implicated in endocytosis and hyphal formation in *Candida albicans* [14], autophagy in human [15, 16], lifespan regulation in *Drosophila* [17], and the establishment of terrestrial lifestyle in plants [18]. However, our understanding of ABC transporters involved in iron homeostasis is limited. In *S*. *cerevisiae*, two ABC transporters Atm1 and Mdl1 have been found to be associated with iron homeostasis. Atm1 regulates the assembly of cytoplasmic and nucleic iron-sulfur (Fe-S) proteins might via transporting glutathione (GSH)-linked [2Fe-2S] clusters ((GS)_4_-[2Fe-2S]) from mitochondria to cytosol [19–20]. Mdl1 exports the proteolytic products generated by the m-AAA protease, and the over-expression of Mdl1 partially restores the defects in Atm1 mutant [21, 22]. In addition, an ABC transporter in *Mycosphaerella graminicola* (MgAtr7) harboring a dityrosine/pyoverdine biosynthetic domain is required for siderophore production and subsequently modulates iron homeostasis [23].

To date, Atm1 homologs have been found to control assembly of cytoplasmic and nuclear Fe-S proteins in *S*. *cerevisiae*, *Arabidopsis thaliana* and *Homo sapiens* [24–26]. But the regulatory mechanism of iron homeostasis modulated by Atm1 is only characterized in *S*. *cerevisiae* [27–30]. In the budding yeast, the Fe-S proteins monothiol glutaredoxins Grx3/4 sense GSH-linked [2Fe-2S] clusters exported by Atm1 from mitochondria to cytoplasm [31]. Depletion of *ATM1* impairs the loading of GSH-linked [2Fe-2S] clusters onto monothiol glutaredoxins Grx3/4, thus hindering the formation of the complex containing Grx3/4 and the cytosolic proteins Fra1/2. This subsequently enhances retention of the transcription factor Aft1/2 at the promoter of iron acquisition genes, therefore leading to constitutive gene activation [27–31]. Except for the budding yeast, the functions and regulatory mechanisms of Atm1 orthologs in iron homeostasis have not been documented in other organisms.

*F*. *graminearum* is an economically important plant pathogen that causes cereal scab disease worldwide [32]. In addition to yield reduction, mycotoxins such as deoxynivalenol (DON) and zearalenone (ZEA) produced by the causal agent constitute a serious threat to food security and human health [33]. *F*. *graminearum* contains many more ABC transporters than most other representative fungi from major evolutionary lineages within the fungal kingdom [34, 35]. After the screening of 60 ABC knockout mutants for sensitivity to iron stress, we found that only the FgAtm1 (Atm1 ortholog) mutant was highly sensitive, whereas the mutants of Mdl1 (FGSG_01885) and MgAtr7 (FGSG_03735) orthologs were not involved in iron regulation in *F*. *graminearum*. We therefore focused on exploring the functions of FgAtm1 in regulating iron homeostasis in *F*. *graminearum*.

In this study, we revealed that the deletion of *FgATM1* impedes the activity of cytosolic Fe-S proteins nitrite reductase and xanthine dehydrogenase, which in turn induces transcription factor FgAreA, and subsequently activates the transcription factor FgHapX. The phosphorylation of FgHapX is mediated by the Ser/Thr kinase FgYak1 and is further required for the transcriptional regulation of iron-related genes. It is worth to note that this interaction between FgHapX and the monothiol glutaredoxin FgGrx4 is also required for the transcriptional activity of FgHapX, which is dramatically different from what is known in the budding yeast. Overall, results from this study reveal a regulatory mechanism of iron homeostasis mediated by FgAtm1 and the transcription factor cascade FgAreA-HapX in *F*. *graminearum*, which will help us improve the understanding of iron-homeostatic regulation in eukaryotes.

## Results

### Deletion of *FgATM1* led to the accumulation of iron in *F*. *graminearum*

**Fig 1:**
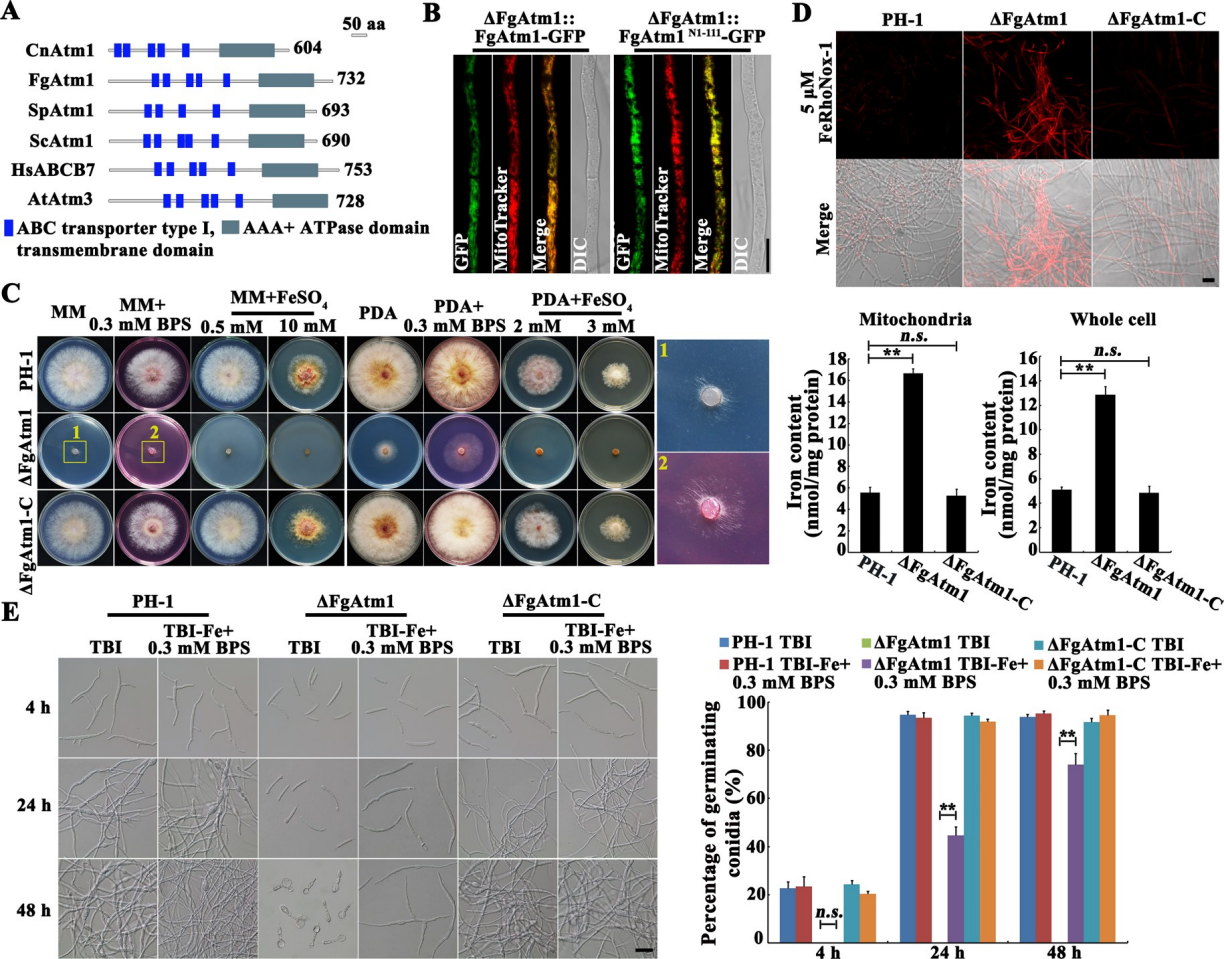
Deletion of FgAtm1 led to iron accumulation. (A) Domain analyses of Atm1 orthologs from *F*. *graminearum* and other eukaryotic species. The domain analysis was performed with InterPro Scan program of the Interpro protein database (http://www.ebi.ac.uk/interpro). (B) Colocalization of FgAtm1 or FgAtm1^N1-111^ (truncated FgAtm1 containing only the N-terminal 111 amino acids) with mitochondrial dye MitoTracker. The plasmid FgAtm1- or FgAtm1^N1-111^-GFP was ectopically transformed into ΔFgAtm1, and the resulting strain was then examined with a fluorescent microscope after MitoTracker staining. Bar = 10 μm. (C) Sensitivity of the wild-type strain PH-1, ΔFgAtm1 and the complemented transformant ΔFgAtm1-C to iron chelating agent BPS and FeSO_4_. A 5-mm mycelial plug of each strain was inoculated on MM or PDA without or with 0.3 mM BPS or FeSO_4_ at the indicated concentration, and then incubated at 25°C for 3 days. (D) Iron content in mitochondria and whole cell of the wild type, ΔFgAtm1 and ΔFgAtm1-C was determined by a laser scanning microscope with 5 μΜ fluorescent iron-binding dye FeRhoNox-1 (upper panel) or colorimetric ferrozine-based assay (lower panel) after culture in CM at 25°C for 36 hours. Bar = 20 μm. Means and standard errors were calculated from three repeats. Significance was measured using unpaired *t*-test (*n*.*s*. not significant, **p < 0.01). (E) Conidial germination of the wild type, ΔFgAtm1 and ΔFgAtm1-C in trichothecene biosynthesis induction medium (TBI) or iron-depleted TBI. Bar = 20 μm. Percentage of germinating conidia of each strain was calculated after 4-, 24- and 48-hour-incubation. Means and standard errors were calculated from three repeats. Significance was measured using unpaired *t*-test (*n*.*s*. not significant, **p < 0.01).

*F*. *graminearum* contains 62 putative ABC transporters. In order to explore functions of ABC transporters, we deleted each of them using a homology recombination strategy. Among 62 ABC transporter genes, 60 were deleted successfully, and two genes FGSG_07101 and FGSG_04181 are essential for *F*. *graminearum* growth [35]. To explore functions of ABC transporters in iron homeostasis, we screened these 60 deletion mutants for the sensitivity to iron stress and found that the mutant of FGSG_10911 was supersensitive to iron stress (S1 Fig), indicating that this ABC transporter may play important roles in iron homeostasis regulation. The BLAST analysis showed that FGSG_10911 is homologous to *S*. *cerevisiae* Atm1 (Fig 1A), and thus we named the gene FgAtm1. We complemented ΔFgAtm1 with FgAtm1-GFP and N-terminal mitochondrion-targeting sequence of FgAtm1 (FgAtm1^N1-111^)-GFP, respectively. Subcellular localization observation revealed that FgAtm1-GFP co-localized with the mitochondrial dye MitoTracker, and the N-terminal mitochondrion-targeting sequence (http://www.cbs.dtu.dk/services/TargetP/) is thought to be responsible for its mitochondrial localization (Fig 1B and S2 Fig).

Phenotypic characterization showed that ΔFgAtm1 displayed hypersensitivity to 0.5 mM Fe^2+^ and 2 mM Fe^2+^ supplemented into minimal medium (MM) and potato dextrose agar (PDA), respectively (Fig 1C). In contrast, after treatment with iron-specific chelating agent bathophenanthroline disulfonate (BPS) at 0.3 mM, ΔFgAtm1 grew better than untreated cultures on MM and PDA (Fig 1C). Determination of intracellular iron by using fluorescent iron-binding dye FeRhoNox-1 and a colorimetric ferrozine-based assay revealed a high level of iron accumulation in both mitochondria and the whole cell of ΔFgAtm1 (Fig 1D). The iron content in mitochondria was clearly higher than that in whole cell (Fig 1D). In addition, the effect of iron stress on conidial germination was also determined. As shown in Fig 1E, 98% of the wild type conidia germinated after incubation at 28°C for 24 h in the trichothecene biosynthesis induction (TBI) medium that contains a trace amount of Fe^2+^ [36], while, ΔFgAtm1 conidia did not germinate even after 48 h under the same conditions. When 0.3 mM BPS was added into TBI to chelate Fe^2+^, 44% and 72% of ΔFgAtm1 conidia germinated after incubation for 24 and 48 h, respectively. Conidial germination in the wild type was not affected by BPS treatment (Fig 1E). To further confirm that the supersensitivity of ΔFgAtm1 to iron stress is due to the deletion of the *FgATM1* gene, the mutant was complemented with a full-length wild-type *FgATM1* gene amplified with the primers listed in S1 Table. The complemented strain ΔFgAtm1-C contained a single copy of *FgATM1*, which was inserted into the genome of ΔFgAtm1 (S3 Fig). The defects of mycelial growth, conidial germination and accumulation of iron in ΔFgAtm1 were restored to the wild-type phenotypes in ΔFgAtm1-C (Fig 1C–1E). These results strongly indicate that the lack of FgAtm1 leads to accumulation of intracellular iron in *F*. *graminearum*.

### FgAtm1 regulates activity of cytosolic Fe-S proteins

**Fig 2:**
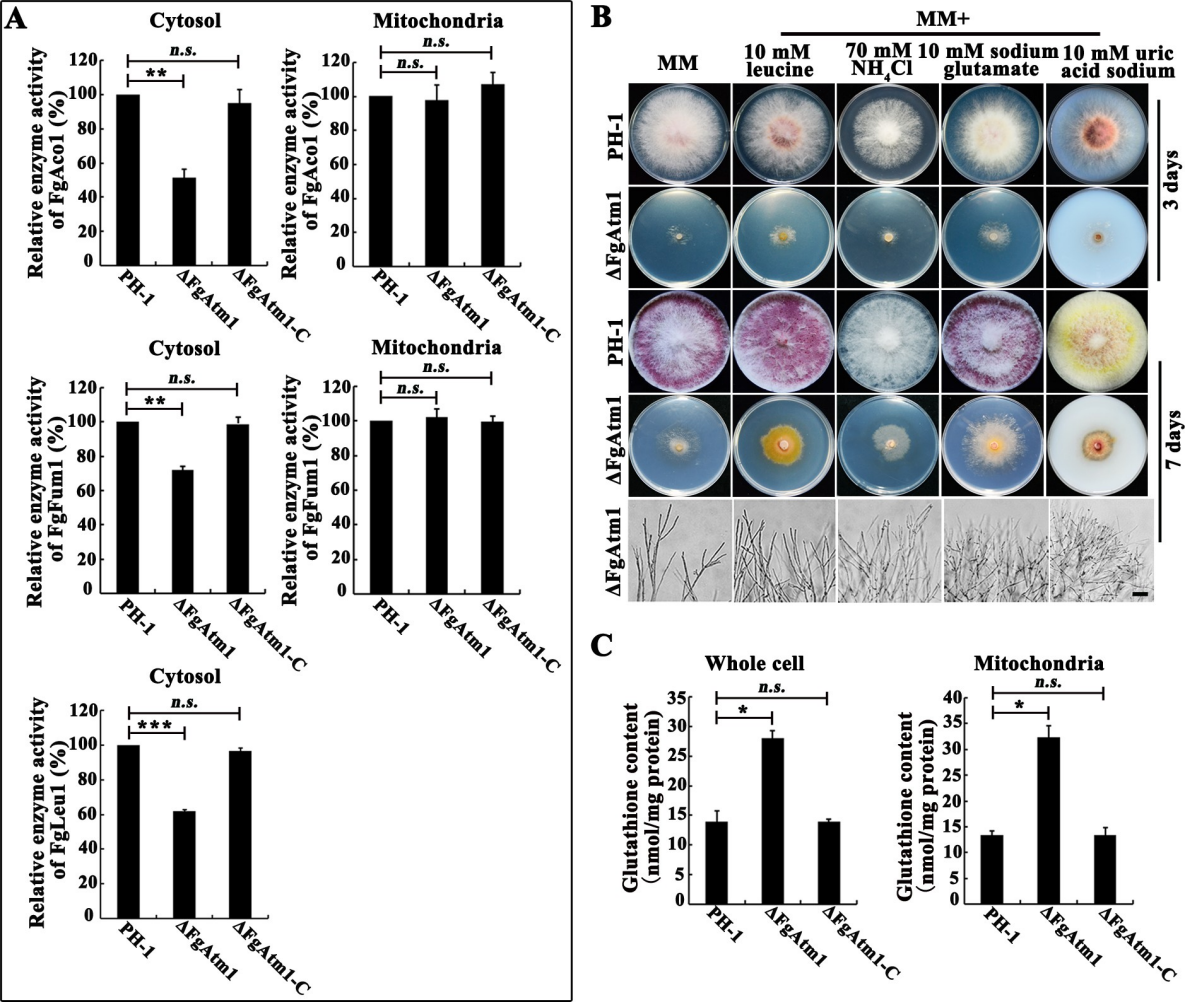
FgAtm1 regulates the activity of cytosolic Fe-S proteins. (A) Determination of FgLeu1, FgAco1 and FgFum1 activities in mitochondria and cytosol of the wild type, ΔFgAtm1 and ΔFgAtm1-C. The FgLeu1, FgAco1 and FgFum1 activity of the wild type were set as 1. Each strain was cultured in CM at 25°C for 36 hours before activity determination. Means and standard errors were calculated from three repeats. Significance was measured using unpaired *t*-test (*n*.*s*. not significant, **p < 0.01, ***p < 0.001). (B) The growth and microscopic observation of colony edge of the wild type and ΔFgAtm1 on MM with or without 10 mM leucine, 70 mM NH_4_Cl, 10 mM sodium glutamate or 10 mM uric acid sodium at 25°C for 3 day or 7 days. Bar = 100 μm. (C) The content of total glutathione in mitochondria and whole cell of the wild type, ΔFgAtm1 and ΔFgAtm1-C. Means and standard errors were calculated from three repeats. Significance was measured using unpaired *t*-test (*n*.*s*. not significant, *p < 0.05).

To determine whether FgAtm1 regulates the assembly of cytosolic Fe-S proteins, we studied the activity of Fe-S proteins isopropyl malate isomerase (FgLeu1, FGSG_ 09589), aconitase (FgAco1, FGSG_07953), fumarase (FgFum1, FGSG_08712). *S*. *cerevisiae* Leu1 is a cytosolic Fe-S protein and catalyzes the second step in leucine biosynthesis [26]. Cytosolic and mitochondrial Fe-S proteins Aco1 and Fum1 both participate in glyoxylate shunt in cytosol and TCA cycle in mitochondria in the budding yeast, respectively [37, 38]. Enzyme activity assays showed that the activities of FgLeu1, FgAco1 and FgFum1 in the cytosol of ΔFgAtm1 were attenuated by 37, 44 and 28% respectively, when compared to those in the wild type. In contrast, the activities of FgAco1 and FgFum1 in ΔFgAtm1 mitochondria were not significantly changed (Fig 2A). Further, feeding ΔFgAtm1 with leucine (the final catalytic product of FgLeu1) or the final catalytic product of other cytosolic Fe-S proteins nitrite reductase, glutamate dehydrogenase or xanthine dehydrogenase [39, 40] accelerated the growth of ΔFgAtm1 on MM (Fig 2B). In *S*. *cerevisiae*, GSH-linked [2Fe-2S] clusters were reported to be the substrate of Atm1, and deletion of *ATM1* caused increased GSH content in the whole cell [19, 20, 41]. We therefore tested the content of GSH, and found that it was increased by 59% and 50% in mitochondria and the whole cell of ΔFgAtm1, respectively (Fig 2C). Similar to these reported in the budding yeast [19–20], the results of this study indicate that FgAtm1 also modulates the assembly of cytosolic Fe-S proteins likely via transporting GSH-linked [2Fe-2S] clusters from mitochondria into *F*. *graminearum* cytoplasm.

### The deletion of FgAtm1 activates the transcription factor cascade FgAreA-HapX

**Fig 3:**
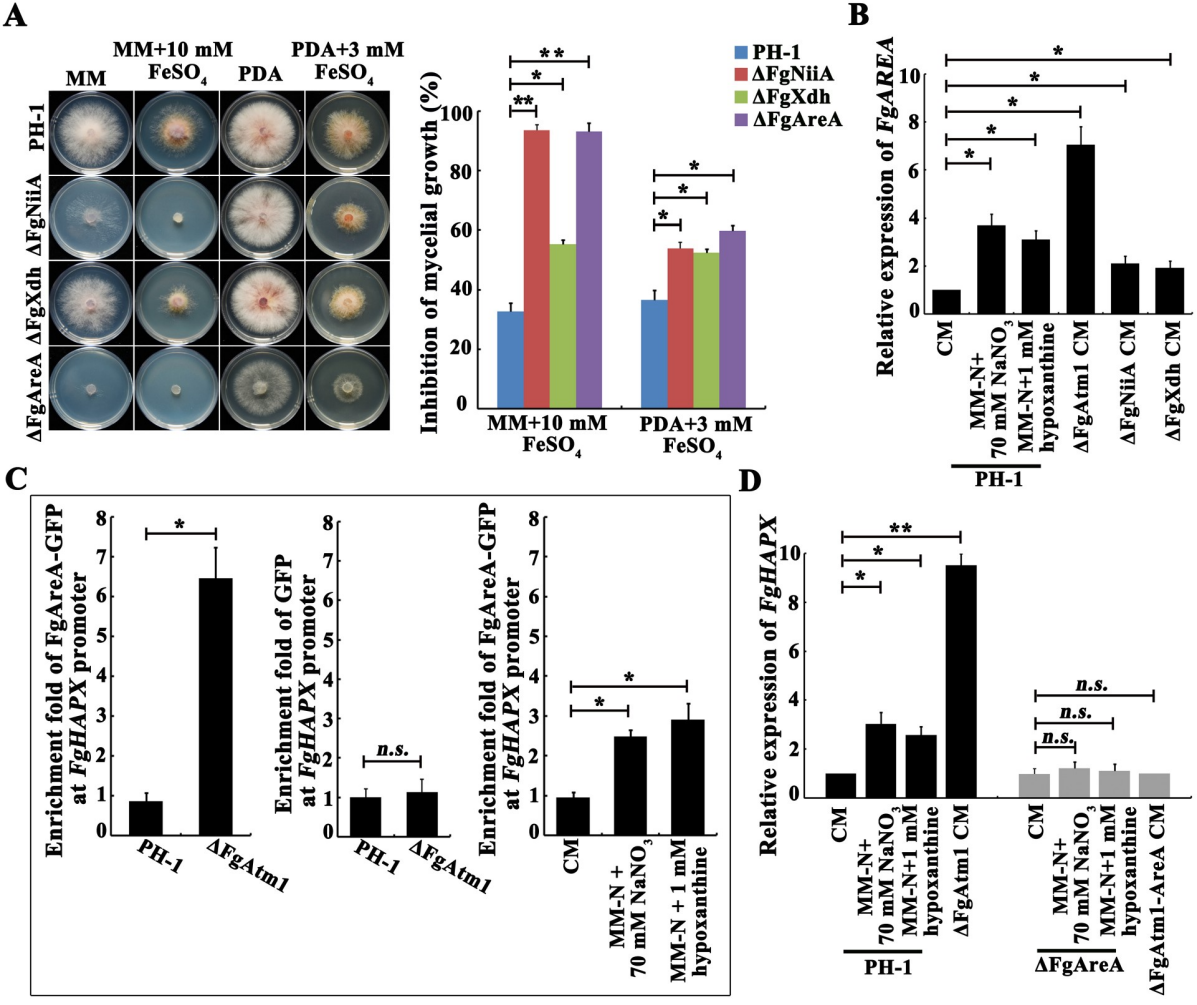
The reduced activity of cytoplasmic Fe-S proteins activates transcription of the FgAreA-HapX cascade. (A) Sensitivity of the wild type, ΔFgNiiA, ΔFgXdh and ΔFgAreA to FeSO_4_. A 5-mm mycelial plug of each strain was inoculated on MM or PDA without or with FeSO_4_ at the indicated concentration, and then incubated at 25°C for 3 days. Mycelial growth inhibition of each treatment was calculated after a 3-day-incubation. Means and standard errors were calculated from three repeats. Significance was measured using an unpaired *t*-test (*p < 0.05, **p < 0.01). (B) Relative transcription levels of *FgAREA* treated by NaNO_3_ or hypoxanthine, or deletion of *FgATM1*, *FgNIIA* or *FgXDH*. PH-1 was treated with MM-N+70 mM NaNO_3_ or MM-N+1 mM hypoxanthine for 4 hours after culture in CM at 25°C for 1 day. The expression level in the wild type without treatment was set as 1. Means and standard errors were calculated from three repeats. Significance was measured using unpaired *t*-test (*p < 0.05). (C) The enrichment of FgAreA-GFP at the promoter of *FgHAPX* was induced by the treatment with NaNO_3_ or hypoxanthine, or deletion of *FgATM1*. Each strain was treated with MM-N+70 mM NaNO_3_ or MM-N+1 mM hypoxanthine for 4 hours after culture in CM at 25°C for 1 day. ChIP- and input-DNA samples were quantified by quantitative PCR assay. A control reaction was processed in parallel with rabbit IgG and PH-1 transformed only with GFP used as a negative control for detecting GFP enrichment at the *FgHAPX* promoter. Means and standard errors were calculated from three repeats. Significance was measured using unpaired *t*-test (*n*.*s*. not significant, *p < 0.05). (D) Relative transcription levels of *FgHAPX* in the wild-type PH-1 and ΔFgAreA under a non-preferred nitrogen source or in the background of *FgATM1* deletion. Each strain was treated with MM-N+70 mM NaNO_3_ or MM-N+1 mM hypoxanthine for 4 hours after culture in CM at 25°C for 1 day. The expression level in the wild type without treatment was set as 1. Means and standard errors were calculated from three repeats. Significance was measured using unpaired *t*-test (*n*.*s*. not significant, *p < 0.05, **p < 0.01).

To further explore the regulatory mechanism of FgAtm1 in iron homeostasis, we determined iron stress sensitivity for nine cytoplasmic Fe-S protein mutants constructed in our laboratory and found that nitrite reductase (FgNiiA, FGSG_08402) and xanthine dehydrogenase (FgXdh, FGSG_01561) mutants showed increased sensitivity to Fe^2+^ (Fig 3A and S5 Fig). Similar to what were reported in the yeasts [37, 38, 42, 43], the remaining proteins that we tested were not involved in iron stress responses. Previous studies have shown that nitrite reductase and xanthine dehydrogenase are key enzymes for non-preferred nitrogen source utilization, and are regulated by nitrogen metabolism regulator AreA in *Aspergillus nidulans*, *F*. *oxysporum* and *F*. *graminearum* [40, 44–47]. Nitrite reductase is responsible for nitrate utilization [40] and xanthine dehydrogenase is required for oxidizing hypoxanthine to xanthine [48]. Quantitative reverse transcription PCR (qRT-PCR) assays showed that transcription of *FgAREA* was induced by the deletion of *FgATM1*, *FgNIIA* (encoding nitrite reductase) or *FgXDH* (encoding xanthine dehydrogenase), as well as by the non-preferred nitrogen sources, NaNO_3_ or hypoxanthine (Fig 3B). Surprisingly, we found that ΔFgAreA also exhibited elevated sensitivity to Fe^2+^ (Fig 3A). Therefore, we hypothesized that the deletion of FgAtm1 leads to reduced activities of FgNiiA and FgXdh, which induces overexpression of FgAreA.

To explore the role of FgAreA in regulating iron homeostasis, we first performed serial analysis of gene expression (SAGE) assay for the mutant ΔFgAtm1, and found that 56 iron-related genes were differentially expressed (>2-fold) in ΔFgAtm1 (S2 Table). Further, qRT-PCR assay confirmed that the transcription level of the transcription factor *FgHAPX* was dramatically increased in ΔFgAtm1 as compared to that of the wild type (S6 Fig). To understand the mechanism by which FgAreA regulates *FgHAPX* expression, we studied the binding ability of FgAreA to the promoter of *FgHAPX* in the wild type bearing FgAreA-GFP (PH-1::FgAreA-GFP) and in ΔFgAtm1 bearing FgAreA-GFP (ΔFgAtm1::FgAreA-GFP) using chromatin immunoprecipitation and quantitative PCR (ChIP-qPCR) assay. A strain transformed with GFP alone was used as a negative control. ChIP-qPCR analyses showed that enrichment of FgAreA at the *FgHAPX* promoter was induced by the deletion of *FgATM1* as well as the treatment by NaNO_3_ or hypoxanthine (Fig 3C). GFP enrichment at the *FgHAPX* promoter was undetectable in the negative control strain (Fig 3C). Additionally, qRT-PCR assays revealed that *FgHAPX* transcription was also induced with NaNO_3_ or hypoxanthine treatment. Moreover the induced expression of *FgHAPX* upon non-preferred nitrogen source treatment was dependent on FgAreA (Fig 3D). These results indicated that FgAreA binds to the promoter of *FgHAPX* and regulates its transcription.

### FgHapX suppresses transcription of iron-consuming genes directly and also activates transcription of iron acquisition genes by repressing another transcription factor FgSreA

**Fig 4:**
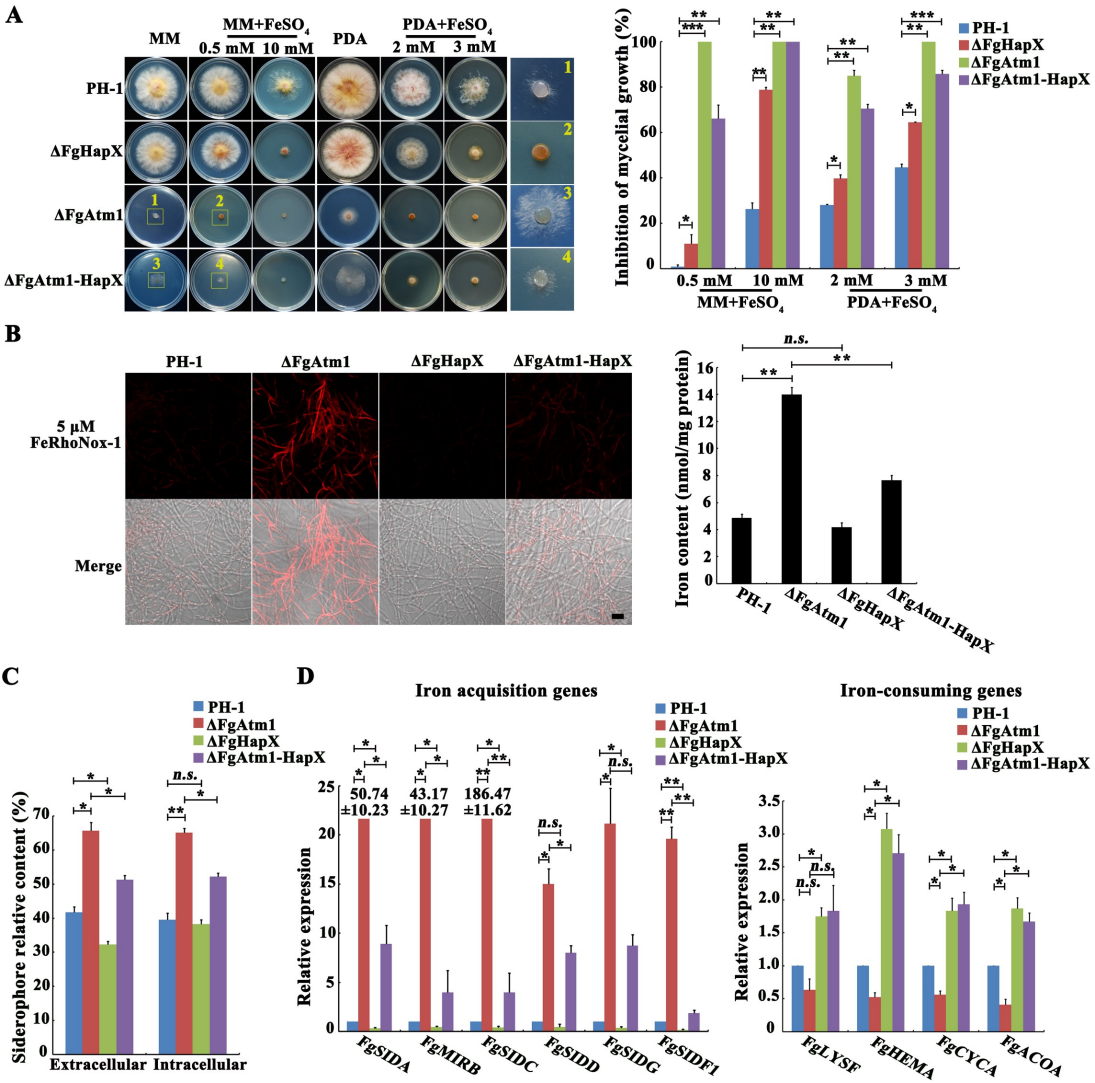
Over-expression of *FgHAPX* leads to iron accumulation in ΔFgAtm1. (A) Sensitivity of the wild type, ΔFgAtm1, ΔFgHapX and ΔFgAtm1-HapX to FeSO_4_. A 5-mm mycelial plug of each strain was inoculated on MM or PDA without or with FeSO_4_ at the indicated concentration, and then incubated at 25°C for 3 days. Mycelial growth inhibition of each treatment was calculated after 3-day-incubation. Means and standard errors were calculated from three repeats. Significance was measured using unpaired *t*-test (*p < 0.05, **p < 0.01, ***p < 0.001). (B) Total iron content of the wild type, ΔFgAtm1, ΔFgHapX and ΔFgAtm1-HapX was determined by a laser scanning microscope with 5 μΜ fluorescent iron-binding dye FeRhoNox-1 (left panel) or colorimetric ferrozine-based assay (right panel) after culture in CM at 25°C for 36 hours. Bar = 20 μm. Means and standard errors were calculated from three repeats. Significance was measured using unpaired *t*-test (*p < 0.05, **p < 0.01). (C) The content of extra- and intracellular siderophores of the wild type, ΔFgAtm1, ΔFgHapX and ΔFgAtm1-HapX determined by the chrome azurol S (CAS) assay. Each strain was cultured in MM lack of Fe^2+^ for 8 hours after growth in CM for 36 hours. Means and standard errors were calculated from three repeats. Significance was measured using unpaired *t*-test (*n*.*s*. not significant, *p < 0.05, **p < 0.01). (D) Relative transcription levels of the genes involved in iron homeostasis in the wild type, ΔFgAtm1, ΔFgHapX and ΔFgAtm1-HapX cultured in CM for 1 day. The expression level of each gene in each mutant is the relative amount of mRNA compared to the level in the wild type. Mean and standard error of each gene were calculated with results from three repeats. Significance was measured using unpaired *t*-test (*n*.*s*. not significant, *p < 0.05, **p < 0.01).

To explore the function of FgHapX in iron homeostasis, we first constructed a *FgHAPX* deletion mutant ΔFgHapX, and tested the sensitivity of ΔFgHapX to iron stress. As shown in Fig 4A and 4B, ΔFgHapX became more sensitive to iron stress in comparison with the wild type, although ΔFgHapX did not show an obvious change in total iron content. Furthermore, we determined the content of extra- and intracellular siderophores secreted by ΔFgHapX with a chrome azurol S (CAS) assay, and found that the lack of *FgHAPX* caused reduced extracellular siderophore but not intracellular siderophore (Fig 4C). Similarly, qRT-PCR assays revealed that iron acquisition genes were down-regulated and iron-consuming genes were up-regulated in ΔFgHapX (Fig 4D). We knocked out *FgHAPX* in ΔFgAtm1, and checked whether the defects of ΔFgAtm1 were partially recovered by deletion of *FgHAPX*. As we expected, the double mutant ΔFgAtm1-HapX grew better and displayed decreased sensitivity to iron stress than ΔFgAtm1 (Fig 4A). Determination of iron and siderophore revealed that lack of *FgHAPX* in ΔFgAtm1 led to a reduced iron concentration, and decreased extra- and intracellular siderophores in comparison with those in ΔFgAtm1 (Fig 4B and 4C). Expression levels of iron acquisition genes in ΔFgAtm1-HapX were reduced and the transcription of iron-consuming genes were elevated compared to those in ΔFgAtm1 (Fig 4D).

**Fig 5:**
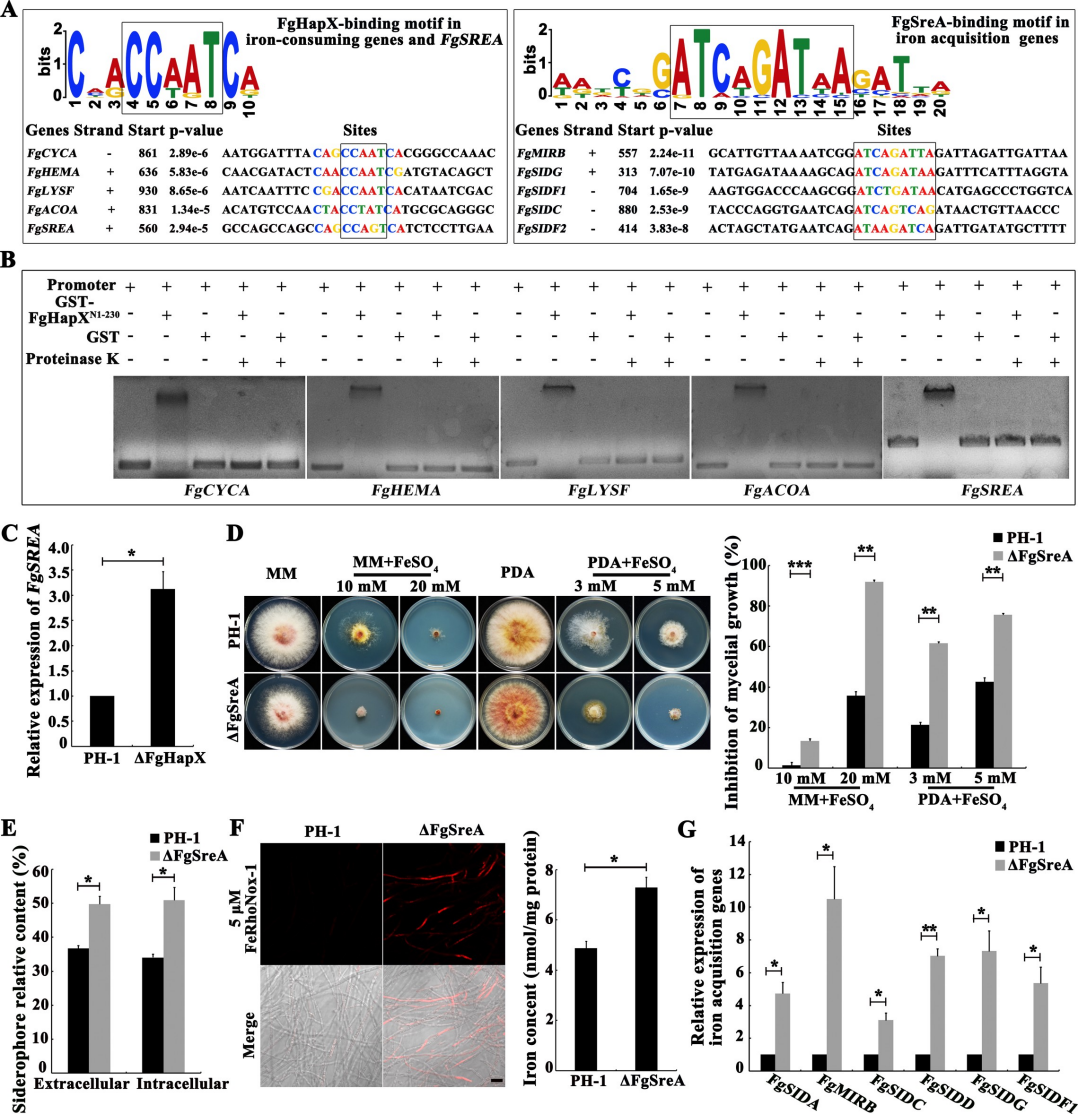
FgHapX activates iron acquisition genes via suppressing the transcriptional repressor FgSreA. (A) Identification of FgHapX and FgSreA binding motifs (indicated by black squares) by the multiple EM for motif elicitation (MEME) program. Four iron-consuming genes and *FgSREA* were used for the FgHapX binding motif analysis, and five iron acquisition genes were used for the FgSreA binding motif analysis. (B) Verification of the binding of FgHapX with the promoters of four iron-consuming genes and *FgSREA* by electrophoretic mobility shift assay (EMSA). The promoter of each gene was incubated with purified GST-FgHapX^N1–230^ or GST with or without proteinase K for 20 min at 25°C. (C) Relative transcription levels of *FgSREA* in the wild type and ΔFgHapX cultured in CM for 1 day. The relative expression level of *FgSREA* in each mutant is the relative amount of mRNA in the wild type. Means and standards error of each gene were calculated from three repeats. Significance was measured using an unpaired *t*-test (*p < 0.05, **p < 0.01). (D) Sensitivity of the wild type and ΔFgSreA to FeSO_4_. A 5-mm mycelial plug of each strain was inoculated on MM or PDA without or with FeSO_4_ at the indicated concentration, and then incubated at 25°C for 3 days. Mycelial growth inhibition of each treatment was calculated after a 3-day-incubation. Means and standard errors were calculated from three repeats. Significance was measured using an unpaired *t*-test (**p < 0.01, ***p < 0.001). (E) The content of extra- and intracellular siderophores in ΔFgSreA was increased as compared to that in the wild type in the chrome azurol S (CAS) assay. Each strain was cultured in MM lacking Fe^2+^ for 8 hours after growth in CM for 36 hours. Means and standard errors were calculated from three repeats. Significance was measured using an unpaired *t*-test (*p < 0.05). (F) Total iron content of the wild type or ΔFgSreA was determined by a laser scanning microscope with 5 μΜ fluorescent iron-binding dye FeRhoNox-1 (left panel) or colorimetric ferrozine-based assay (right panel) after each strain was cultured in CM at 25°C for 36 hours. Bar = 20 μm. Means and standard errors were calculated from three repeats. Significance was measured using an unpaired *t*-test (*p < 0.05). (G) Relative transcription levels of six iron acquisition genes in ΔFgSreA cultured in CM for 1 day. The relative expression level of each gene in ΔFgSreA is the amount of mRNA relative to that of the wild type. The expression level of each iron acquisition gene in PH-1 was set to 1. Means and standard errors were calculated from three repeats. Significance was measured using an unpaired *t*-test (*p < 0.05, **p < 0.01).

HapX homologs in *A*. *nidulans* and *A*. *fumigatus* have been found to repress the transcription of iron-consuming genes by binding to CCAAT motif [49, 50]. The multiple EM for motif elicitation (MEME) analyses showed that the genes *FgCYCA*, *FgHEMA*, *FgLYSF* and *FgACOA* involved in the iron-consuming have the CCAAT motif (Fig 5A). Electrophoretic mobility shift assay (EMSA) further confirmed that FgHapX bound the promoters of iron-consuming genes (Fig 5B). Iron acquisition genes contained the GATA, but not CCAAT, motif in their promoters (Fig 5A), indicating that other regulator(s) modulates the transcription of iron uptake genes directly in *F*. *graminearum*. In *A*. *fumigatus*, HapX activates the expression of siderophore-mediated iron uptake genes via transcriptional repression of SreA that suppresses the transcription of iron acquisition genes via binding to the GATA motif in their promoter [50, 51]. The MEME analysis and EMSA assay showed that FgHapX could bind the *FgSREA* (SreA homolog) promoter (Fig 5A and 5B). Moreover, the qRT-PCR assay revealed that deletion of *FgHAPX* led to elevated transcription of *FgSREA* (Fig 5C). We further obtained a *FgSREA* deletion mutant ΔFgSreA, and found that ΔFgSreA displayed increased sensitivity to iron stress (Fig 5D). The deletion of *FgSREA* caused iron accumulation, and increased extra- and intracellular siderophores (Fig 5E and 5F), and qRT-PCR assays showed that deletion of *FgSREA* caused elevated expression of iron acquisition genes (Fig 5G). These results indicated that FgHapX represses the transcription of FgSreA, and subsequently activates transcription of iron acquisition genes.

### FgHapX interacts with FgGrx4 to regulate iron homeostasis

**Fig 6:**
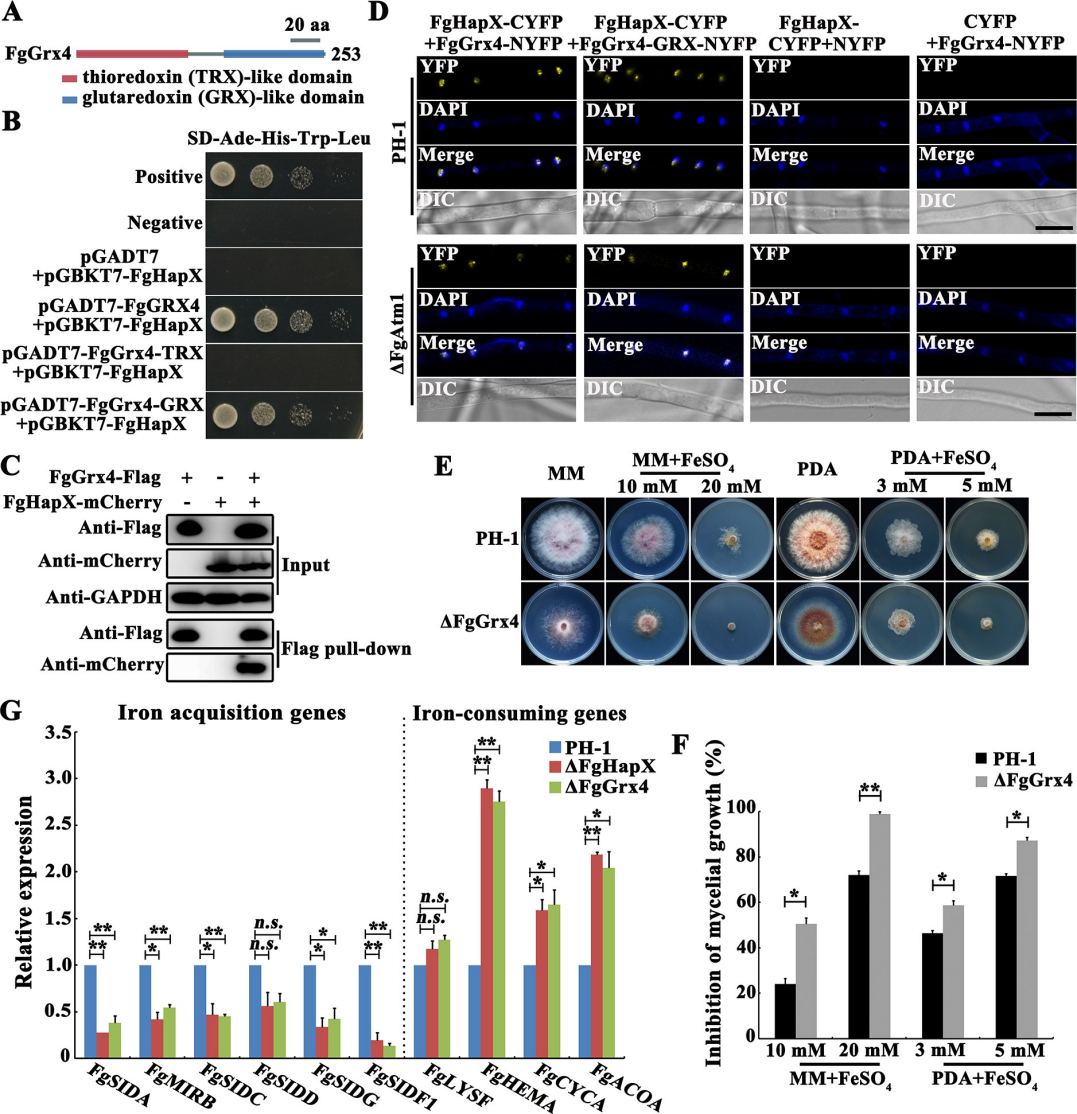
FgHapX interacts with FgGrx4 to regulate iron homeostasis in *F*. *graminearum*. (A) Domain architecture of the *F*. *graminearum* FgGrx4 protein analyzed with InterPro Scan program of the Interpro protein database (http://www.ebi.ac.uk/interpro). FgGrx4 contains a thioredoxin (TRX)-like domain and a glutaredoxin (GRX)-like domain. (B) The GRX domain of FgGrx4 is required for its interaction with FgHapX in yeast two-hybrid hybridization (Y2H) assays. Serial dilutions of yeast cells (cells/ml) transferred with the bait and prey constructs were assayed for growth on SD-Leu-Trp-His plates. A pair of plasmids pGBKT7-53 and pGADT7 was used as a positive control. A pair of plasmids pGBKT7-Lam and pGADT7 was used as a negative control. (C) FgHapX interacts with FgGrx4 in the co-immunoprecipitation (Co-IP) assay. Western blots of total proteins (input) from transformants bearing the FgGrx4-Flag and/or FgHapX-mCherry constructs (upper panel), and the proteins eluted from anti-Flag agarose beads (lower panel) were detected with the anti-mCherry or anti-Flag antibody. Detection with the anti-GAPDH antibody was conducted for the protein loading reference. (D) The interaction of FgHapX and FgGrx4 in the nucleus is dependent on the GRX domain of FgGrx4, but independent on FgAtm1 by bimolecular fluorescence complementation (BiFC) assay. A pair of constructs FgHapX-CYFP+NYFP, and another pair of constructs FgGrx4-NYFP+CYFP were used as negative controls. YFP signals were observed using confocal microscopy. Bar = 10 μm. (E) Sensitivity of the wild type and ΔFgGrx4 to FeSO_4_. A 5-mm mycelial plug of each strain was inoculated on MM or PDA without or with FeSO_4_ at the indicated concentration, and then incubated at 25°C for 3 days. (F) Mycelial growth inhibition of each treatment was calculated after 3-day-incubation (E). Means and standard errors were calculated from three repeats. Significance was measured using an unpaired *t*-test (*p < 0.05, **p < 0.01). (G) Relative transcription levels of iron-related genes in the wild type, ΔFgHapX and ΔFgGrx4 after growth in CM for 1 day. The relative expression level of each gene in each mutant is the amount of mRNA relative to the wild type. Means and standards error of each gene were calculated from three repeats. Significance was measured using an unpaired *t*-test (*n*.*s*. not significant, *p < 0.05, **p < 0.01).

To explore the regulatory mechanism of FgHapX in iron homeostasis, we performed a yeast two-hybrid (Y2H) screen of *F*. *graminearum* cDNA library, and found 50 potential FgHapX-interacting proteins (S3 Table), including the monothiol glutaredoxin FgGrx4 that is homologous to *S*. *cerevisiae* Grx3/4. Furthermore, Y2H, Co-IP and BiFC assays revealed that FgGrx4 interacted with FgHapX in the nucleus and the interaction was independent of FgAtm1 (Fig 6A–6D). Moreover, Y2H and BiFC assays showed that FgGrx4 interacted with FgHapX through its GRX domain but not its TRX domain (Fig 6B and 6D). In *S*. *cerevisiae*, lack of Grx3/4 leads to constitutive expression of iron acquisition genes, which contributes to iron accumulation in cells [52, 53]. We therefore generated a *FgGRX4* deletion mutant, ΔFgGrx4, and found that ΔFgGrx4 displayed increased sensitivity to iron stress although it did not exhibit an obvious alteration in the total iron content (Fig 6E and S7A Fig). To explore the effect of FgGrx4 on FgHapX functions, we determined the quantity and localization of FgHapX in ΔFgGrx4, and found that *FgGRX4* deletion did not cause an obvious change in the localization and quantity of FgHapX (S7B and S7C Fig). However, similar to the FgHapX deletion, the deletion of *FgGRX4* led to significantly decreased expression of iron acquisition genes, and increased transcription of iron-consuming genes (Fig 6G). These results indicated that FgGrx4 is required for the transcriptional activity of FgHapX in *F*. *graminearum*.

### FgHapX is phosphorylated by the Ser/Thr kinase FgYak1

**Fig 7:**
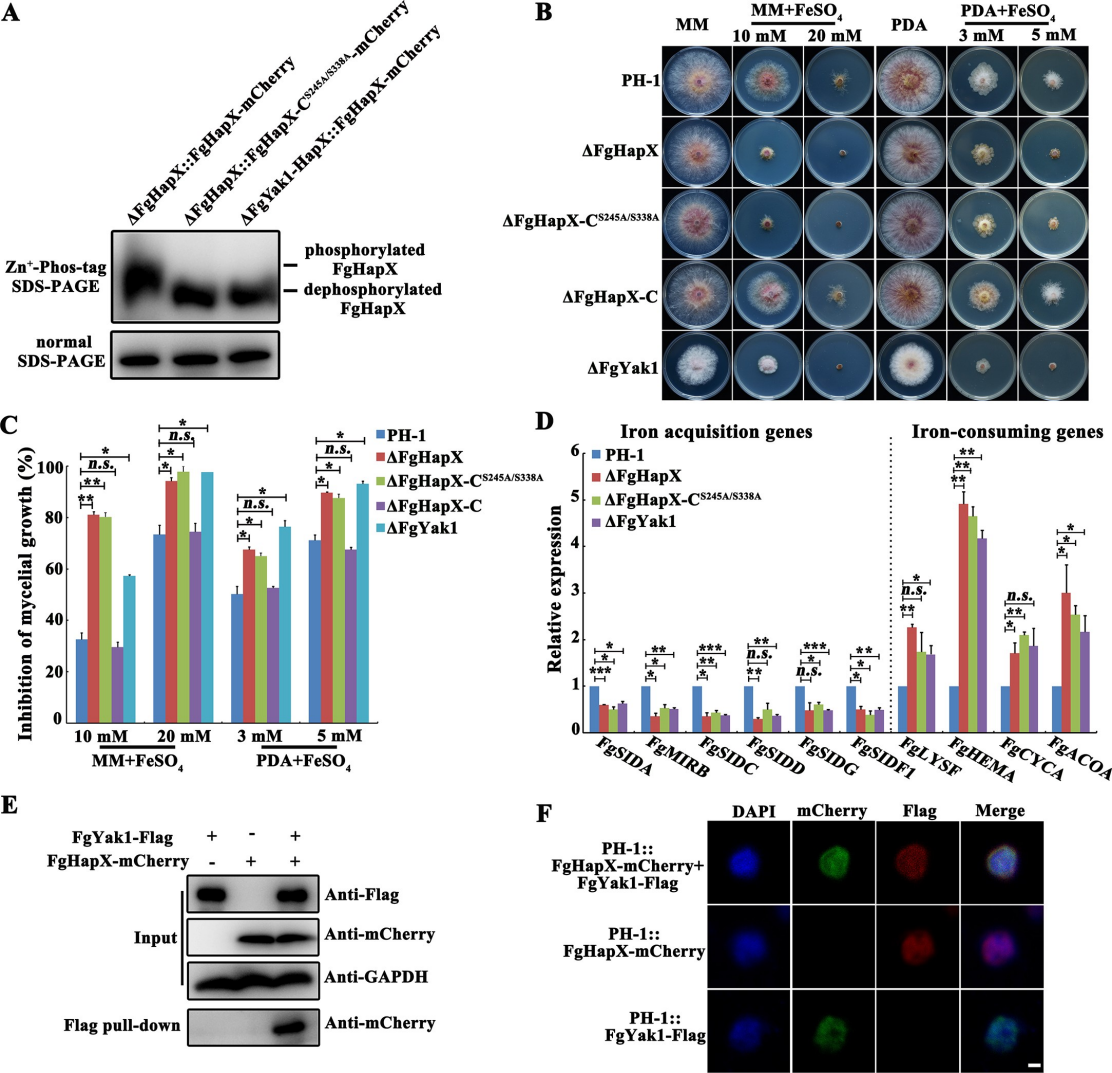
The phosphorylation of FgHapX mediated by the Ser/Thr kinase FgYak1 is required for the transcriptional activity of FgHapX. (A) The mutations of FgHapX at 245 and 338 from Ser to Ala or loss of FgYak1 led to a decreased phosphorylation level of FgHapX. Proteins extracted from ΔFgHapX::FgHapX-mCherry, ΔFgHapX::FgHapX-C^S245A/S338A^-mCherry, or ΔFgYak1-HapX::FgHapX-mCherry were subjected to Phos-tag SDS-PAGE and normal SDS-PAGE followed by immunoblotting with an anti-mCherry antibody. (B) Sensitivity of the wild type, ΔFgHapX, ΔFgHapX-C, ΔFgHapX-C^S245A/S338A^ and ΔFgYak1 to FeSO_4_. Each strain was inoculated on MM or PDA amended without or with FeSO_4_ at the indicated concentration, and then incubated at 25°C for 3 days. (C) Mycelial growth inhibition of each strain under each treatment was calculated after 3 days of incubation (B). Means and standard errors were calculated from three repeats. Significance was measured using unpaired *t*-test (*n*.*s*. not significant, *p < 0.05, **p < 0.01). (D) Relative transcription levels of iron-related genes in the wild type, ΔFgHapX, ΔFgHapX-C^S245A/S338A^, and ΔFgYak1 growth in CM for 1 day. The expression level of each gene in each mutant is the amount of mRNA relative to the same gene in the wild type. Means and standard errors of each gene were calculated from three repeats. Significance was measured using unpaired *t*-test (*n*.*s*. not significant, *p < 0.05, **p < 0.01, ***p < 0.001). (E) FgHapX interacts with FgYak1 in the co-immunoprecipitation (Co-IP) assay. Western blots of total proteins (input) from transformants expressing the FgYak1-Flag and/or FgHapX-mCherry constructs (upper panel), and the proteins eluted from anti-Flag agarose beads (lower panel) were detected with the anti-mCherry or anti-Flag antibody. Detection with the anti-GAPDH antibody was conducted for the protein loading reference. (F) FgYak1-Flag (red) interacts with FgHapX-mCherry (green) in the nucleus in the immunofluorescence assay. Nuclei were stained with DAPI. Bar = 1 μm.

In eukaryotes, phosphorylation of transcription factors frequently has been found to regulate their activities. Phosphoproteome assay showed that FgHapX contains two predicted Ser residues at 245 and 338 sites that may be subject to phosphorylation (S8 Fig). To confirm the function of these two residues, we constructed a strain containing a constitutive dephosphorylated FgHapX isoform. Briefly, the two phosphorylated Ser residues were replaced by alanine, the mutated FgHapX^S245A/S338A^ was transformed into ΔFgHapX and the resulting strain was designated as ΔFgHapX-C^S245A/S338A^. Next, we performed a phos-tag assay to detect the phosphorylation level of FgHapX in ΔFgHapX-C and in ΔFgHapX-C^S245A/S338A^. As shown in Fig 7A, the dephosphorylated level of FgHapX in ΔFgHapX-C^S245A/S338A^ was significantly higher than that in ΔFgHapX-C. To further explore the function of FgHapX phosphorylation, we determined the sensitivity of ΔFgHapX-C^S245A/S338A^ to iron stress. As shown in Fig 7B and 7C, similar to ΔFgHapX, ΔFgHapX-C^S245A/S338A^ still remained highly sensitivity to iron stress. Consistently, the qRT-PCR assays showed the expression levels of iron acquisition genes were reduced and the transcription of iron-consuming genes was elevated in ΔFgHapX-C^S245A/S338A^ (Fig 7D). In addition, these mutations did not change the quantity and localization of FgHapX (S9A and S9B Fig). Collectively, these results indicate that phosphorylation of FgHapX is required for regulating expression of iron-related genes.

To identify the potential kinase that phosphorylates FgHapX, we screened 96 kinase mutants and found that the mutant of Ser/Thr protein kinase FgYak1 (FGSG_05418) showed dramatically increased sensitivity to iron stress (Fig 7B and S10 Fig). Furthermore, co-immunoprecipitation (Co-IP) confirmed that FgYak1 interacted with FgHapX (Fig 7E). Immunofluorescence assay also revealed that FgYak1 interacted with FgHapX in the nucleus (Fig 7F). The qRT-PCR assays showed that, similar to those in ΔFgHapX and ΔFgHapX-C^S245A/S338A^, the transcription levels of iron acquisition genes were reduced and those of iron-consuming genes were elevated in ΔFgYak1 (Fig 7D). Importantly, the phos-tag assay revealed that the dephosphorylated level of FgHapX in ΔFgYak1 was higher than that in ΔFgHapX-C (Fig 7A). Meanwhile, the phos-tag assays showed that the phosphorylated levels of FgHapX in ΔFGSG_13318, ΔFGSG_00408, ΔFGSG_10381, ΔFGSG_06832, ΔFGSG_05734 or ΔFGSG_11812 were similar with that in ΔFgHapX-C, although these kinase mutants also showed elevated sensitivity to iron stress (S10A–S10D Fig). In addition, deletion of FgYak1 did not alter the quantity and localization of FgHapX (S9A and S9B Fig). Collectively, these results indicated that FgYak1 phosphorylates FgHapX in *F*. *graminearum*.

## Discussion

**Fig 8:**
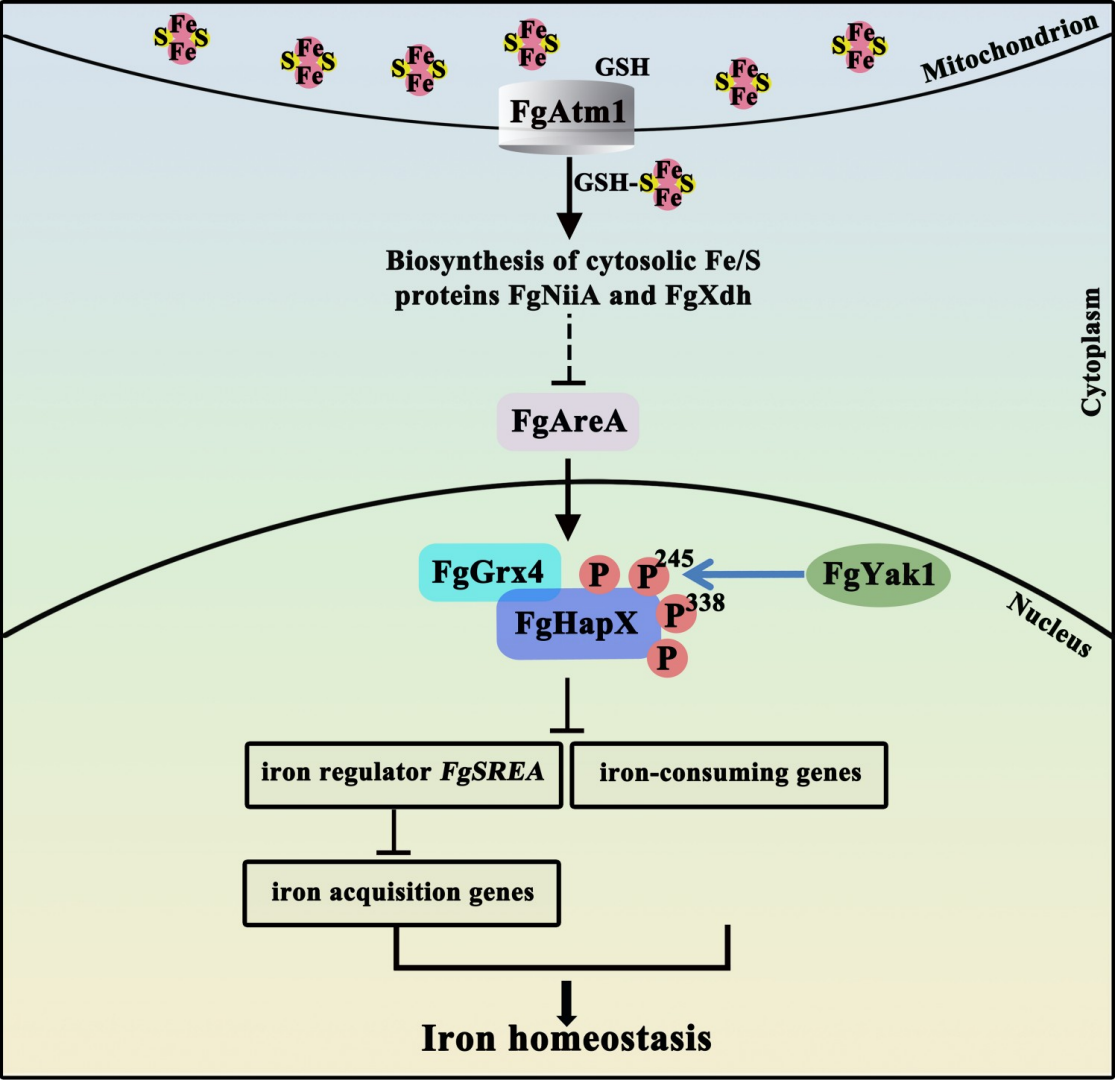
A proposed model of iron homeostasis regulated by FgAtm1 in *F*. *graminearum*. FgAtm1 regulates the export of GSH-linked [2Fe-2S] clusters from mitochondria to cytoplasm, subsequently affects the assembly of cytoplasmic Fe-S proteins nitrite reductase (FgNiiA) and xanthine dehydrogenase (FgXdh) that are key enzymes for non-preferred nitrogen utilization. The activated FgNiiA and FgXdh inhibit the transcription of nitrogen metabolism regulator FgAreA. FgAreA activates expression of the transcription repressor FgHapX, subsequently regulates the transcription of iron-related genes in *F*. *graminearum*. In addition, the function of FgHapX is dependent on its interacting protein FgGrx4 and phosphorylation mediated by kinase FgYak1.

In *S*. *cerevisiae*, the Atm1-mediated iron regulation has been well characterized. The depletion of Atm1 impedes the loading of GSH-linked [2Fe-2S] clusters onto monothiol glutaredoxins, subsequently disrupting formation of the Grx3/4-Fra1/2 complex, which results in the failure of Aft1/2 dissociation from the promoters of iron acquisition genes [27–30]. The iron acquisition genes are therefore activated constitutively in the Atm1-depleted cells. In this study, we found that lack of FgAtm1 also leads to an overload of intracellular iron. However, the regulation mechanism of iron homeostasis mediated by FgAtm1 in *F*. *graminearum* is different from what is known in *S*. *cerevisiae*. We uncovered that deletion of FgAtm1 impedes the activity of cytosolic Fe-S proteins nitrite reductase and xanthine dehydrogenase, which conversely activates the nitrogen metabolism regulator FgAreA (Fig 8). Subsequently, FgAreA activates the transcription of repressor FgHapX via binding the *FgHAPX* promoter (Fig 8). Moreover, we found that FgHapX directly represses the transcription of iron-consuming genes, but also activates the expression of iron acquisition genes indirectly via suppressing the transcription of another repressor *FgSREA* (Fig 8). It is worthy to note that *S*. *cerevisiae* does not contain a HapX ortholog, and *F*. *graminearum* and other filamentous fungi do not have the yeast Aft1/2 orthologs. These results indicate that the regulatory networks of iron homeostasis can be distinct in different fungi.

AreA belongs to the GATA nitrogen regulator and is required for the transcription of genes responsible for the utilization of non-preferred nitrogen sources in several fungi [54]. Previous studies have found that the transcription of *AREA* is induced by nitrogen starvation or the treatment with nitrate in *A*. *nidulans* [55], *F*. *graminearum* [56, 57] and *Fusarium fujikuroi* [58]. In this study, the lack of FgAtm1 compromised the activity of nitrite reductase FgNiiA and xanthine dehydrogenase FgXdh resulting in utilization defects of the non-preferred nitrogen sources nitrite and hypoxanthine, and subsequently induced the transcription of *FgAREA*. This finding indicates that iron metabolism is able to affect nitrogen utilization via the Fe-S proteins in the filamentous fungus *F*. *graminearum*.

HapX is an important transcriptional repressor of intracellular iron homeostasis in filamentous fungi [59]. In *A*. *fumigatus*, HapX not only represses genes involved in iron-consuming pathways to spare iron, but also activates iron acquisition genes to acquire iron via suppressing another repressor SreA during iron starvation [50], which is consistent with our finding. However deletion of HapX homologs does not change the transcription of iron acquisition genes in *A*. *nidulans*, *F*. *oxysporum* and *C*. *albicans* [49, 60, 61]. Previous studies have found that the transcription factor SreA may also represses the expression of HapX during iron overload [49, 62]. In *Cryptococcus neoformans*, carbon metabolism regulator Mig1 promotes the transcription of *HAPX* under low-iron conditions [63]. In this study, we found however that FgHapX is regulated by the GATA transcription factor FgAreA. To our knowledge, this is the first observation that the iron regulator HapX is regulated by a nitrogen metabolism regulator AreA in filamentous fungi.

In the current study, we also found that the functions of Grx4 orthologs vary dramatically in *F*. *graminearum* and yeasts. First, monothiol glutaredoxin FgGrx4 is required for the transcriptional activity of FgHapX via its interaction with FgHapX in *F*. *graminearum*. In yeasts, however, Grx4 homologs combine with the GSH-linked [2Fe-2S] clusters and then interact with transcription factors *S*. *cerevisiae* Aft1/Aft2 or *Schizosaccharomyces pombe* Php4/ Fep1 to disassociate these factors from the promoters of iron regulation genes [28–30, 64–66]. Second, the interaction of FgGrx4 and FgHapX is independent of the presence of FgAtm1, and the TRX domain of FgGrx4 doesn’t interact with FgHapX. In *S*. *cerevisiae*, the interaction of Grx4 and Aft1/2 is dependent on Atm1 [30]. In *S*. *pombe*, the interaction of GRX domain of Grx4 with Php4 or Fep1 is dependent on iron conditions, whereas the TRX domain continuously binds to Php4 or Fep1 [65, 66]. Third, similar to *FgHAPX* deletion, deletion of *FgGRX4* did not change intracellular iron content (S7A Fig). However, lack of Grx3/4 leads to iron accumulation in *S*. *cerevisiae* cells [52]. Fourth, deletion of *FgGRX4* caused the down-regulation of iron acquisition genes in *F*. *graminearum*. However, deletion of *GRX3* or *GRX4* causes constitutive expression of iron acquisition genes in *S*. *cerevisiae* [52, 53]. In *S*. *pombe*, *GRX4* disruption causes constitutive transcription of iron acquisition genes regulated by Fep1, or constitutive transcription of iron-consuming genes regulated by Php4 [64, 66]. Taken together, these results indicate that the function of FgGrx4 in *F*. *graminearum* is dramatically different from that of Grx4 in the yeasts.

Phosphorylation of transcription factors mediated by various kinases has been found to modulate their localization, protein accumulation and DNA binding ability in eukaryotic organisms [67]. In mammals, phosphorylation of the organismal lifespan-related transcription factor FOXO by serum and glucocorticoid-induced kinase (SGK) results in the exclusion from the nucleus and repression of transcriptional activity [68]. In *A*. *thaliana*, multisite light-induced phosphorylation of phy-interacting basic Helix Loop Helix (bHLH) transcription factor PIF3 causes its degradation [69]. In the fission yeast, phosphorylation of sterol biosynthesis regulator SpSre1 mediated by casein kinase Hhp2 reduces its protein quantity by accelerating its degradation [70]. Whereas in *Lotus japonicus*, phosphorylation of a root nodule development-associated transcriptional activator CYCLOPS by calcium- and calmodulin-dependent kinase (CCaMK) increases its DNA binding activity at the target gene promoters [71]. In this study, we discovered that phosphorylation of FgHapX is required for its transcription activity, but not for its quantity and localization (Fig 6D, S9A and S9B Fig). Furthermore, we identified that FgHapX is subject to phosphorylation mediated by the kinase FgYak1. Previous studies have reported that the Ser/Thr protein kinase Yak1 controls cell growth in response to glucose depletion by negatively regulating the cAMP-PKA pathway in *S*. *cerevisiae*, and regulates the emergence and maintenance of hyphal growth of *C*. *albicans* [72, 73]. To our knowledge, it is the first report on involvement of a kinase (Yak1) in regulating iron homeostasis by phosphorylation of a transcription factor in fungi.

In *A*. *thaliana*, Atm1 ortholog Atm3 was found to regulate the assembly of cytoplasmic molybdenum cofactor (Moco) proteins, besides Fe-S proteins [Bernard et al., 2009]. The precursor of Moco cyclic pyranopterin monophosphate (cPMP) that is synthesized in mitochondria was reported as another substrate of Atm3 [25, 74]. In the current study, we also found that *F*. *graminearum* FgAtm1 modulates the assembly of Moco protein nitrate reductase (FgNiaD) (S11 Fig). Further, we also found that *F*. *graminearum* FgAtm1 modulates mitochondrial function and redox balance besides iron homeostasis, which are in agreement with the studies in these reports of *S*. *cerevisiae* and *C*. *neoformans* [41, 75, 76]. The ΔFgAtm1 mutant displayed decreased sensitivity to the mitochondrial respiratory inhibitors diphenylene iodonium (DPI) and rotenone (complex I) and antimycin A (complex III) (S12A Fig). The mutant also exhibited increased reactive oxygen species (ROS) content and elevated sensitivity to hydrogen peroxide (H_2_O_2_) (S12B–S12D Fig), since mitochondrial respiratory complexes I and III are known to be major generators of ROS in eukaryotic cells [77]. In addition, phenotypic determination showed that deletion of *FgATM1* led to the defects in asexual and sexual development, virulence and secondary metabolite production (S13 Fig). The phenotypic defects might result from the imbalance of nutrient, iron and redox and impaired mitochondrial functions in ΔFgAtm1.

## Materials and methods

### Fungal strains and sensitivity determination

*F*. *graminearum* wild-type strain PH-1 was used as a parental strain for transformation experiments in this study. Mycelial growth of the wild type and the resulting transformants were assayed on potato dextrose agar (PDA) or minimal medium (MM) as described previously [78, 79]. To determine sensitivity to iron stress, 5-mm mycelial plugs of each strain taken from a 3-day-old colony edge were inoculated on PDA or MM supplemented without/with Fe^2+^, H_2_O_2,_ or catalytic product of each Fe-S protein, and then incubated at 25°C for 3 days in the dark. Three biological replicates were used for each strain and each experiment was repeated three times independently.

### Construction of gene replacement, GFP-, Flag-, CYFP-, NYFP- and mCherry- fusion cassettes

The double-joint PCR approach [80] was used to generate the gene replacement construct for each target gene. Briefly, for each gene, 5’ and 3’ flanking regions were amplified with the primer pairs listed in S1 Table and the resulting amplified sequences were then fused with the hygromycin resistance gene cassette (*HPH*) driven by the constitutive trpC promoter which was amplified from the pBS-HPH1 vector [81]. Protoplast transformation of *F*. *graminearum* was carried out using the protocol described previously [82]. Putative gene deletion mutants were identified by PCR assays with relevant primers (S1 Table) and the *FgATM1* deletion mutation was further confirmed by Southern hybridization assays (S3 Fig).

To construct the FgAtm1-GFP cassette, *FgATM1* containing the promoter region and open-reading frame (without the stop codon) was amplified with the relevant primers (S1 Table). The resulting PCR products were co-transformed with XhoI-digested pYF11 containing a geneticin resistance gene (*NEO*) [83] into the yeast strain XK1-25 [84] using the Alkali-Cation Yeast Transformation Kit (MP Biomedicals, Solon, USA) to generate the recombined FgAtm1-GFP fusion vector. Subsequently, the FgAtm1-GFP fusion vector was recovered from the yeast transformant using the Yeast Plasmid Kit (Solarbio, Beijing, China) and then transferred into *Escherichia coli* strain DH5α for amplification. Using the similar strategy, FgYak1 (FGSG_05418)—and FgTri1 (FGSG_00071) -GFP fusion cassettes were constructed. Using the similar strategy, FgGrx4 (FGSG_01317)—and FgYak1-Flag fusion cassettes were constructed by co-transformation with XhoI-digested PHZ126 vector. Similarly, FgHapX-CYFP and FgGrx4-NYFP fusion cassettes were constructed by co-transformation with XhoI-digested PHZ68 and PHZ65 vectors, respectively.

The double-joint PCR approach [80] was also used to construct FgHapX-, FgHapX^S245A/S338A^- and FgLeu1 (FGSG_09589)-mCherry cassettes. Briefly, the target gene containing the promoter region and open-reading fragment and the geneticin resistance gene (*NEO*) were amplified, and then fused with *mCherry* fragment. Before protoplast transformation, each fusion construct was verified by DNA sequencing. The transformation of *F*. *graminearum* was carried out using the previously described protocol [82]. All the mutants generated in this study were preserved in 15% glycerol at −80°C.

### Determination of iron content and siderophore

For determination of mitochondrial and total iron content, About 50 mg of fresh mycelia were lysed by 2% cellulase (Ryon Biological Technology CO, Ltd, Shanghai, China), 2% lysozyme (Ryon Biological Technology CO, Ltd, Shanghai, China) and 0.2% driselase from *Basidiomycetes* sp. (Sigma, St. Louis, MO, USA) for 4–6 hours. After filtration with funnel and filter paper, the filtrate was centrifuged at 5000 *g* at 4°C for 10 min. The protoplast was used for total iron determination and mitochondrial extraction with a Cell Mitochondria Isolation Kit (Beyotime Industrial Co., Ltd., Shanghai, China). Iron content was determined using the colorimetric ferrozine-based assay previously reported, with ferrozine as chelator and ferric chloride as a standard [28, 29, 85]. Briefly, aliquots (100 μl) of cell lysates were mixed with 100 μl of 10 mM HCl (the solvent of the iron standard FeCl_3_), and 100 μl of the iron-releasing reagent (a freshly mixed solution of equal volumes of 1.4 M HCl and 4.5% (w/v) KMnO_4_ in H_2_O). The mixtures were incubated within a fume hood at 60°C for 2 h. After the mixtures had cooled to room temperature, 30 μl of the iron-detection reagent (6.5 mM ferrozine, 6.5 mM neocuproine, and 2.5 M ammonium acetate and 1 M ascorbic acid) was added to each tube. After 30 min, 200 μl of the solution in each tube was transferred into a well of a 96-well plate and the absorbance was measured at 550 nm on a microplate reader. The linear range of the ferrozine assay is from 0.2 to 30 nmol.

To determine the content of extra- and intracellular siderophores, each strain was cultured in CM for 36 hours, then transferred to MM lack of FeSO_4_ and amended 0.3 mM iron-specific chelating agent bathophenanthroline disulfonate (BPS) at 25°C for 8 hours. After filtration with funnel and filter paper, the supernatant was used for determining the content of extracellular siderophore, and the mycelia was used for determining the content of intracellular siderophore. Finely ground mycelia (50 mg) were resuspended in 50 mM potassium phosphate buffer. After vigorous vortexing, the cellular debris was pelleted, and the supernatant was determined for the content of intracellular siderophore. An aliquot of supernatant (1 ml) was mixed with 1 ml chrome azurol S (CAS) assay solution that was prepared. After incubation in the dark for 1 hour at room temperature, the absorbance of each sample was measured at 630 nm and the relative content of siderophore was calculated according to previous study [86].

### Conidiation, pathogenicity and DON biosynthesis assays

For the conidiation assay, fresh mycelia (50 mg) of each strain were inoculated in a 50-ml flask containing 20 ml of carboxymethyl cellulose (CMC) liquid medium. The flasks were incubated at 25°C for 4 days in a shaker (180 rpm). Subsequently, the number of conidia in each flask was determined using a hemacytometer. Three biological replicates were used for each strain and each experiment was repeated three times independently.

Virulence assays on wheat spikelets, corn silks, and wheat seedling leaves were conducted as described previously [35]. For virulence on wheat spikelets, a 10 μl aliquot of conidial suspension (10^5^ conidia/ml) was injected into a floret in the central section spikelet of a single flowering wheat head of susceptible cultivar Jimai 22. Fifteen days after inoculation, the infected spikelets in each inoculated wheat head were recorded. For virulence on corn silks and wheat seedling leaves, a 5-mm mycelial plug of each strain was inoculated on the middle of corn silks and wheat seedling leaves, and then cultured for 4 days. Ten replicates were used for each strain and each experiment was repeated three times independently. To determine DON production, each strain was grown in liquid trichothecene biosynthesis induction (TBI) medium at 28°C for 3 d in a shaker (150 rpm) in the dark. A DON Quantification Kit (Wise Science, Zhenjiang, China) was used to quantify the DON production for each sample. The experiment was repeated three times.

### Microscopic examinations

The colony edge of each strain cultured on PDA plates at 25°C for 7 days was examined with a Leica TCS SP5 imaging system (Leica Microsystems, Wetzlar, Germany). Morphology of conidia cultured in CMC liquid medium for 4 days was observed with a Leica TCS SP5 imaging system after staining with the cell wall-damaging agent calcofluor white (CFW) at 10 μg/ml. Germinating conidia in TBI liquid medium or iron-depleted TBI liquid medium were observed with a Leica TCS SP5 imaging system. Fluorescence signals were examined with a Zeiss LSM780 confocal microscope (Gottingen, Niedersachsen, Germany). For the observation of proteins tagged with GFP, mCherry or YFP, each strain was cultured in CM at 25°C for 24 h in a shaker (180 rpm) before staining with MitoTracker or 4’,6-diamidino-2-phenylindole (DAPI) [87]. For examination of iron content, each strain was stained with 5 μM FeRhoNox-1 (Goryo Chemical, Inc., Sapporo, Japan) [88–91] for 1 hours after culture in complete medium (CM) for 36 hours. For the observation of FgTri1-GFP, each strain was cultured on wheat seedling leaves at 25°C with 100% humidity for two days.

### Determination of enzyme activity and ROS content

The wild type, ΔFgAtm1 and the ΔFgAtm1-C were grown in CM for 36 hours, and then were harvested after rinsing 3 times with sterile water. To determine FgLeu1 activity in cytosol, 50 mg of finely ground mycelia was resuspended in 1 ml of lysis buffer (1 M Tris-HCl pH 7.4, 1 M NaCl, 0.5 M EDTA, 1% Triton 100) for 10 min. The lysate was centrifuged at 14,000 *g* for 10 min at 4°C. Each protein sample (50 μg) was used for FgLeu1 activity determination. DL-threo-3-isopropylmalic acid (Wako Pure Chemical Industries, Ltd, Japan) was used as the substrate and β-isopropylmalate formation was measured by monitoring absorbance at 235 nm for 10 minutes [92, 93]. To determine FgAco1 and FgFum1 activity in cytosol and mitochondrion, the separation of mitochondrion and cytosol (without nuclei) was conducted according to the above method described for iron content determination. Each protein sample (50 μg) was used for FgAco1 activity determination. Cis-aconitic acid (Sigma-Aldrich, St. Louis, MO, USA) was used as the substrate and monitor absorbance at 340 nm for 2–4 minutes [92]. FgFum1 activity of each protein sample (50 μg) was determined with a Fumarase Specific Activity Assay Kit ab110043 (Abcam, Cambridge, UK) according to the manufacturer’s instruction. These experiments were repeated three times independently.

To examine ROS content, fresh mycelia of each strain grown in CM liquid medium for 36 hours were harvested after rinsing 3 times with sterile water. 50 mg of finely ground mycelia were resuspended in 1 ml of extraction buffer. After homogenization with a vortex shaker, the lysate was centrifuged at 14,000 *g* in a microcentrifuge for 10 min at 4°C. An aliquot of 10 μl supernatant was used for ROS determination with ROS ELISA Kit (Tong Wei Industrial Co., Ltd., Shanghai, China). Meantime, to examine ROS, each strain cultured on PDA plates at 25°C for 3 days was stained with 0.05% (wt/vol) nitroblue tetrazolium (NBT) for 2 h. These experiments were all repeated three times.

### RNA extraction and quantitative reverse transcription PCR (qRT-PCR)

Total RNA isolation from mycelia of each sample with the TaKaRa RNAiso Reagent (TaKaRa Biotechnology, Dalian, China), and reverse transcription was performed with a HiScript II 1st Strand cDNA Synthesis Kit (Vazyme Biotech, Nanjing, China). The expression level of each gene was determined by qRT-PCR with HiScript II Q RT SuperMix (Vazyme Biotech, Nanjing, China). The expression of the *FgACTIN* gene was performed as a reference. The experiment was repeated three times.

### Chromatin immunoprecipitation (ChIP)-qPCR analyses

ChIP was performed as previously described [94, 95] with additional modifications. Briefly, fresh mycelia were cross-linked with 1% formaldehyde for 15 min and then stopped with 125 mM glycine. The cultures were ground with liquid nitrogen and re-suspended in the lysis buffer (250 mM, HEPES pH 7.5, 150 mM NaCl, 1 mM EDTA, 1% Triton, 0.1% DeoxyCholate, 10 mM DTT) and protease inhibitor (Sangon Co., Shanghai, China). The DNA was sheared into ~300 bp fragments with twenty pulses of 10 s and 20 s of resting at 35% amplitude (Qsonica*sonicator, Q125, Branson, USA). After centrifugation, the supernatant was diluted with 10×ChIP dilution buffer (1.1% Triton X-100, 1.2 mM EDTA, 16.7 mM Tris–HCl, pH 8.0 and 167 mM NaCl). Immunoprecipitation was performed using monoclonal anti-GFP ab290 (Abcam, Cambridge, UK) antibody together with the protein A agarose (Santa Cruz, CA, USA) respectively. DNA was immunoprecipitated by ethanol after washing, eluting, reversing the cross-linking and digesting with proteinase K. Further, ChIP-enriched DNA was used for quantitative PCR analysis using SYBR green I fluorescent dye detection with the relative primers (S1 Table). Relative enrichment of each gene was determined by quantitative PCR and calculated first by normalizing the amount of a target cDNA fragment against that of *FgACTIN* as an internal control, and then by normalizing the value for the immunoprecipitated sample against that for the input. The ChIP-qPCR was independently repeated three times.

### Electrophoretic mobility shift assay (EMSA) assays

The cDNA encoding the N terminal 1–230 amino acids of FgHapX (FgHapX^N1–230^) was amplified and cloned into pGEX-4T-3 vector to generate GST-tagged protein. The resulting construct was transformed into the *E*. *coli* strain BL21 (DE3) after verifying the cDNA sequence. The recombinant GST-FgHapX^N1–230^ was purified by Ni sepharose beads and eluted by reduced glutathione. Promter DNAs were amplified using the primers in Table S1. For EMSA, Reaction mixtures containing purified GST -FgHapX^N1–230^, promter DNAs and 10×Binding buffer (100 mM Tris-HCl (PH 7.5), 0.5 M NaCl, 10 mM DTT, 10 mM EDTA, 50% glycerol) were incubated for 20 min at 25°C. The purified GST was used as negative controls. The reactions were electrophoresed on 1.2% agarose gel in 0.5×TAE for 45 min in 80 V under low temperature. Signals were detected in J3-3000 imaging system after dying DNA dye ethidium bromide (EB) for 15 min. The experiment was conducted independently three times.

### Yeast two-hybrid (Y2H) assays

To construct plasmids for Y2H analyses, the coding sequence of each tested gene was amplified from the cDNA of PH-1 with primer pairs indicated in S1 Table. The cDNA of each gene was inserted into the yeast GAL4-binding domain vector pGBKT7 (Clontech, Mountain View, CA, USA) and GAL4 activation domain vector pGADT7 (Clontech, Mountain View, CA, USA), respectively. The pairs of Y2H plasmids were cotransformed into *S*. *cerevisiae* strain Y2H Gold lithium acetate/single-stranded DNA/polyethylene glycol transformation protocol. In addition, a pair of plasmids, pGBKT7-53 and pGADT7 and another pair of plasmids, pGBKT7-Lam and pGADT7, served as a positive control and negative controls, respectively. Transformants were grown at 30°C for 3 d on synthetic medium (SD) lacking Leu and Trp, and then transferred to SD stripped of Ade, His, Trp and Leu to assess binding activity. Three independent experiments were performed to confirm yeast two-hybrid assay results.

To search for FgHapX-interacting proteins, we performed Y2H screens. FgHapX was cloned into the yeast vector pGBKT7. A *F*. *graminearum* cDNA library was constructed in the Y2H vector pGADT7 using total RNA extracted from mycelia and conidia. The Y2HGold that was co-transformed with the cDNA library as well as FgHapX-pGBKT7 were directly selected using SD-Trp-Leu-His. Approximately 150 potential yeast transformants containing cDNA clones interacting with FgHapX were further confirmed in selection medium SD-Trp-Leu-His-Ade.

### Western blotting assay

For western blotting assay, protein samples of strains were prepared and extracted as described previously [96]. Proteins separated on the SDS-PAGE gel were transferred onto a polyvinylidene fluoride membrane with a Bio-Rad electroblotting apparatus. The polyclonal anti-Flag A9044 (Sigma, St. Louis, MO, USA), monoclonal anti-GFP ab32146 (Abcam, Cambridge, MA, USA) and monoclonal anti-mCherry ab125096 (Abcam, Cambridge, UK) antibodies were used at a 1:2000 to 1:10000 dilution for immunoblot assays. The samples were also detected with the monoclonal anti-GAPDH antibody EM1101 (Hangzhou HuaAn Biotechnology co., Ltd.) as a reference. Incubation with a secondary antibody and chemiluminescent detection were performed as described previously [97]. The experiment was repeated three times independently.

### Co-immunoprecipitation (Co-IP) assays

The mCherry and Flag fusion constructs were verified by DNA sequencing and transformed in pairs into PH-1. Transformants expressing the fusion constructs were confirmed by western blot analysis. In addition, the transformants bearing a single fusion construct were used as references. For Co-IP assays, total proteins were extracted and incubated with the anti-Flag (Abmart, Shanghai, China) agarose overnight at 4°C as described previously [97]. Proteins eluted from agarose were analyzed by western blotting detection with the monoclonal anti-mCherry ab125096 (Abcam, Cambridge, UK) antibody. The protein samples were also detected with monoclonal anti-GAPDH antibody EM1101 (Hangzhou HuaAn Biotechnology co., Ltd.) as a reference.

### Bimolecular fluorescence complementation (BiFC) assay

The FgHapX-CYFP and FgGrx4-NYFP fusion constructs were generated by cloning the related fragments into pHZ68 and pHZ65 vectors, respectively. FgHapX-CYFP and FgGrx4-NYFP constructs were co-transformed into PH-1 and ΔFgAtm1. In addition, a pair of constructs, FgHapX-CYFP and NYFP and another pair of constructs, FgGrx4-NYFP and CYFP were used as negative controls. Transformants resistant to both hygromycin and zeocin were isolated and confirmed by PCR. YFP signals were examined with a Zeiss LSM780 confocal microscope (Gottingen, Niedersachsen, Germany).

### Phosphoproteome assay

Proteins of the wild-type PH-1 were extracted in the lysis buffer (8 M urea, 50 mM Tris 8.0, 1% NP40, 1% sodium deoxycholate, 5 mM dithiothreitol, 2 mM EDTA, 30 mM nicotinamide, 3 μm trichostatin A, 1% protease inhibitor Cocktail and 1% phosphatase inhibitor cocktail). For trypsin digestion, the protein sample was diluted in 0.1 M TEAB (triethylammonium bicarbonate), and digested with 1/25 trypsin (Promega, Madison, USA) for 12 h at 37°C. The digestion was terminated with 1% TFA (trifluoroacetic acid), and the resulting peptides were cleaned with a Strata X C18 SPE column (Phenomenex, Torrance, USA) and vacuum-dried in a scanvac maxi-beta (Labogene, Lynge, Denmark). Then, the resulting peptides (2 mg per sample) were reconstituted in 120 μl 0.5 M TEAB, and treated with a TMTsixplex label reagent kit (Pierce, Idaho, USA). Both fractionations were performed with an XBridge Shield C18 RP column (Waters, Milford, USA) in a LC20AD HPLC system (Shimadzu, Kyoto, Japan). For phosphorylation enrichment, peptides were dissolved in 80% ACN/6% TFA and then incubated with IMAC-Ti4+ beads (Sachtopore, Sachtleben Chemie, Germany) at room temperature. The beads were washed once with 50% ACN/0.5%TFA/200 mM NaCl and once with 50% ACN/0.1% TFA. The bound peptides were then eluted with 10% NH_4_OH and 80% ACN/2% FA (formic acid). All of the eluted fractions were combined, vacuum-dried and cleaned with C18 ZipTips (Millipore, Billerica, USA) according to the manufacturer’s instructions, followed by LC-MS/MS analysis according to a previous study [98]. The database search and bioinformatics analyses were performed as described previously [99].

### Phos-tag assay analysis

For Phos-tag assay, the FgHapX-mCherry fusion construct was transferred into the ΔFgHapX and ΔFgYak1 mutants and the FgHapX^S245A/S338A^-mCherry fusion construct was transferred into the ΔFgHapX mutant. Protein samples were prepared and extracted as described above. Each resulting protein sample was loaded on 8% SDS-polyacrylamide gels prepared with 25 μM Phos binding reagent acrylamide (APE×BIO, F4002) and 100 μM ZnCl_2_. Gels were electrophoresed at 20 mA/gel for 3–5 h. Prior to protein transfer, gels were first equilibrated three times in transfer buffer containing 5 mM EDTA for 5 min and further equilibrated in transfer buffer without EDTA for 5 min for two times. Protein transfer from the Zn^2+^-phos-tag acrylamide gel to the PVDF membrane was performed for 4–5 h at 100 V on ice, and finally the membrane was analyzed by western blotting with the monoclonal anti-mCherry ab125096 (Abcam, Cambridge, UK) antibody.

### Immunofluorescence assays

FgYak1-Flag and FgHapX-mCherry were co-transformed into the wild type. The nuclei of corresponding strains were extracted as described previously [100] and then fixed on the polylysine slides. Slides were incubated with the polyclonal anti-Flag antibody A9044 (Sigma, St. Louis, MO) and monoclonal anti-mCherry ab125096 (Abcam, Cambridge, UK) antibody at 1:500 dilution, respectively, followed by the secondary antibodies Andy Fluor 594 goat anti-mouse L119A (red fluorescence) (GeneCopoeia, Maryland, US) and Andy Fluor 488 goat anti-rabbit L110A (green fluorescence) (GeneCopoeia, Maryland, US) at 1:300 dilution. Nuclei were stained with DAPI for 15 minutes before fluorescence observation.

## Supporting information

S1 Fig Sensitivity of 60 ABC transporter deletion mutants of *F*. *graminearum* to FeSO_4_. A 5-mm mycelial plug of each strain was inoculated on MM without or with 8 mM FeSO_4_, and then incubated at 25°C for 3 days (A) or 7 days (B).
(TIF)

S2 Fig Subcellular localization of FgAtm1- or FgAtm1^N1-111^-GFP in the *in situ* complemented strain. (A) PCR analyses of the wild type, ΔFgAtm1 and the ectopically and *in situ* complemented strains. (B) Colocalization of FgAtm1- or FgAtm1^N1-111^-GFP with mitochondrial dye MitoTracker. The plasmid FgAtm1- or FgAtm1^N1-111^-GFP was transformed *in situ* into ΔFgAtm1 and the resulting strain was then examined with a fluorescent microscope after MitoTracker staining. Bar = 10 μm.
(TIF)

S3 Fig Construction and identification of *FgATM1* gene deletion and complemented mutants. (A) Gene replacement strategy for the *FgATM1* deletion mutant ΔFgAtm1. The hygromycin resistance cassette (HPH) is denoted by the large grey arrow. (B) PCR analyses of the wild type, ΔFgAtm1 and the complemented strain ΔFgAtm1-C. (C) Southern blot hybridization analysis of the wild type and ΔFgAtm1 using an upstream fragment of *FgATM1* as a probe (left panel). Southern blot hybridization analysis of the wild type and the complemented strain ΔFgAtm1-C using a G418 fragment as a probe (right panel).
(TIF)

S4 Fig The lack of FgAtm1 did not alter the localization and protein quantity of FgLeu1-mCherry. (A) Comparison in fluorescence intensity of the cytoplasmic Fe-S protein isopropyl malate isomerase FgLeu1 between the wild type and ΔFgAtm1. Bar = 10 μm. (B) Comparison in FgLeu1 quantity between the wild type and ΔFgAtm1. Western blots of proteins obtained from transformants of PH-1 and ΔFgAtm1 bearing the FgLeu1-mCherry were detected with the monoclonal anti-mCherry antibody. Detection with the anti-GAPDH antibody was the loading reference. Band intensities were quantified with the program IMAGE QUANT TL. The intensity of FgLeu1 band for each strain is relative to that of the GAPDH band.
(TIF)

S5 Fig Seven potential cytoplasmic Fe-S proteins are not involved in response to iron stress in *F*. *graminearum*. Sensitivity of the five Fe-S proteins deletion mutants to FeSO_4_. A 5-mm mycelial plug of each strain was inoculated on PDA without or with 3 mM FeSO_4_, and then incubated at 25°C for 3 days. Means and standard errors were calculated from three repeats. Significance was measured using an unpaired *t*-test (*n*.*s*. not significant).
(TIF)

S6 Fig Relative transcription level of *FgHAPX* in the wild type and ΔFgAtm1 growth in CM for 1 day. The expression level of *FgHAPX* in PH-1 was set to 1. Means and standard errors were calculated from three repeats. Significance was measured using an unpaired *t*-test (*p < 0.05).
(TIF)

S7 Fig Deletion of FgGrx4 did not alter intracellular iron concentration, the localization, or protein quantity of FgHapX. (A) Total iron content of the wild type and ΔFgGrx4 was determined by colorimetric ferrozine-based assay after growth in CM at 25°C for 36 hours. Means and standard errors were calculated from three repeats. Significance was measured using an unpaired *t*-test (*n*.*s*. not significant). (B and C) The localization (B) and protein quantity (C) of FgHapX in the wild type and ΔFgGrx4. Bar = 10 μm.
(TIF)

S8 Fig Mass spectrum data of two identified phosphorylated sites in FgHapX. (A) Identification of two serine phosphorylation sites (S245 and S338) in FgHapX. (B) Sketch map of the above two residues in FgHapX.
(TIF)

S9 Fig The phosphorylation of FgHapX did not alter its localization or protein quantity. The localization (A) and protein quantity (B) of FgHapX in the wild type, ΔFgHapX-C^S245A/S338A^ mutated in the two phosphorylation sites and ΔFgYak1. Bar = 10 μm.
(TIF)

S10 Fig Sensitivity determination of 96 kinase deletion mutants of *F*. *graminearum* to FeSO_4_. (A and B) A 5-mm mycelial plug of each strain was inoculated on MM without or with 10 mM FeSO_4_, and then incubated at 25°C for 3 days (A) or 7 days (B). (C) Mycelial growth inhibition of seven kinase mutants was calculated after 3-day-incubation at the conditions (A). Means and standard errors were calculated from three repeats. Significance was measured using an unpaired *t*-test (*p < 0.05, **p < 0.01). (D) Phos-tag assays for the seven kinase mutants. Protein extracted from each strain was subjected to Phos-tag SDS-PAGE and normal SDS-PAGE followed by immunoblotting with an anti-mCherry antibody.
(TIF)

S11 Fig FgAtm1 regulates the assembly of cytosolic Moco protein nitrate reductase (FgNiaD). Each strain was cultured in CM at 25°C for 36 hours before activity determination. The FgNiaD activity of the wild type was set as 1. Means and standard errors were calculated from three repeats. Significance was measured using unpaired *t*-test (*n*.*s*. not significant, **p < 0.01).
(TIF)

S12 Fig FgAtm1 modulates mitochondrial function and intracellular redox balance. (A) Sensitivity of the wild type and ΔFgAtm1 to different mitochondrial complex inhibitors. A 5-mm mycelial plug of each strain was inoculated on PDA without or with diphenylene iodonium (DPI) and rotenone (complex I), antimycin A (complex III), malonic acid (complex II) or 6-Hydroxydopamine hydrobromide (6-OHDA) (complex IV) at the indicated concentration, and then incubated at 25°C for 3 days. Mycelial growth inhibition of each treatment was calculated after 3-day-incubation. Means and standard errors were calculated from three repeats. Significance was measured using an unpaired *t*-test (*n*.*s*. not significant, **p < 0.01). (B) Reactive oxygen species (ROS) production in the wild type, ΔFgAtm1 and ΔFgAtm1-C. The 3-day-old colony of each strain was stained with 0.05% (wt/vol) ROS indicator nitroblue tetrazolium (NBT) for 2 h with or without 50 μM DPI and 4 μM antimycin A. (C) Quantitative determination of ROS in the wild type, ΔFgAtm1 and ΔFgAtm1-C with or without the treatment of 50 μM DPI and 4 μM antimycin A. Means and standard errors were calculated from three repeats. Significance was measured using an unpaired *t*-test (*n*.*s*. not significant, *p < 0.05, **p < 0.01). (D) Sensitivity of the wild type, ΔFgAtm1 and ΔFgAtm1-C to hydrogen peroxide (H_2_O_2_). A 5-mm mycelial plug of each strain was inoculated on PDA amended without or with 12 mM H_2_O_2_ and then incubated at 25°C for 3 days.
(TIF)

S13 Fig The deletion of *FgATM1* led to defects in asexual and sexual development, virulence and secondary metabolite production. (A) Conidial morphology of the wild type, ΔFgAtm1, and ΔFgAtm1-C. The differential interference contrast (DIC) images of conidia from each strain were captured with an electronic microscope. Bar = 20 μm. Conidia were cultured in CMC liquid medium at 25°C for 4 days in a shaker. (B) Conidia of each strain were quantified after incubation in CMC for 4 days. Means and standard errors were calculated from three repeats. Significance was measured using an unpaired t-test (n.s. not significant, ***p < 0.001). (C and D) Comparisons of conidium length (C) and septum number (D) among the above strains. Conidia were cultured in CMC liquid medium at 25°C for 4 days in a shaker. A total of 200 conidia were examined for each strain. (E) Perithecia of the wild type, ΔFgAtm1 and ΔFgAtm1-C were grown on carrot agar for induction of perithecial formation. Bar = 500 μm. (F) The amount of DON produced by the wild type, ΔFgAtm1 and ΔFgAtm1-C after incubation in trichothecene biosynthesis induction (TBI) medium for 3 days. The dry weight of mycelium is used as an internal reference. Means and standard errors were calculated from three repeats. Significance was measured using an unpaired t-test (n.s. not significant, ***p < 0.001). (G) Relative expression levels of DON biosynthetic genes FgTRI1, FgTRI5, and FgTRI6 in the wild type, ΔFgAtm1, and ΔFgAtm1-C after growth in TBI at 28°C in the dark for 3 days. Means and standard errors were calculated from three repeats. Significance was measured using an unpaired t-test (n.s. not significant, ***p < 0.001). (H) The subcellular localization of FgTri1-GFP. FgTri1-GFP localized to “toxisomes” in PH-1, whereas, the Tri1-GFP labelled toxisomes were nearly undetectable in ΔFgAtm1. Bar = 10 μm. Each strain was examined in artificially wounded wheat seedling leaves 48 h after inoculation. (I) FgTri1-GFP was dramatically reduced in ΔFgAtm1 determined by western blotting with the monoclonal anti-GFP antibody. The protein samples were also detected with the monoclonal anti-GAPDH antibody as a reference. (J) Comparison of red pigment production among PH-1, ΔFgAtm1 and ΔFgAtm1-C. Each strain was grown on PDA for 7 days (left panel) or in potato dextrose broth (PDB) for 2 days (right panel).
(TIF)

S1 Table The primers used in this study. (DOCX)

S2 Table Serial analysis of gene expression (SAGE) for identification of differential expression (>2-folds) genes in *FgATM1* deletion mutant as compared with those in the wild type. (DOCX)

S3 Table A list of putative FgHapX-interacting proteins identified by the yeast two-hybrid assay. (DOCX)

## References

[1] Aisen P, Enns C, Wessling-Resnick M. 2001 Chemistry and biology of eukaryotic iron metabolism. Int J Biochem Cell Biol 33: 940–959. 10.1016/S1357-2725(01)00063-2. 11470229

[2] Kobayashi T, Nishizawa NK. 2012 Iron uptake, translocation, and regulation in higher plants. Annu Rev Plant Biol 63: 131–152. 10.1146/annurev-arplant-042811-105522 22404471

[3] Philpott CC, Ryu MS. 2014 Special delivery: distributing iron in the cytosol of mammalian cells. Front Pharmacol 5: 173 10.3389/fphar.2014.00173 25101000 PMC4106451

[4] Huma N, Salim UrR, Anjum FM, Murtaza MA, Sheikh MA. 2007 Food fortification strategy-preventing iron deficiency anemia: a review. Crit Rev Food Sci Nutr 47: 259–265. 10.1080/10408390600698262 17453923

[5] Zimmermann MB, Hurrell RF 2007 Nutritional iron deficiency. The Lancet 370: 511–520. 10.1016/S0140-6736(07)61235-5. 17693180

[6] Oide S, Moeder W, Krasnoff S, Gibson D, Haas H, Yoshioka K, Turgeon BG. 2006 NPS6, encoding a nonribosomal peptide synthetase involved in siderophore-mediated iron metabolism, is a conserved virulence determinant of plant pathogenic ascomycetes. Plant Cell 18: 2836–2853. 10.1105/tpc.106.045633 17056706 PMC1626607

[7] Schrettl M, Bignell E, Kragl C, Joechl C, Rogers T, Arst HN, Haynes K, Haas H. 2004 Siderophore biosynthesis but not reductive iron assimilation is essential for *Aspergillus fumigatus* virulence. J Exp Med 200: 1213–1219. 10.1084/jem.20041242 15504822 PMC2211866

[8] Ehrensberger KM, Bird AJ. 2011 Hammering out details: regulating metal levels in eukaryotes. Trends Biochem Sci 36: 524–531. 10.1016/j.tibs.2011.07.002 21840721

[9] Ganz T, Nemeth E. 2011 Hepcidin and disorders of iron metabolism. Annu Rev Med 62: 347–360. 10.1146/annurev-med-050109-142444 20887198

[10] Hentze MW, Muckenthaler MU, Galy B, Camaschella C. 2010 Two to tango: regulation of Mammalian iron metabolism. Cell 142: 24–38. 10.1016/j.cell.2010.06.028 20603012

[11] Del Sorbo G, Schoonbeek Hj, De Waard MA. 2000 Fungal transporters involved in efflux of natural toxic compounds and fungicides. Fungal Genet Biol 30: 1–15. 10.1006/fgbi.2000.1206. 10955904

[12] Klein C, Kuchler K, Valachovic M. 2011 ABC proteins in yeast and fungal pathogens. Essays Biochem 50: 101–119. 10.1042/bse0500101 21967054

[13] Perlin MH, Andrews J, Toh SS. 2014 Essential letters in the fungal alphabet: ABC and MFS transporters and their roles in survival and pathogenicity. Adv Genet 85: 201–253. 10.1016/B978-0-12-800271-1.00004-4 24880736

[14] Khandelwal NK, Kaemmer P, Forster TM, Singh A, Coste AT, Andes DR, Hube B, Sanglard D, Chauhan N, Kaur R, d'Enfert C, Mondal AK, Prasad R. 2016 Pleiotropic effects of the vacuolar ABC transporter MLT1 of *Candida albicans* on cell function and virulence. Biochem J 473: 1537–1552. 10.1042/BCJ20160024 27026051 PMC4888455

[15] Desideri E, Filomeni G, Ciriolo MR. 2012 Glutathione participates in the modulation of starvation-induced autophagy in carcinoma cells. Autophagy 8: 1769–1781. 10.4161/auto.22037 22964495 PMC3541287

[16] Ding R, Jin S, Pabon K, Scotto KW. 2016 A role for ABCG2 beyond drug transport: Regulation of autophagy. Autophagy 12: 737–751. 10.1080/15548627.2016.1155009 26983466 PMC4854550

[17] Huang H, Lu-Bo Y, Haddad GG. 2014 A *Drosophila* ABC transporter regulates lifespan. PLoS Genet 10: e1004844 10.1371/journal.pgen.1004844 25474322 PMC4256198

[18] Hwang JU, Song WY, Hong D, Ko D, Yamaoka Y, Jang S, Yim S, Lee E, Khare D, Kim K, Palmgren M, Yoon HS, Martinoia E, Lee Y. 2016 Plant ABC transporters enable many unique aspects of a terrestrial plant's lifestyle. Mol. Plant 9: 338–355. 10.1016/j.molp.2016.02.003 26902186

[19] Li J, Cowan JA. 2015 Glutathione-coordinated [2Fe-2S] cluster: a viable physiological substrate for mitochondrial ABCB7 transport. Chem Commun 51: 2253–2255. 10.1039/C4CC09175B. PMC4522903 25556595

[20] Qi W, Li J, Cowan JA. 2014 A structural model for glutathione-complexed iron-sulfur cluster as a substrate for ABCB7-type transporters. Chem Commun 50: 3795–3798. 10.1039/C3CC48239A. PMC4052440 24584132

[21] Young L, Leonhard K, Tatsuta T, Trowsdale J, Langer T. 2001 Role of the ABC transporter Mdl1 in peptide export from mitochondria. Science 291: 2135–2138. 10.1126/science.1056957 11251115

[22] Chloupková M, LeBard LS, Koeller DM. 2003 MDL1 is a high copy suppressor of ATM1: evidence for a role in resistance to oxidative stress. J Mol Biol 331: 155–165. 10.1016/s0022-2836(03)00666-1 12875842

[23] Zwiers LH, Roohparvar R, de Waard MA. 2007 MgAtr7, a new type of ABC transporter from *Mycosphaerella graminicola* involved in iron homeostasis. Fungal Genet Biol 44: 853–863. 10.1016/j.fgb.2007.02.001 17379549

[24] Bekri S, Kispal G, Lange H, Fitzsimons E, Tolmie J, Lill R, Bishop DF. 2000 Human ABC7 transporter: gene structure and mutation causing X-linked sideroblastic anemia with ataxia with disruption of cytosolic iron-sulfur protein maturation. Blood 96: 3256–3264. 11050011

[25] Bernard DG, Cheng Y, Zhao Y, Balk J. 2009 An allelic mutant series of *ATM3* reveals its key role in the biogenesis of cytosolic iron-sulfur proteins in *Arabidopsis*. Plant Physiol 151: 590–602. 10.1104/pp.109.143651 19710232 PMC2754654

[26] Kispal G, Csere P, Prohl C, Lill R. 1999 The mitochondrial proteins Atm1p and Nfs1p are essential for biogenesis of cytosolic Fe/S proteins. EMBO J 18: 3981–3989. 10.1093/emboj/18.14.3981 10406803 PMC1171474

[27] Kumanovics A, Chen OS, Li L, Bagley D, Adkins EM, Lin H, Dingra NN, Outten CE, Keller G, Winge D, Ward D, Kaplan J. 2008 Identification of FRA1 and FRA2 as genes involved in regulating the yeast iron regulon in response to decreased mitochondrial iron-sulfur cluster synthesis. J Biol Chem 283: 10276–10286. 10.1074/jbc.M801160200 18281282 PMC2447656

[28] Li H, Mapolelo DT, Dingra NN, Keller G, Riggs-Gelasco PJ, Winge DR, Johnson MK, Outten CE. 2011 Histidine 103 in Fra2 is an iron-sulfur cluster ligand in the [2Fe-2S] Fra2-Grx3 complex and is required for in vivo iron signaling in yeast. J Biol Chem 286: 867–876. 10.1074/jbc.M110.184176 20978135 PMC3013046

[29] Li H, Mapolelo DT, Dingra NN, Naik SG, Lees NS, Hoffman BM, Riggs-Gelasco PJ, Huynh BH, Johnson MK, Outten CE. 2009 The yeast iron regulatory proteins Grx3/4 and Fra2 form heterodimeric complexes containing a [2Fe-2S] cluster with cysteinyl and histidyl ligation. Biochemistry 48: 9569–9581. 10.1021/bi901182w 19715344 PMC2796373

[30] Ueta R, Fujiwara N, Iwai K, Yamaguchi-Iwai Y. 2012 Iron-induced dissociation of the Aft1p transcriptional regulator from target gene promoters is an initial event in iron-dependent gene suppression. Mol Cell Biol 32: 4998–5008. 10.1128/MCB.00726-12 23045394 PMC3510542

[31] Mühlenhoff U, Molik S, Godoy JR, Uzarska MA, Richter N, Seubert A, Zhang Y, Stubbe J, Pierrel F, Herrero E, Lillig CH, Lill R. 2010 Cytosolic monothiol glutaredoxins function in intracellular iron sensing and trafficking via their bound iron-sulfur cluster. Cell metab 12: 373–385. 10.1016/j.cmet.2010.08.001. PMC4714545 20889129

[32] Goswami RS, Kistler HC. 2004 Heading for disaster: *Fusarium graminearum* on cereal crops. Mol Plant Pathol 5: 515–525. 10.1111/j.1364-3703.2004.00252.x 20565626

[33] Pestka JJ, Smolinski AT. 2005 Deoxynivalenol: toxicology and potential effects on humans. J Toxicol Env Heal B 8: 39–69. 10.1080/10937400590889458. 15762554

[34] Kovalchuk A, Driessen AJ. 2010 Phylogenetic analysis of fungal ABC transporters. BMC genomics 11: 177 10.1186/1471-2164-11-177 20233411 PMC2848647

[35] Yin Y, Wang Z, Cheng D, Chen X, Chen Y, Ma Z. 2018 The ATP-binding protein FgArb1 is essential for penetration, infectious and normal growth of *Fusarium graminearum*. New Phytol 219: 1447–1466. 10.1111/nph.15261 29932228

[36] Gardiner DM, Osborne S, Kazan K, Manners JM. 2009 Low pH regulates the production of deoxynivalenol by *Fusarium graminearum*. Microbiology 155: 3149–3156. 10.1099/mic.0.029546-0 19497949

[37] Regev-Rudzki N, Battat E, Goldberg I, Pines O. 2009 Dual localization of fumarase is dependent on the integrity of the glyoxylate shunt. Mol Microbiol 72: 297–306. 10.1111/j.1365-2958.2009.06659.x 19415796

[38] Regev-Rudzki N, Karniely S, Ben-Haim NN, Pines O. 2005 Yeast aconitase in two locations and two metabolic pathways: seeing small amounts is believing. Mol Biol Cell 16: 4163–4171. 10.1091/mbc.E04-11-1028 15975908 PMC1196327

[39] Lill R, Muhlenhoff U. 2005 Iron-sulfur-protein biogenesis in eukaryotes. Trends Biochem Sci 30: 133–141. 10.1016/j.tibs.2005.01.006 15752985

[40] Takaya N. 2009 Response to hypoxia, reduction of electron acceptors, and subsequent survival by filamentous fungi. Biosci Biotechnol Biochem 73: 1–8. 10.1271/bbb.80487 19129650

[41] Kispal G, Csere P, Guiard B, Lill R. 1997 The ABC transporter Atm1p is required for mitochondrial iron homeostasis. FEBS Letters 418: 346–50. 10.1016/s0014-5793(97)01414-2 9428742

[42] Frey PA, Hegeman AD, Ruzicka FJ. 2008 The radical SAM superfamily. Crit Rev Biochem Mol 43: 63–88. 10.1080/10409230701829169. 18307109

[43] Mulliez E, Duarte V, Arragain S, Fontecave M, Atta M. 2017 On the role of additional [4Fe-4S] clusters with a free coordination site in radical-SAM enzymes. Front Chem 5: 17 10.3389/fchem.2017.00017 28361051 PMC5352715

[44] Berger H, Basheer A, Bock S, Reyes-Dominguez Y, Dalik T, Altmann F, Strauss J. 2008 Dissecting individual steps of nitrogen transcription factor cooperation in the *Aspergillus nidulans* nitrate cluster. Mol Microbiol 69: 1385–1398. 10.1111/j.1365-2958.2008.06359.x 18673441

[45] Giese H, Sondergaard TE, Sorensen JL. 2013 The AreA transcription factor in *Fusarium graminearum* regulates the use of some nonpreferred nitrogen sources and secondary metabolite production. Fungal Biol 117: 814–821. 10.1016/j.funbio.2013.10.006 24295920

[46] Lopez-Berges MS, Rispail N, Prados-Rosales RC, Di Pietro A. 2010 A nitrogen response pathway regulates virulence functions in *Fusarium oxysporum* via the protein kinase TOR and the bZIP protein MeaB. Plant Cell 22: 2459–2475. 10.1105/tpc.110.075937 20639450 PMC2929112

[47] Lopez-Berges MS, Schafer K, Hera C, Di Pietro A. 2014 Combinatorial function of velvet and AreA in transcriptional regulation of nitrate utilization and secondary metabolism. Fungal Genet Biol 62: 78–84. 10.1016/j.fgb.2013.11.002 24240057

[48] Hesberg C, Hansch R, Mendel RR, Bittner F. 2004 Tandem orientation of duplicated xanthine dehydrogenase genes from *Arabidopsis thaliana*: differential gene expression and enzyme activities. J Biol Chem 279: 13547–13554. 10.1074/jbc.M312929200 14726515

[49] Hortschansky P, Eisendle M, Al-Abdallah Q, Schmidt AD, Bergmann S, Thon M, Kniemeyer O, Abt B, Seeber B, Werner ER, Kato M, Brakhage AA, Haas H. 2007 Interaction of HapX with the CCAAT-binding complex–a novel mechanism of gene regulation by iron. EMBO J 26: 3157–3168. 10.1038/sj.emboj.7601752 17568774 PMC1914100

[50] Schrettl M, Beckmann N, Varga J, Heinekamp T, Jacobsen ID, Jochl C, Moussa TA, Wang S, Gsaller F, Blatzer M, Werner ER, Niermann WC, Brakhage AA, Haas H. 2010 HapX-mediated adaption to iron starvation is crucial for virulence of *Aspergillus fumigatus*. PLoS Pathog 6: e1001124 10.1371/journal.ppat.1001124 20941352 PMC2947994

[51] Schrettl M, Kim HS, Eisendle M, Kragl C, Nierman WC, Heinekamp T, Werner ER, Jacobsen I, Illmer P, Yi H, Brakhage AA, Haas H. 2008 SreA-mediated iron regulation in *Aspergillus fumigatus*. Mol Microbiol 70:27–43. 10.1111/j.1365-2958.2008.06376.x 18721228 PMC2610380

[52] Pujol-Carrion N, Belli G, Herrero E, Nogues A, de la Torre-Ruiz MA. 2006 Glutaredoxins Grx3 and Grx4 regulate nuclear localisation of Aft1 and the oxidative stress response in *Saccharomyces cerevisiae*. J Cell Sci 119:4554–64. 10.1242/jcs.03229 17074835

[53] Ojeda L, Keller G, Muhlenhoff U, Rutherford JC, Lill R, Winge DR. 2006 Role of glutaredoxin-3 and glutaredoxin-4 in the iron regulation of the Aft1 transcriptional activator in *Saccharomyces cerevisiae*. J Biol Chem 281: 17661–17669. 10.1074/jbc.M602165200 16648636

[54] Tudzynski B. 2014 Nitrogen regulation of fungal secondary metabolism in fungi. Front Microbiol 5: 656 10.3389/fmicb.2014.00656 25506342 PMC4246892

[55] Todd RB, Fraser JA, Wong KH, Davis MA, Hynes MJ. 2005 Nuclear accumulation of the GATA factor AreA in response to complete nitrogen starvation by regulation of nuclear export. Eukaryot Cell 4: 1646–1653. 10.1128/EC.4.10.1646-1653.2005 16215172 PMC1265900

[56] Min K, Shin Y, Son H, Lee J, Kim JC, Choi GJ, Lee YW. 2012 Functional analyses of the nitrogen regulatory gene *areA* in *Gibberella zeae*. FEMS Microbiol Lett 334: 66–73. 10.1111/j.1574-6968.2012.02620.x 22702217

[57] Hou R, Jiang C, Zheng Q, Wang C, Xu JR. 2015 The AreA transcription factor mediates the regulation of deoxynivalenol (DON) synthesis by ammonium and cyclic adenosine monophosphate (cAMP) signalling in *Fusarium graminearum*. Mol Plant Pathol 16: 987–999. 10.1111/mpp.12254 25781642 PMC6638501

[58] Michielse CB, Pfannmuller A, Macios M, Rengers P, Dzikowska A, Tudzynski B. 2014 The interplay between the GATA transcription factors AreA, the global nitrogen regulator and AreB in *Fusarium fujikuroi*. Mol Microbiol 91: 472–493. 10.1111/mmi.12472 24286256

[59] Hortschansky P, Haas H, Huber EM, Groll M, Brakhage AA. 2017 The CCAAT-binding complex (CBC) in *Aspergillus* species. Biochim Biophys Acta 1860: 560–570. 10.1016/j.bbagrm.2016.11.008. 27939757

[60] Lopez-Berges MS, Capilla J, Turra D, Schafferer L, Matthijs S, Jochl C, Cornelis P, Guarro J, Haas H, Di Pietro A. 2012 HapX-mediated iron homeostasis is essential for rhizosphere competence and virulence of the soilborne pathogen *Fusarium oxysporum*. Plant Cell 24: 3805–3822. 10.1105/tpc.112.098624 22968717 PMC3480304

[61] Chen C, Pande K, French SD, Tuch BB, Noble SM. 2011 An iron homeostasis regulatory circuit with reciprocal roles in *Candida albicans* commensalism and pathogenesis. Cell Host Microbe 10: 118–135. 10.1016/j.chom.2011.07.005 21843869 PMC3165008

[62] Schrettl M, Haas H. 2011 Iron homeostasis–Achilles' heel of *Aspergillus fumigatus*? Curr Opin Microbiol 14: 400–405. 10.1016/j.mib.2011.06.002 21724450 PMC3162135

[63] Caza M, Hu G, Price M, Perfect JR, Kronstad JW. 2016 The Zinc Finger Protein Mig1 Regulates Mitochondrial Function and Azole Drug Susceptibility in the Pathogenic Fungus *Cryptococcus neoformans*. mSphere 1: e00080–15. 10.1128/mSphere.00080-15. PMC4863601 27303693

[64] Jbel M, Mercier A, Labbe S. 2011 Grx4 monothiol glutaredoxin is required for iron limitation-dependent inhibition of Fep1. Eukaryot Cell 10: 629–645. 10.1128/EC.00015-11 21421748 PMC3127657

[65] Kim KD, Kim HJ, Lee KC, Roe JH. 2011 Multi-domain CGFS-type glutaredoxin Grx4 regulates iron homeostasis via direct interaction with a repressor Fep1 in fission yeast. Biochem Bioph Res Co 408: 609–614. 10.1016/j.bbrc.2011.04.069. 21531205

[66] Mercier A, Labbe S. 2009 Both Php4 function and subcellular localization are regulated by iron via a multistep mechanism involving the glutaredoxin Grx4 and the exportin Crm1. J Biol Chem 284: 20249–20262. 10.1074/jbc.M109.009563 19502236 PMC2740451

[67] Li B, Jiang S, Yu X, Cheng C, Chen S, Cheng Y, Yuan JS, Jiang D, He P, Shan L. 2015 Phosphorylation of trihelix transcriptional repressor ASR3 by MAP KINASE4 negatively regulates *Arabidopsis* immunity. Plant Cell 27: 839–856. 10.1105/tpc.114.134809 25770109 PMC4558661

[68] Webb AE, Brunet A. 2014 FOXO transcription factors: key regulators of cellular quality control. Trends Biochem Sci 39: 159–169. 10.1016/j.tibs.2014.02.003 24630600 PMC4021867

[69] Ni W, Xu SL, Chalkley RJ, Pham TN, Guan S, Maltby DA, Burlingame AL, Wang ZY, Quail PH. 2013 Multisite light-induced phosphorylation of the transcription factor PIF3 is necessary for both its rapid degradation and concomitant negative feedback modulation of photoreceptor phyB levels in *Arabidopsis*. Plant Cell 25: 2679–2698. 10.1105/tpc.113.112342 23903316 PMC3753391

[70] Brookheart RT, Lee CY, Espenshade PJ. 2014 Casein kinase 1 regulates sterol regulatory element-binding protein (SREBP) to control sterol homeostasis. J Biol Chem 289: 2725–2735. 10.1074/jbc.M113.511899 24327658 PMC3908405

[71] Singh S, Katzer K, Lambert J, Cerri M, Parniske M. 2014 CYCLOPS, a DNA-binding transcriptional activator, orchestrates symbiotic root nodule development. Cell Host Microbe 15: 139–152. 10.1016/j.chom.2014.01.011 24528861

[72] Lee P, Cho BR, Joo HS, Hahn JS. 2008 Yeast Yak1 kinase, a bridge between PKA and stress-responsive transcription factors, Hsf1 and Msn2/Msn4. Mol Microbiol 70: 882–895. 10.1111/j.1365-2958.2008.06450.x 18793336

[73] Goyard S, Knechtle P, Chauvel M, Mallet A, Prevost MC, Proux C, Coppee JY, Schwarz P, Dromer F, Park H, Filler SG, Janbon G, d'Enfert C, 2008 The Yak1 kinase is involved in the initiation and maintenance of hyphal growth in *Candida albicans*. Mol Biol Cell 19: 2251–2266. 10.1091/mbc.E07-09-0960 18321992 PMC2366847

[74] Teschner J, Lachmann N, Schulze J, Geisler M, Selbach K, Santamaria-Araujo J, Balk J, Mendel RR, Bittner F. 2010 A novel role for *Arabidopsis* mitochondrial ABC transporter ATM3 in molybdenum cofactor biosynthesis. Plant Cell 22:468–80. 10.1105/tpc.109.068478 20164445 PMC2845412

[75] Miao R, Kim H, Koppolu UM, Ellis EA, Scott RA, Lindahl PA. 2009 Biophysical characterization of the iron in mitochondria from Atm1p-depleted *Saccharomyces cerevisiae*. Biochemistry 48: 9556–68. 10.1021/bi901110n 19761223 PMC2758324

[76] Do E, Park S, Li MH, Wang JM, Ding C, Kronstad JW, Jung WH. 2017 The mitochondrial ABC transporter Atm1 plays a role in iron metabolism and virulence in the human fungal pathogen *Cryptococcus neoformans*. Med Mycol 56: 458–468. 10.1093/mmy/myx073. PMC7539791 29420779

[77] Turrens JF. 1997 Superoxide production by the mitochondrial respiratory chain. Bioscience reports 17: 3–8. 10.1023/a:1027374931887 9171915

[78] Gu Q, Chen Y, Liu Y, Zhang C, Ma Z. 2015 The transmembrane protein FgSho1 regulates fungal development and pathogenicity via the MAPK module Ste50-Ste11-Ste7 in *Fusarium graminearum*. New Phytol 206: 315–328. 10.1111/nph.13158 25388878

[79] Zhang X, Chen X, Jiang J, Yu M, Yin Y, Ma Z. 2015 The tubulin cofactor A is involved in hyphal growth, conidiation and cold sensitivity in *Fusarium asiaticum*. BMC Microbiol 15: 35 10.1186/s12866-015-0374-z 25886735 PMC4342098

[80] Yu JH, Hamari Z, Han KH, Seo JA, Reyes-Domínguez Y, Scazzocchio C. 2004 Double-joint PCR: a PCR-based molecular tool for gene manipulations in filamentous fungi. Fungal Genet Biol 41: 973–981. 10.1016/j.fgb.2004.08.001 15465386

[81] Liu XH, Lu JP, Zhang L, Dong B, Min H, Lin FC. 2007 Involvement of a *Magnaporthe grisea* serine/threonine kinase gene, *MgATG1*, in appressorium turgor and pathogenesis. Eukaryot Cell 6: 997–1005. 10.1128/EC.00011-07 17416896 PMC1951528

[82] Yu F, Gu Q, Yun Y, Yin Y, Xu JR, Shim WB, Ma Z. 2014 The TOR signaling pathway regulates vegetative development and virulence in *Fusarium graminearum*. New Phytol 203: 219–232. 10.1111/nph.12776 24684168

[83] Park G, Bruno KS, Staiger CJ, Talbot NJ, Xu JR. 2004 Independent genetic mechanisms mediate turgor generation and penetration peg formation during plant infection in the rice blast fungus. Mol Microbiol 53: 1695–1707. 10.1111/j.1365-2958.2004.04220.x 15341648

[84] Bruno KS, Tenjo F, Li L, Hamer JE, Xu JR. 2004 Cellular localization and role of kinase activity of PMK1 in *Magnaporthe grisea*. Eukaryot Cell 3: 1525–1532. 10.1128/EC.3.6.1525-1532.2004 15590826 PMC539019

[85] Riemer J, Hoepken HH, Czerwinska H, Robinson SR, Dringen R. 2004 Colorimetric ferrozine-based assay for the quantitation of iron in cultured cells. Anal Biochem 331: 370–375. 10.1016/j.ab.2004.03.049 15265744

[86] Machuca A, Milagres AMF. 2003 Use of CAS-agar plate modified to study the effect of different variables on the siderophore production by *Aspergillus*. Lett Appl Microbiol 36: 177–181. 10.1046/j.1472-765x.2003.01290.x 12581379

[87] Wang C, Zhang S, Hou R, Zhao Z, Zheng Q, Xu Q, Zheng D, Wang G, Liu H, Gao X, Ma JW, Kistler HC, Kang Z, Xu JR. 2011 Functional analysis of the kinome of the wheat scab fungus *Fusarium graminearum*. PLoS Pathog 7: e1002460 10.1371/journal.ppat.1002460 22216007 PMC3245316

[88] Hirayama T, Okuda K, Nagasawa H. 2013 A highly selective turn-on fluorescent probe for iron (II) to visualize labile iron in living cells. Chem Sci 4: 1250–1256. 10.1039/C2SC21649C.

[89] Kamihara Y, Takada K, Sato T, Kawano Y, Murase K, Arihara Y, Kikuchi S, Hayasaka N, Usami M, Iyama S, Miyanishi K, Sato Y, Kobune M, Miyanishi K, Kato J. 2016 The iron chelator deferasirox induces apoptosis by targeting oncogenic Pyk2/β-catenin signaling in human multiple myeloma. Oncotarget 7: 64330–64341. 10.18632/oncotarget.11830 27602957 PMC5325446

[90] Chaudhary K, Promsote W, Ananth S, Veeranan-Karmegam R, Tawfik A, Arjunan P, Martin P, Smith SB, Thangaraju M, Kisselev O, Ganapathy V, Gnana-Prakasam JP. 2018 Iron overload accelerates the progression of diabetic retinopathy in association with increased retinal renin expression. Sci Rep-UK 8: 3025 10.1038/s41598-018-21276-2. PMC5813018 29445185

[91] Smith M, Rimdeika A, Siow R, Naftalin R. 2018 Zinc attenuates UVA-dependent labile iron increase in human dermal fibroblasts: implications for skin ageing. Postgrad Med J 94: A7 10.1136/postgradmedj-2018-fpm.17.

[92] Pierik AJ, Netz DJ, Lill R. 2009 Analysis of iron-sulfur protein maturation in eukaryotes. Nat Protoc 4: 753–766. 10.1038/nprot.2009.39 19528951

[93] Weerapana E, Wang C, Simon GM, Richter F, Khare S, Dillon MB, Bachovchin DA, Mowen K, Baker D, Cravatt BF. 2010 Quantitative reactivity profiling predicts functional cysteines in proteomes. Nature 468: 790–795. 10.1038/nature09472 21085121 PMC3058684

[94] Nasmith CG, Walkowiak S, Wang L, Leung WW, Gong Y, Johnston A, Harris LJ, Guttman DS, Subramaniam R. 2011 Tri6 is a global transcription regulator in the phytopathogen *Fusarium graminearum*. PLoS Pathog 7: e1002266 10.1371/journal.ppat.1002266 21980289 PMC3182926

[95] Saleh A, Alvarez-Venegas R, Avramova Z. 2008 An efficient chromatin immunoprecipitation (ChIP) protocol for studying histone modifications in *Arabidopsis* plants. Nat Protoc 3: 1018–1025. 10.1038/nprot.2008.66 18536649

[96] Gu Q, Zhang C, Yu F, Yin Y, Shim WB, Ma Z. 2015 Protein kinase FgSch9 serves as a mediator of the target of rapamycin and high osmolarity glycerol pathways and regulates multiple stress responses and secondary metabolism in *Fusarium graminearum*. Environ Microbiol 17: 2661–2676. 10.1111/1462-2920.12522 24903410

[97] Liu Z, Wang Z, Huang M, Yan L, Ma Z, Yin Y. 2017 The FgSsb-FgZuo-FgSsz complex regulates multiple stress responses and mycotoxin production via folding the soluble SNARE Vam7 and beta2-tubulin in *Fusarium graminearum*. Environ Microbiol 19: 5040–5059. 10.1111/1462-2920.13968 29076607

[98] Carapito C, Klemm C, Aebersold R, Domon B. 2009 Systematic LC-MS analysis of labile post-translational modifications in complex mixtures. J Proteome Res 8: 2608–2614. 10.1021/pr800871n 19284785

[99] Jiao X, Sherman BT, Huang da W, Stephens R, Baseler MW, Lane HC, Lempicki RA. 2012 DAVID-WS: a stateful web service to facilitate gene/protein list analysis. Bioinformatics 28: 1805–1806. 10.1093/bioinformatics/bts251 22543366 PMC3381967

[100] Wang W, Ye R, Xin Y, Fang X, Li C, Shi H, Zhou X, Qi Y. 2011 An importin beta protein negatively regulates MicroRNA activity in Arabidopsis. Plant Cell 23: 3565–3576. 10.1105/tpc.111.091058 21984696 PMC3229135

