# Supplementary figures and images for: A fungal ABC transporter FgAtm1 regulates iron homeostasis via the transcription factor cascade FgAreA-HapX
Source: PLoS Pathog. 2019 Sep 23;15(9):e1007791. doi: 10.1371/journal.ppat.1007791 (PMC6788720; doi:10.1371/journal.ppat.1007791)

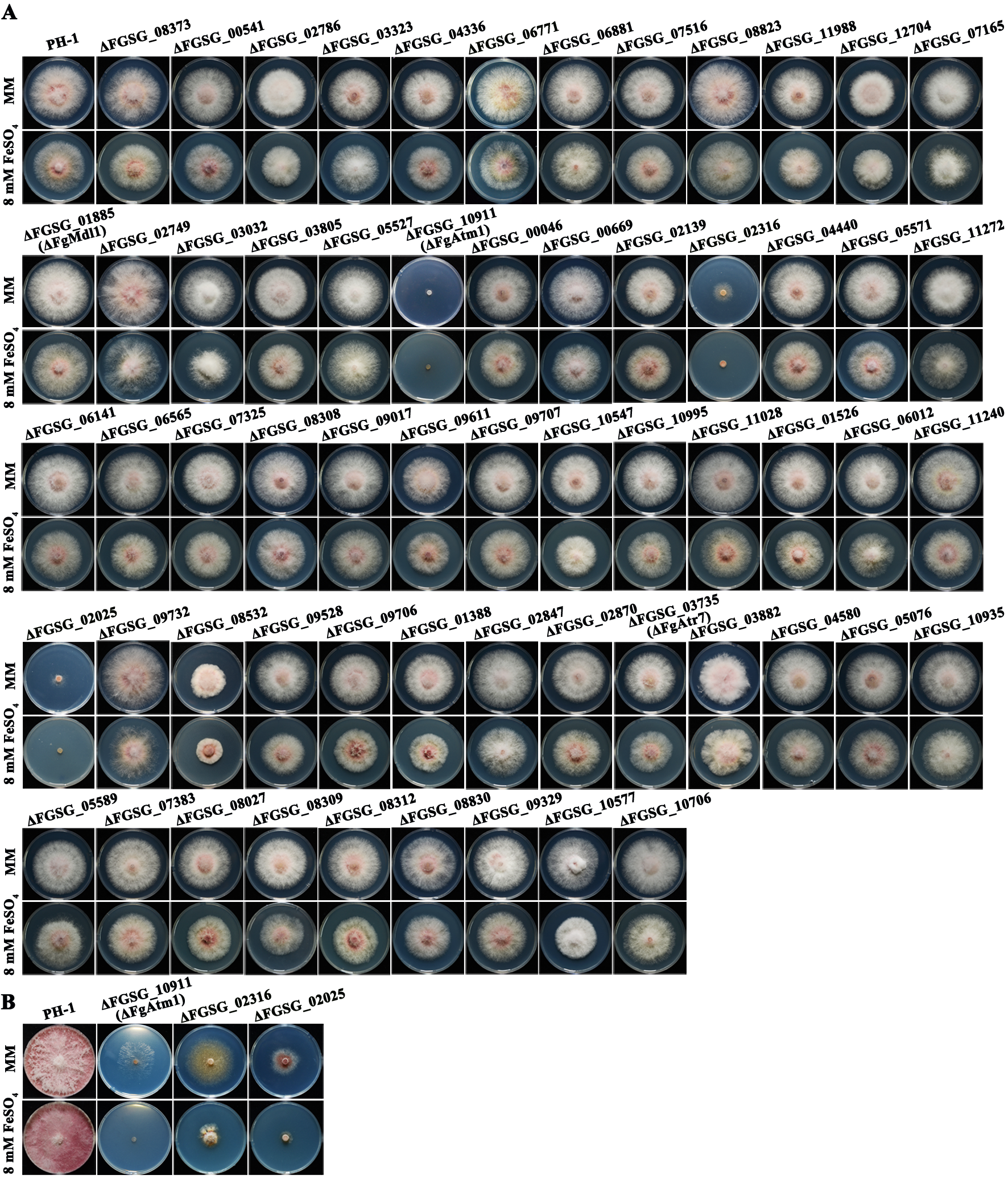

Supplement: S1 Fig — A 5-mm mycelial plug of each strain was inoculated on MM without or with 8 mM FeSO4, and then incubated at 25°C for 3 days (A) or 7 days (B). (TIF) [file ppat.1007791.s001.tif]

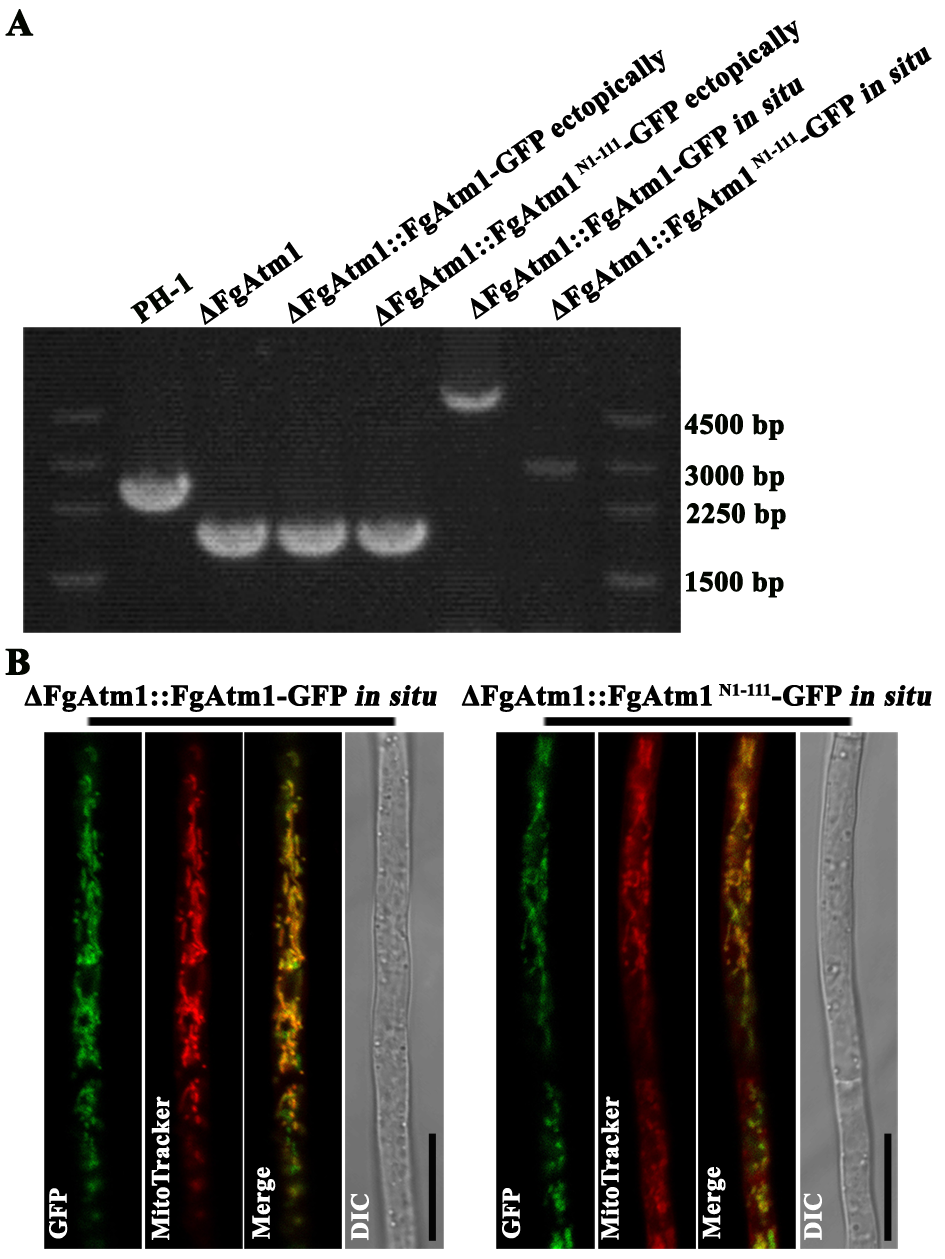

Supplement: S2 Fig — (A) PCR analyses of the wild type, ΔFgAtm1 and the ectopically and in situ complemented strains. (B) Colocalization of FgAtm1- or FgAtm1N1-111-GFP with mitochondrial dye MitoTracker. The plasmid FgAtm1- or FgAtm1N1-111-GFP was transformed in situ into ΔFgAtm1 and the resulting strain was then examined with a fluorescent microscope after MitoTracker staining. Bar = 10 μm. (TIF) [file ppat.1007791.s002.tif]

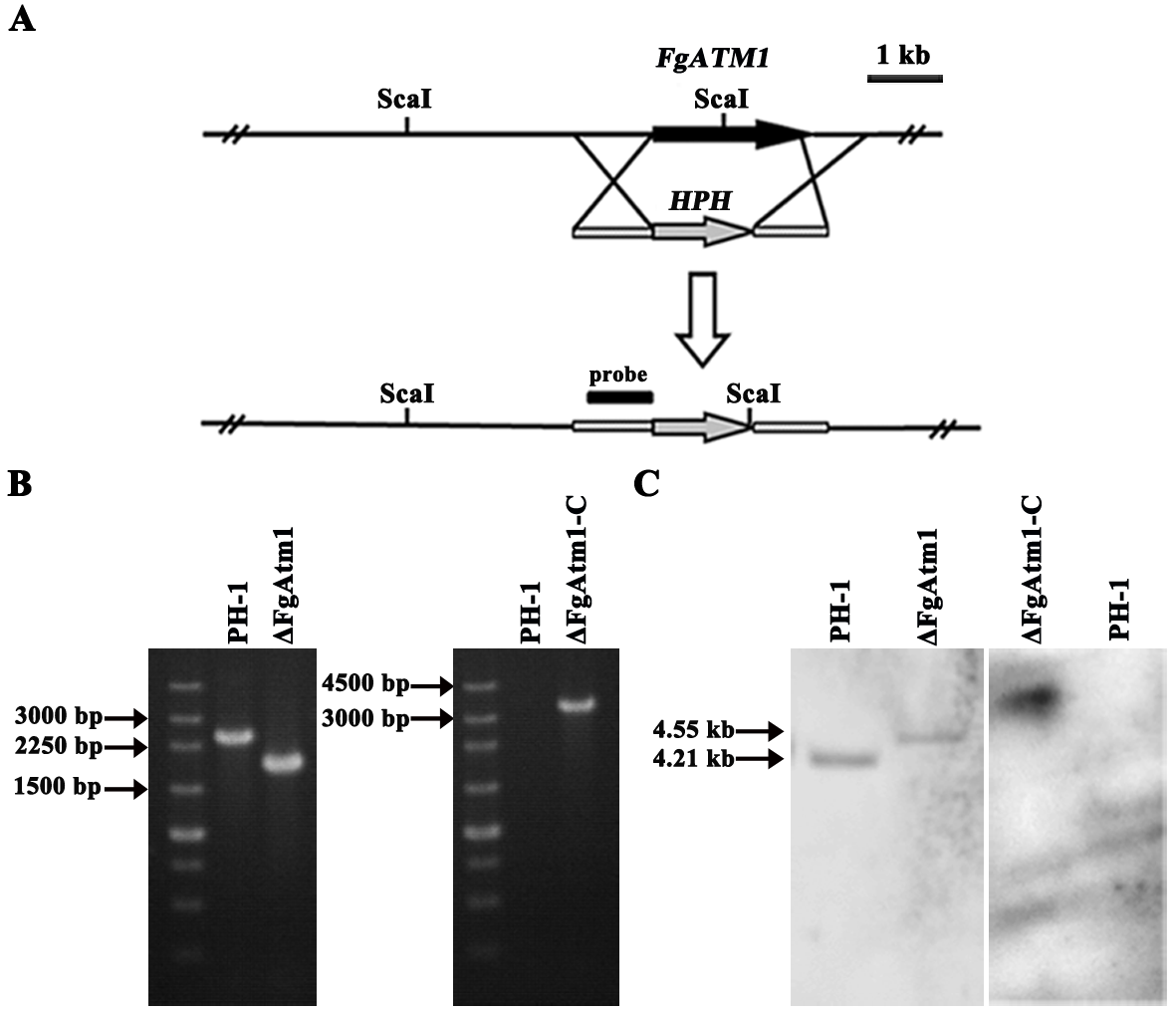

Supplement: S3 Fig — (A) Gene replacement strategy for the FgATM1 deletion mutant ΔFgAtm1. The hygromycin resistance cassette (HPH) is denoted by the large grey arrow. (B) PCR analyses of the wild type, ΔFgAtm1 and the complemented strain ΔFgAtm1-C. (C) Southern blot hybridization analysis of the wild type and ΔFgAtm1 using an upstream fragment of FgATM1 as a probe (left panel). Southern blot hybridization analysis of the wild type and the complemented strain ΔFgAtm1-C using a G418 fragment as a probe (right panel). (TIF) [file ppat.1007791.s003.tif]

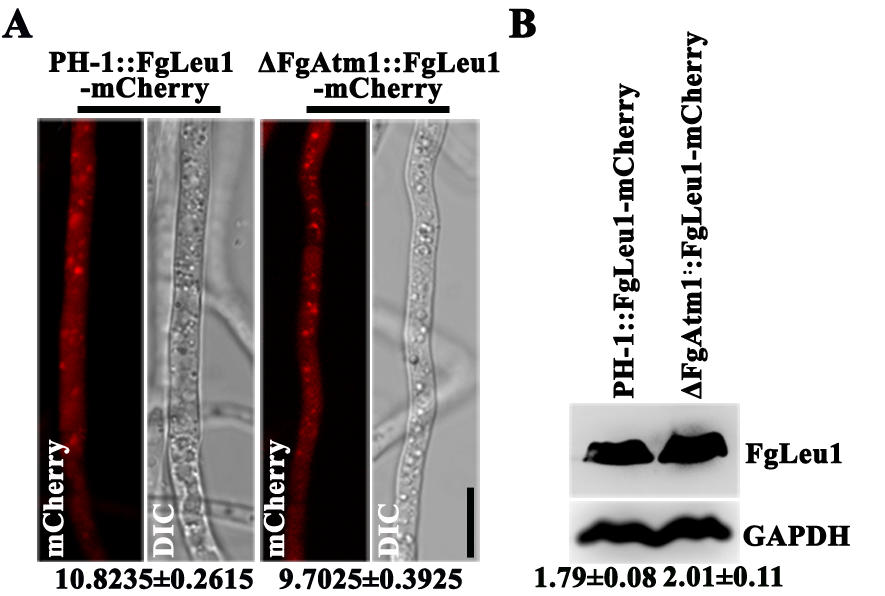

Supplement: S4 Fig — (A) Comparison in fluorescence intensity of the cytoplasmic Fe-S protein isopropyl malate isomerase FgLeu1 between the wild type and ΔFgAtm1. Bar = 10 μm. (B) Comparison in FgLeu1 quantity between the wild type and ΔFgAtm1. Western blots of proteins obtained from transformants of PH-1 and ΔFgAtm1 bearing the FgLeu1-mCherry were detected with the monoclonal anti-mCherry antibody. Detection with the anti-GAPDH antibody was the loading reference. Band intensities were quantified with the program IMAGE QUANT TL. The intensity of FgLeu1 band for each strain is relative to that of the GAPDH band. (TIF) [file ppat.1007791.s004.tif]

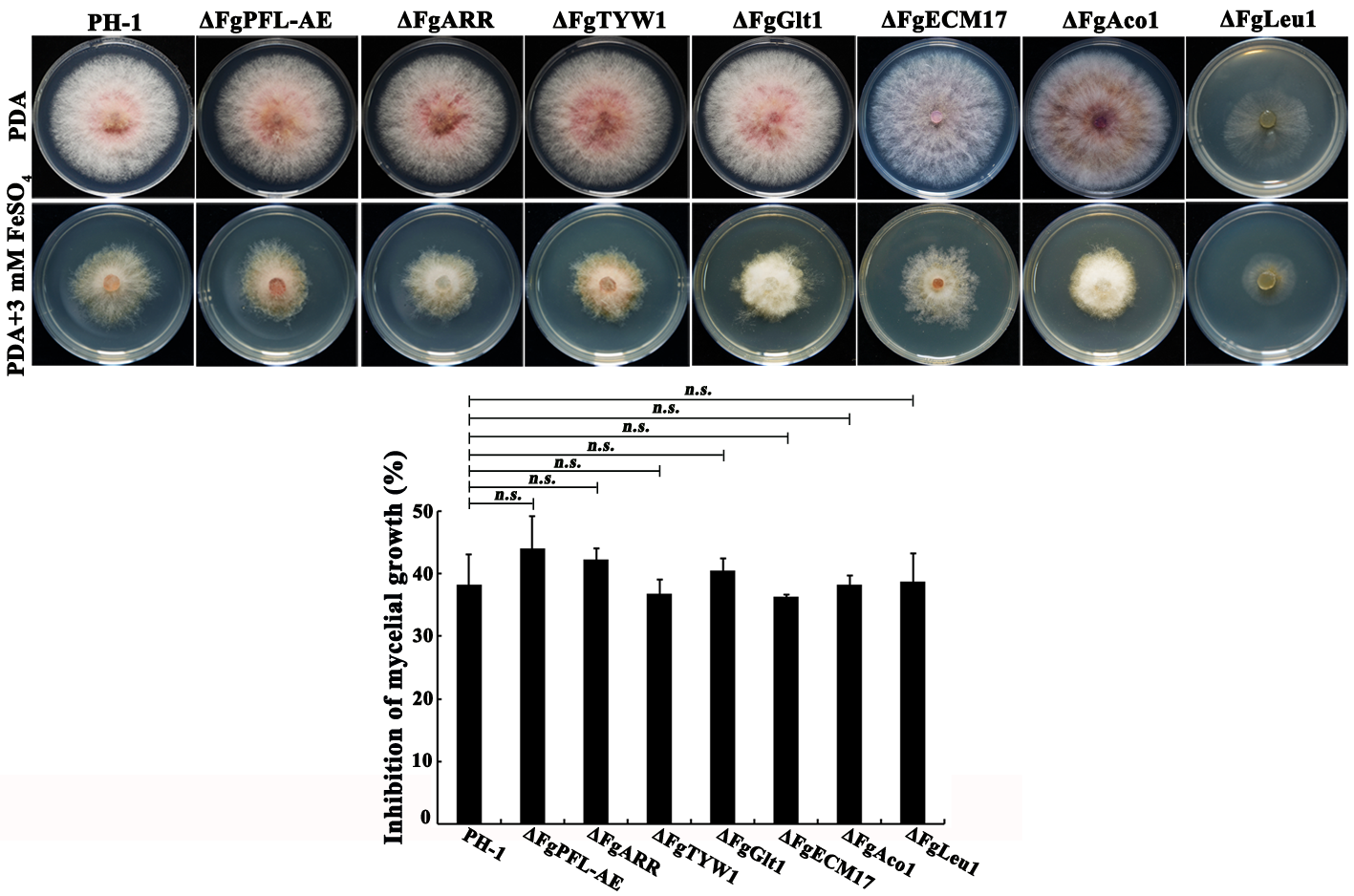

Supplement: S5 Fig — Sensitivity of the five Fe-S proteins deletion mutants to FeSO4. A 5-mm mycelial plug of each strain was inoculated on PDA without or with 3 mM FeSO4, and then incubated at 25°C for 3 days. Means and standard errors were calculated from three repeats. Significance was measured using an unpaired t-test (n.s. not significant). (TIF) [file ppat.1007791.s005.tif]

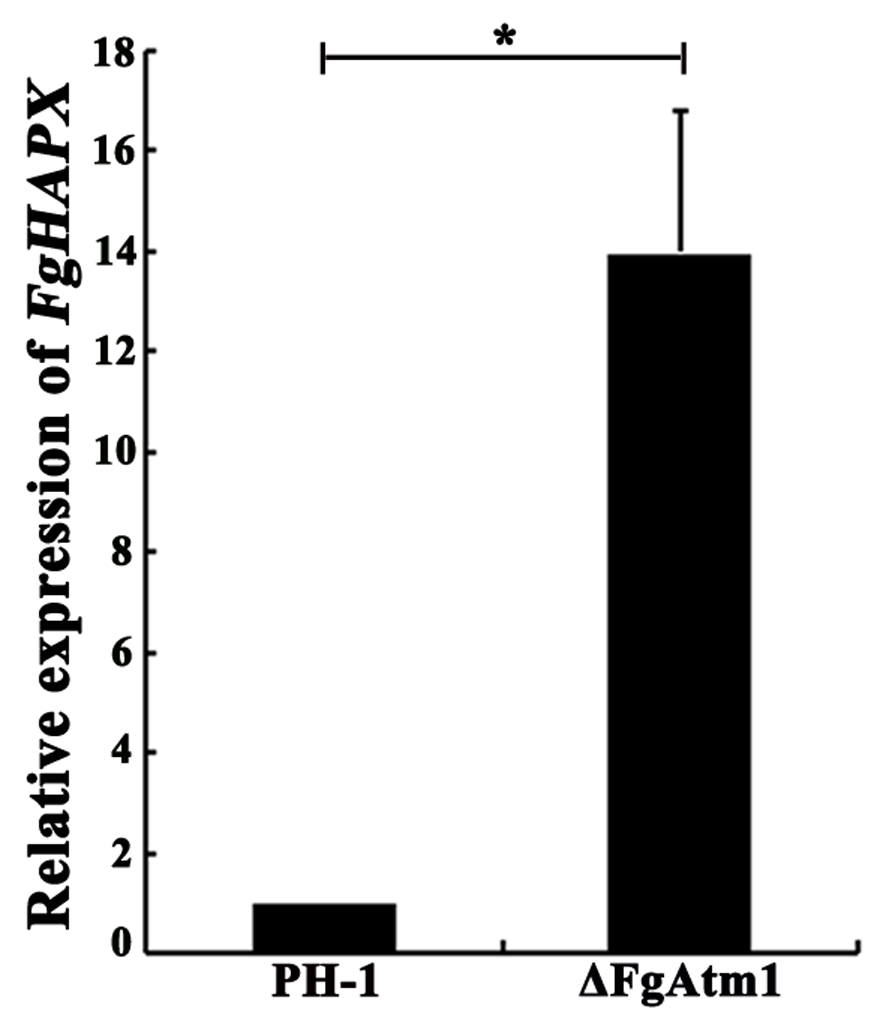

Supplement: S6 Fig — The expression level of FgHAPX in PH-1 was set to 1. Means and standard errors were calculated from three repeats. Significance was measured using an unpaired t-test (*p < 0.05). (TIF) [file ppat.1007791.s006.tif]

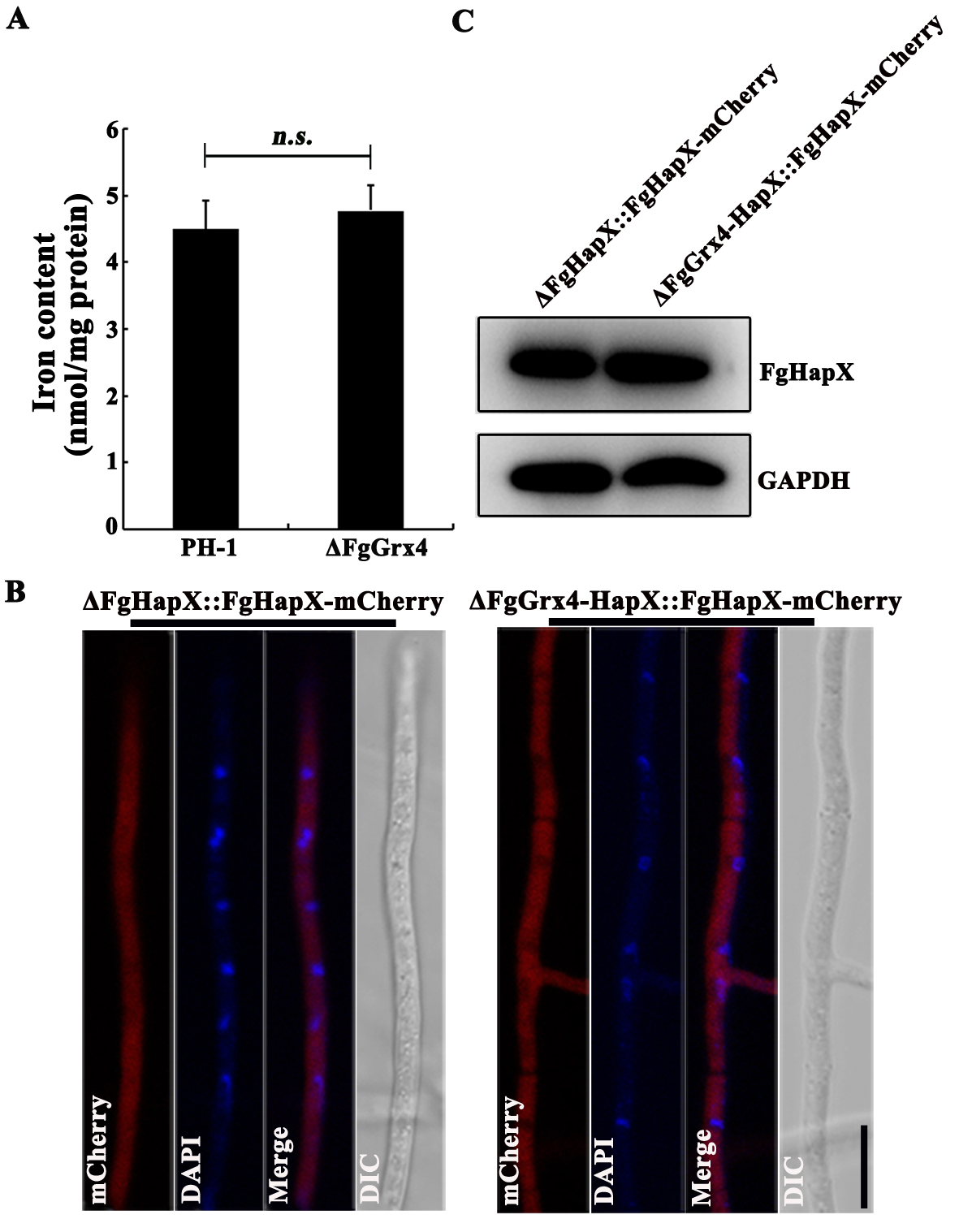

Supplement: S7 Fig — (A) Total iron content of the wild type and ΔFgGrx4 was determined by colorimetric ferrozine-based assay after growth in CM at 25°C for 36 hours. Means and standard errors were calculated from three repeats. Significance was measured using an unpaired t-test (n.s. not significant). (B and C) The localization (B) and protein quantity (C) of FgHapX in the wild type and ΔFgGrx4. Bar = 10 μm. (TIF) [file ppat.1007791.s007.tif]

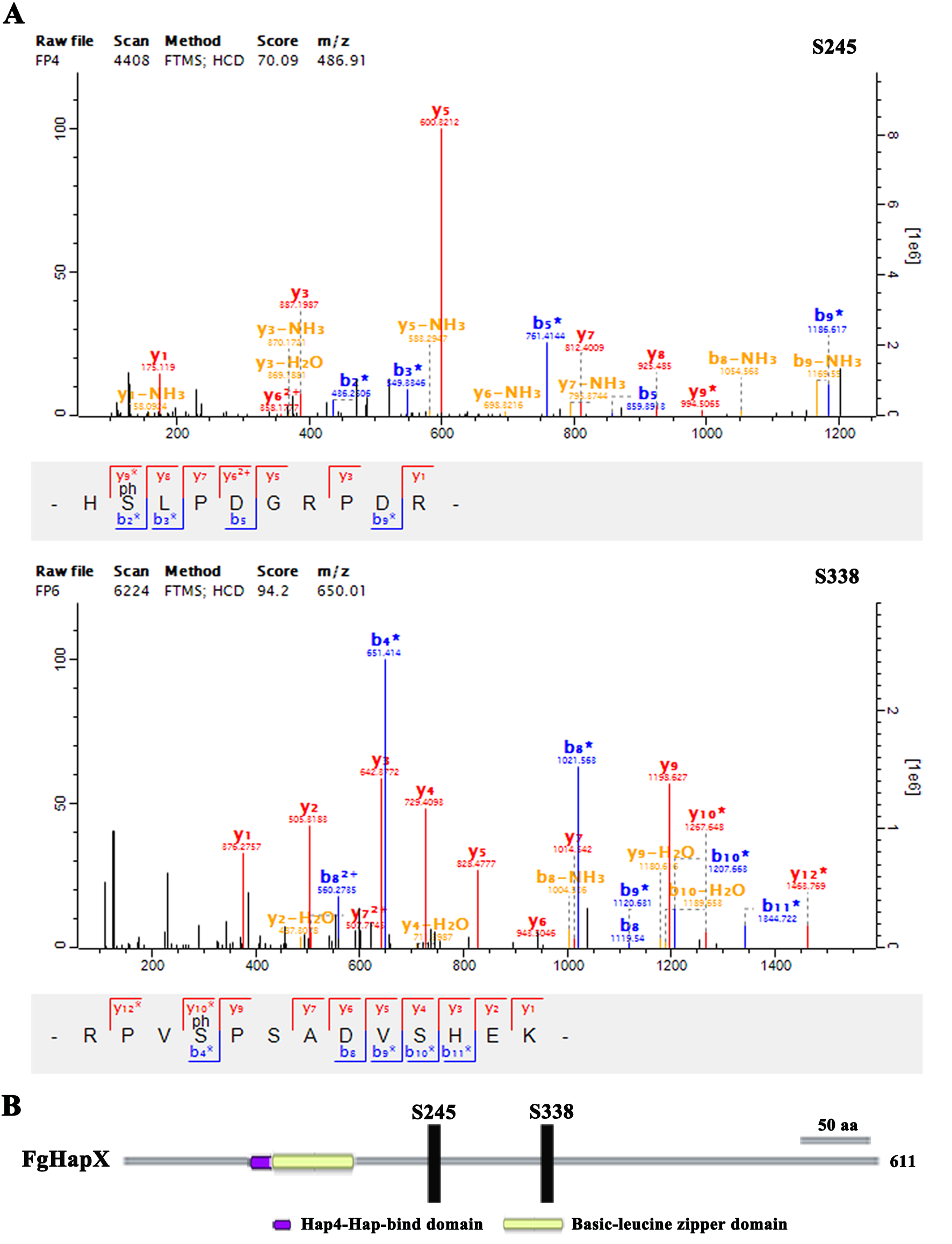

Supplement: S8 Fig — (A) Identification of two serine phosphorylation sites (S245 and S338) in FgHapX. (B) Sketch map of the above two residues in FgHapX. (TIF) [file ppat.1007791.s008.tif]

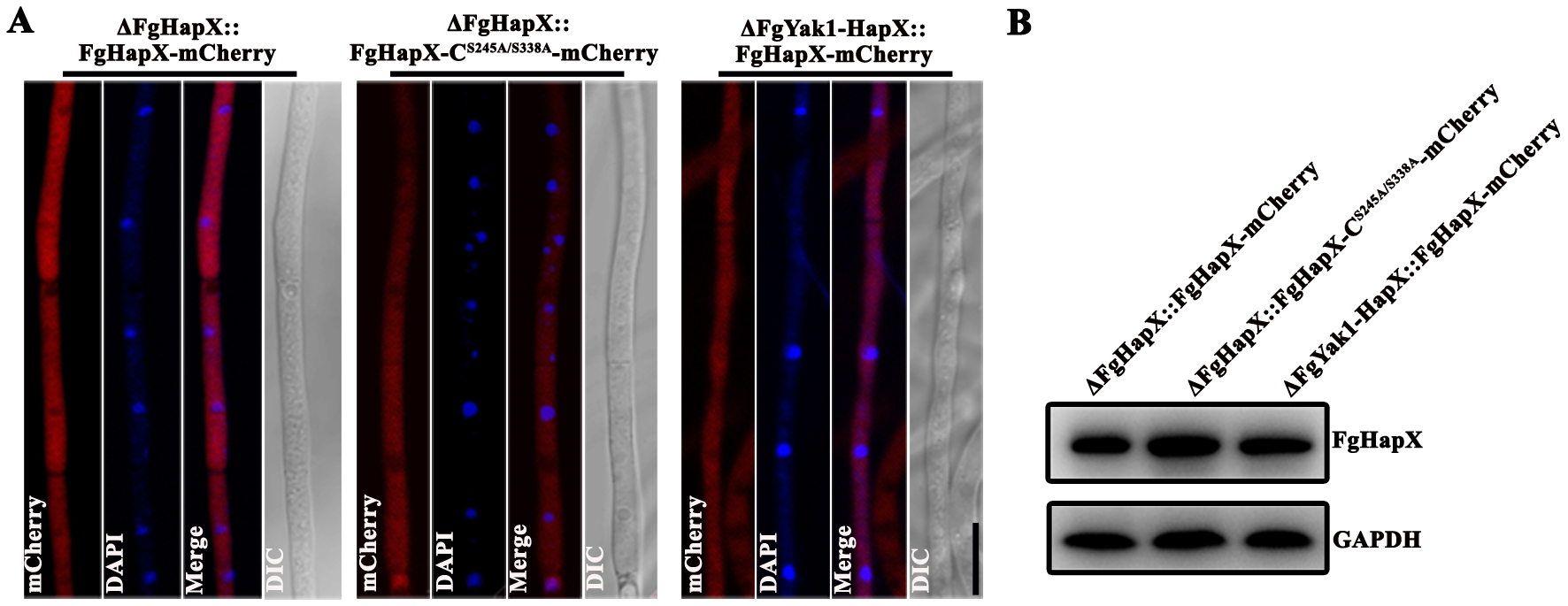

Supplement: S9 Fig — The localization (A) and protein quantity (B) of FgHapX in the wild type, ΔFgHapX-CS245A/S338A mutated in the two phosphorylation sites and ΔFgYak1. Bar = 10 μm. (TIF) [file ppat.1007791.s009.tif]

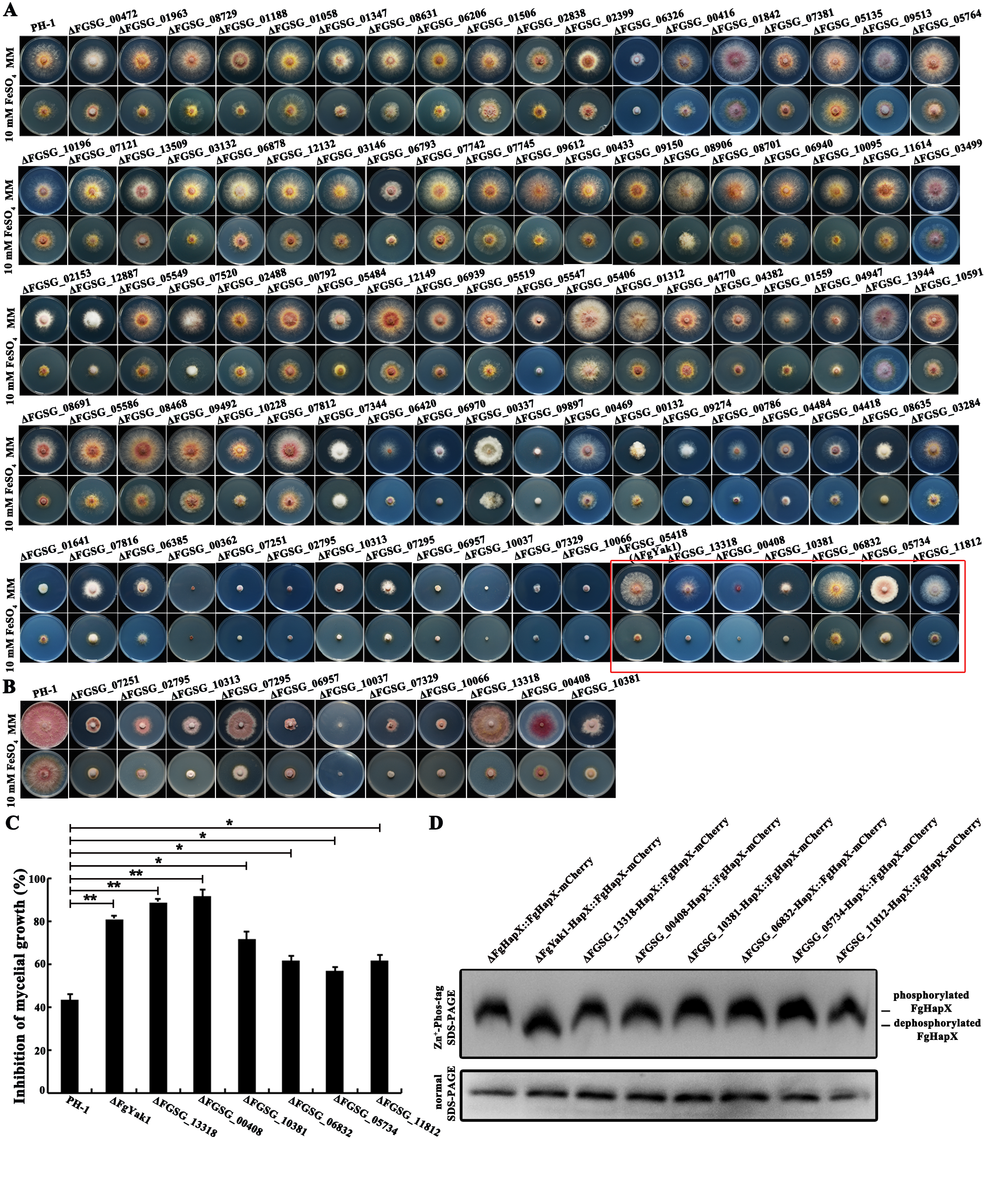

Supplement: S10 Fig — (A and B) A 5-mm mycelial plug of each strain was inoculated on MM without or with 10 mM FeSO4, and then incubated at 25°C for 3 days (A) or 7 days (B). (C) Mycelial growth inhibition of seven kinase mutants was calculated after 3-day-incubation at the conditions (A). Means and standard errors were calculated from three repeats. Significance was measured using an unpaired t-test (*p < 0.05, **p < 0.01). (D) Phos-tag assays for the seven kinase mutants. Protein extracted from each strain was subjected to Phos-tag SDS-PAGE and normal SDS-PAGE followed by immunoblotting with an anti-mCherry antibody. (TIF) [file ppat.1007791.s010.tif]

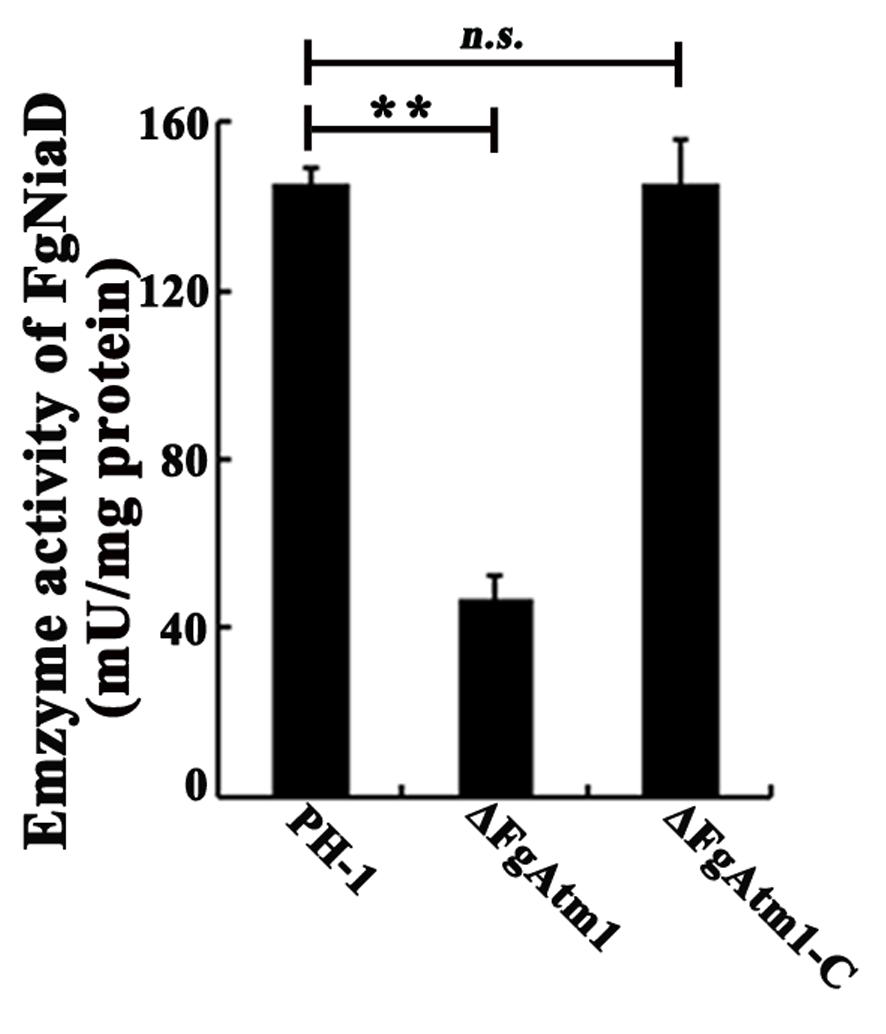

Supplement: S11 Fig — Each strain was cultured in CM at 25°C for 36 hours before activity determination. The FgNiaD activity of the wild type was set as 1. Means and standard errors were calculated from three repeats. Significance was measured using unpaired t-test (n.s. not significant, **p < 0.01). (TIF) [file ppat.1007791.s011.tif]

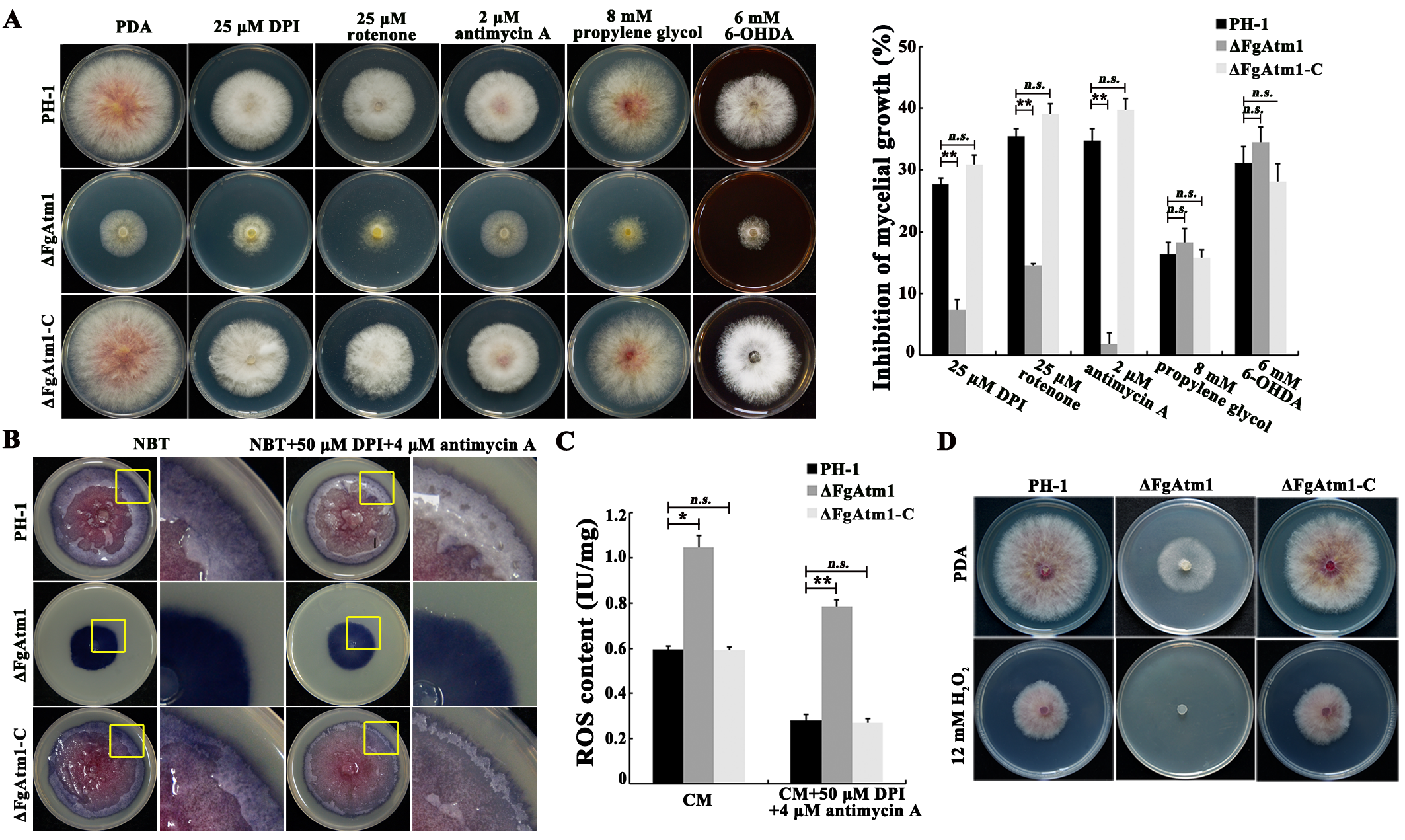

Supplement: S12 Fig — (A) Sensitivity of the wild type and ΔFgAtm1 to different mitochondrial complex inhibitors. A 5-mm mycelial plug of each strain was inoculated on PDA without or with diphenylene iodonium (DPI) and rotenone (complex I), antimycin A (complex III), malonic acid (complex II) or 6-Hydroxydopamine hydrobromide (6-OHDA) (complex IV) at the indicated concentration, and then incubated at 25°C for 3 days. Mycelial growth inhibition of each treatment was calculated after 3-day-incubation. Means and standard errors were calculated from three repeats. Significance was measured using an unpaired t-test (n.s. not significant, **p < 0.01). (B) Reactive oxygen species (ROS) production in the wild type, ΔFgAtm1 and ΔFgAtm1-C. The 3-day-old colony of each strain was stained with 0.05% (wt/vol) ROS indicator nitroblue tetrazolium (NBT) for 2 h with or without 50 μM DPI and 4 μM antimycin A. (C) Quantitative determination of ROS in the wild type, ΔFgAtm1 and ΔFgAtm1-C with or without the treatment of 50 μM DPI and 4 μM antimycin A. Means and standard errors were calculated from three repeats. Significance was measured using an unpaired t-test (n.s. not significant, *p < 0.05, **p < 0.01). (D) Sensitivity of the wild type, ΔFgAtm1 and ΔFgAtm1-C to hydrogen peroxide (H2O2). A 5-mm mycelial plug of each strain was inoculated on PDA amended without or with 12 mM H2O2 and then incubated at 25°C for 3 days. (TIF) [file ppat.1007791.s012.tif]

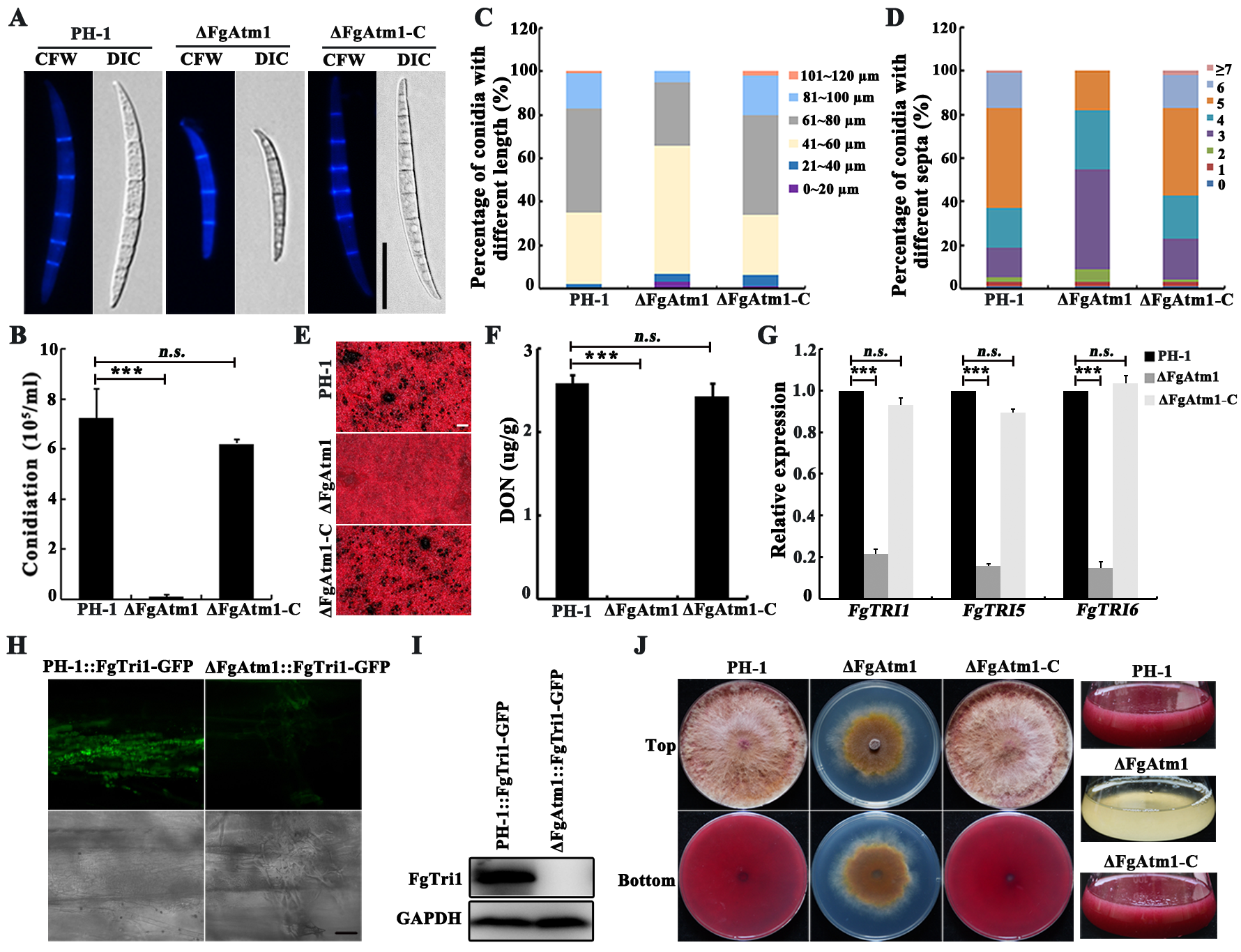

Supplement: S13 Fig — (A) Conidial morphology of the wild type, ΔFgAtm1, and ΔFgAtm1-C. The differential interference contrast (DIC) images of conidia from each strain were captured with an electronic microscope. Bar = 20 μm. Conidia were cultured in CMC liquid medium at 25°C for 4 days in a shaker. (B) Conidia of each strain were quantified after incubation in CMC for 4 days. Means and standard errors were calculated from three repeats. Significance was measured using an unpaired t-test (n.s. not significant, ***p < 0.001). (C and D) Comparisons of conidium length (C) and septum number (D) among the above strains. Conidia were cultured in CMC liquid medium at 25°C for 4 days in a shaker. A total of 200 conidia were examined for each strain. (E) Perithecia of the wild type, ΔFgAtm1 and ΔFgAtm1-C were grown on carrot agar for induction of perithecial formation. Bar = 500 μm. (F) The amount of DON produced by the wild type, ΔFgAtm1 and ΔFgAtm1-C after incubation in trichothecene biosynthesis induction (TBI) medium for 3 days. The dry weight of mycelium is used as an internal reference. Means and standard errors were calculated from three repeats. Significance was measured using an unpaired t-test (n.s. not significant, ***p < 0.001). (G) Relative expression levels of DON biosynthetic genes FgTRI1, FgTRI5, and FgTRI6 in the wild type, ΔFgAtm1, and ΔFgAtm1-C after growth in TBI at 28°C in the dark for 3 days. Means and standard errors were calculated from three repeats. Significance was measured using an unpaired t-test (n.s. not significant, ***p < 0.001). (H) The subcellular localization of FgTri1-GFP. FgTri1-GFP localized to “toxisomes” in PH-1, whereas, the Tri1-GFP labelled toxisomes were nearly undetectable in ΔFgAtm1. Bar = 10 μm. Each strain was examined in artificially wounded wheat seedling leaves 48 h after inoculation. (I) FgTri1-GFP was dramatically reduced in ΔFgAtm1 determined by western blotting with the monoclonal anti-GFP antibody. The protein samples were also detected wit [file ppat.1007791.s013.tif]
