# Supplementary material for: A fungal ABC transporter FgAtm1 regulates iron homeostasis via the transcription factor cascade FgAreA-HapX
Source: PLoS Pathog. 2019 Sep 23;15(9):e1007791. doi: 10.1371/journal.ppat.1007791 (PMC6788720; doi:10.1371/journal.ppat.1007791)
Supplement: S1 Table — (DOCX) [file ppat.1007791.s014.docx]

| **S1 Table.** The primers used in this study. | |
| --- | --- |
| **Oligo Name** | **Sequence** |
| FgAtm1-UP-F^a^ | TTTCCGTCAAATCGACTTGG |
| FgAtm1-UP-R^a^ | CAAAATAGGCATTGATGTGTTGACCTCCTCAACGCGAGCTCATCAT |
| FgAtm1-DOWN-F^b^ | CTCGTCCGAGGGCAAAGGAATAGAGTAGTTGTCCTCAAGGAGGGACAT |
| FgAtm1-DOWN-R^b^ | AAACCATCCTCTCTCTCGAAA |
| FgAtm1-NEST-F^c^ | TATCGGAAGGTTGACTTGAGG |
| FgAtm1-NEST-R^c^ | TTTACCTTGCGGACTCCTG |
| FgAtm1-ID-F^d^ | GGTTGCATCATTTAGCTAGCA |
| FgAtm1-ID-R^d^ | ACCGTCCCTCTATAGCCATAA |
| FgHapX-UP-F | GTGCAATTCCGACTTATGCC |
| FgHapX-UP-R | CAAAATAGGCATTGATGTGTTGACCTCCGATGGGGACGTCGACGGGTA |
| FgHapX-DOWN-F | CTCGTCCGAGGGCAAAGGAATAGAGTAGTGTTCCCCAAACGGACATTG |
| FgHapX-DOWN-R | CATGTACTCAATATCCTTTCC |
| FgHapX-NEST-F | ATTGGAGATCATCAATGATAA |
| FgHapX-NEST-R | CATTATACTTGCTGCTTCAAC |
| FgHapX-ID-F | TCTTCCACCAATCCTTCAGC |
| FgHapX-ID-R | TCCACTGTATCATGTGGACTG |
| FgSreA-UP-F | CCAGTCCATTGACAAAGACGT |
| FgSreA-UP-R | CAAAATAGGCATTGATGTGTTGACCTCCAGGGCTGGACTTGTTGACAA |
| FgSreA-DOWN-F | CTCGTCCGAGGGCAAAGGAATAGAGTAGATGGACTCACCCGAGCCACA |
| FgSreA-DOWN-R | TGAGGTCAAGTGACACCGCT |
| FgSreA-NEST-F | TTGGCTGATCGACTCTGTTG |
| FgSreA-NEST-R | ACGATACAGTGACTCCTTGCT |
| FgSreA-ID-F | TGCATCTTACGGTCTCCGATT |
| FgSreA-ID-R | TGGAGCCTGTGTAGTGACG |
| FgYak1-UP-F | TCCAGTGCTAAACCCTATGG |
| FgYak1-UP-R | CAAAAAATGCTCCTTCAATATCACTAGTACTGGCTGTTGCTGCTATGG |
| FgYak1-DOWN-F | CCTAACCCTAAACCAAAGCATCAGGCCTGATGATACCTTGAATGCGAC |
| FgYak1-DOWN-R | TGAGCATTGGTCTGTTTTGC |
| FgYak1-NEST-F | CGCCCAGCACAATCACTCAT |
| FgYak1-NEST-R | AAGTTGCGAATGAAGTCTCT |
| FgYak1-ID-F | GTCAACGCCCGATAACCACG |
| FgYak1-ID-R | GGCGACCTGTTCAGAGAGCT |
| FgGrx4-UP-F | ACGAAGGTCAGGTTTGATGA |
| FgGrx4-UP-R | CAAAATAGGCATTGATGTGTTGACCTCCTGCCAGGGTTTCGATCTGAT |
| FgGrx4-DOWN-F | CTCGTCCGAGGGCAAAGGAATAGAGTAGTCGGTTGTTGCTGTATGCTG |
| FgGrx4-DOWN-R | CGCCCTTATGGACAAATCC |
| FgGrx4-NEST-F | AAAGGAATTGCTGTGCGGTG |
| FgGrx4-NEST-R | GGCGTCAAGTCTTGTCATTC |
| FgGrx4-ID-F | TTTGCACCAATTGCTGCCCC |
| FgGrx4-ID-R | TCCCTGAGCAATCCCTGCT |
| FgLeu1-UP-F | GCATGGCTGATGAAGTCCTG |
| FgLeu1-UP-R | CAAAATAGGCATTGATGTGTTGACCTCCTTTGACAATGAGACCGCACT |
| FgLeu1-DOWN-F | CTCGTCCGAGGGCAAAGGAATAGAGTAGTCTGGATACAACAAAATGGG |
| FgLeu1-DOWN-R | AGATAGCGCCAACATTTGCC |
| FgLeu1-NEST-F | GTAACTAACAGCCCGGTAGA |
| FgLeu1-NEST-R | TATGTTCTGACCGACCAGTC |
| FgLeu1-ID-F | CGCCTACTACTCTATCACAG |
| FgLeu1-ID-R | ACCGAGATGGACAGAGTCAG |
| FgBio2-UP-F | GCAAACCTCACATAAGCTTG |
| FgBio2-UP-R | CAAAATAGGCATTGATGTGTTGACCTCCTGTGAAAAGGGAAAAGGTAA |
| FgBio2-DOWN-F | CTCGTCCGAGGGCAAAGGAATAGAGTAGACCTGGTTATGATTGTGTAG |
| FgBio2-DOWN-R | GTATGGTATGGTATGCACGC |
| FgBio2-NEST-F | ATGCCGCTCAAATCACGGTA |
| FgBio2-NEST-R | GGAGTGTGTAAAGGATGTGG |
| FgBio2-ID-F | GCTCACTCACAACACATCTC |
| FgBio2-ID-R | GTGATGTTGCTTCAGACGTC |
| G418-probe-F^e^ | AAGATGGATTGCACGCAGGTT |
| G418-probe-R^e^ | AAGAAGGCGATAGAAGGCGAT |
| FgAtm1-probe-F | TCTATCCCACCAACCTTCTT |
| FgAtm1-probe-R | TTGGGAAGGAATATCTGGCT |
| FgAtm1-GFP-F^f^ | ACTCACTATAGGGCGAATTGGGTACTCAAATTGGTTACGAACAGGCAAGATAGTTC |
| FgAtm1-GFP-R^f^ | CACCACCCCGGTGAACAGCTCCTCGCCCTTGCTCACGTTCTTCTTCTGTTCGTCAT |
| FgAtm1-GFP-ID-F | TTGTCCTCAAGGAGGGACAT |
| FgAtm1^N1-111^-GFP-F | ACTCACTATAGGGCGAATTGGGTACTCAAATTGGTTACGAACAGGCAAGATAGTTC |
| FgAtm1^N1-111^-GFP-R | CACCACCCCGGTGAACAGCTCCTCGCCCTTGCTCACCTTGGTACCCCAGTCATCC |
| FgYak1-GFP-F | ACTCACTATAGGGCGAATTGGGTACTCAAATTGGTTCGAGTTCCAAGGTAATGCG |
| FgYak1-GFP-R | CACCACCCCGGTGAACAGCTCCTCGCCCTTGCTCACATGCCAGGTGTTTTGATTT |
| FgYak1-GFP-ID-F | AGCAGCAACAGAGCGGCATC |
| FgTri1-GFP-F | ACTCACTATAGGGCGAATTGGGTACTCAAATTGGTTCTTCTCGCTCGTAGTAACAG |
| FgTri1-GFP-R | CACCACCCCGGTGAACAGCTCCTCGCCCTTGCTCACGTCATCCTGTACCAATTCCA |
| FgTri1-GFP-ID-F | CTACGAGAACCCTGAAAAGT |
| FgVac8-GFP-F | ACTCACTATAGGGCGAATTGGGTACTCAAATTGGTTATGCTGTATGTCTCAGGATC |
| FgVac8-GFP-R | CACCACCCCGGTGAACAGCTCCTCGCCCTTGCTCACGCCCTCGATATGGGCCTTGG |
| FgVac8-GFP-ID-F | AGCACATTGCAGTCTGGACAC |
| GFP-ID-R | GACACGCTGAACTTGTGGCCGTT |
| FgHapX-mCherry-F^g^ | GACGCAGTTAGCAGCAGCAA |
| FgHapX-mCherry-R^g^ | GGCCATGTTATCCTCCTCGCCCTTGCTCACAATTCCTCTCCCAAACCGAA |
| FgLeu1-mCherry-F | GTGTACGATCATCGAGTACAG |
| FgLeu1-mCherry-R | GGCCATGTTATCCTCCTCGCCCTTGCTCACCCACTCGAGAGGTTCCTTCT |
| mCherry-F | GTGAGCAAGGGCGAGGAGGAT |
| mCherry-R | TTATCACTTGTACAGCTCGTCC |
| neo-mCherry-F | GGCGGCATGGACGAGCTGTACAAGTGATAAGGAGGTCAACACATCAATGCT |
| neo-mCherry-R | AACCCAGGGGCTGGTGACGGA |
| FgGrx4-Flag-F^h^ | CTATAGGGCGAATTGGGTACTCAAATTGGTTACGAAGGTCAGGTTTGATGA |
| FgGrx4-Flag-R^h^ | CTTTATAATCACCGTCATGGTCTTTGTAGTCAGGCGCTGCAGGAGCGCTCA |
| FgGrx4-Flag-ID-F | CTTGTTGGCGGTTTGGATAT |
| FgYak1-Flag-F | AAGTGTGTCTTGATGCTCGAGCGAGTTCCAAGGTAATGCGAA |
| FgYak1-Flag-R | ATGGTCTTTGTAGTCCTCGAGATGCCAGGTGTTTTGATTTCCC |
| FgYak1-Flag-ID-F | AGCAGCAACAGAGCGGCATC |
| FgHapX-BD-F^i^ | ATGGCCATGGAGGCCGAATTCATGTCGGCATCTTCTCCTCAAC |
| FgHapX-BD-R^i^ | TCGACGGATCCCCGGGAATTCTTAAATTCCTCTCCCAAACCGA |
| FgGrx4-AD-F^i^ | GCCATGGAGGCCAGTGAATTCATGCCGACCATTACAGAAATCAC |
| FgGrx4-AD-R^i^ | ATGCCCACCCGGGTGGAATTCTCAAGGCGCTGCAGGAGC |
| FgGrx4-TRX-AD-F | GCCATGGAGGCCAGTGAATTCATGCCGACCATTACAGAAATCAC |
| FgGrx4-TRX-AD-R | ATGCCCACCCGGGTGGAATTCTCAAAGCTCCTCCTTCTTCTTCTC |
| FgGrx4-GRX-AD-F | GCCATGGAGGCCAGTGAATTCCAGTCAGGTGAAAACGCCGC |
| FgGrx4-GRX-AD-R | ATGCCCACCCGGGTGGAATTCTCAAGGCGCTGCAGGAGC |
| FgHapX-68-F^j^ | TTTCGTAGGAACCCAATCTTCAAAATGTCGGCATCTTCTCCTCA |
| FgHapX-68-R^j^ | GTTCGGGATCTTGCAGGCCGGGCGAATTCCTCTCCCAAACCGAA |
| FgHapX-68-ID-F^j^ | TCTCATGGCCGCCAACTTTA |
| FgGrx4-65-F^j^ | TTTCGTAGGAACCCAATCTTCAAAATGCCGACCATTACAGAAAT |
| FgGrx4-65-R^j^ | GCTCACCATCGTGGCGATGGAGCGAGGCGCTGCAGGAGCGCTCA |
| FgGrx4-65-ID-F^j^ | CTTGTTGGCGGTTTGGATAT |
| FgHapX-RT-F^k^ | ACTCTTCTGTCGTCTCATGG |
| FgHapX-RT-R^k^ | AGGCATCATTGAACCAGGAG |
| FgSidA-RT-F | TGCTGTTATTGGTGCCGGTC |
| FgSidA-RT-R | AGCCTCGGTAAGCAGTGTCT |
| FgMirB-RT-F | CACAAGGCGAAGAAGAATAT |
| FgMirB-RT-R | AGGACGAAACCAACTACAAT |
| FgSidC-RT-F | CAACAGCGGATGCGATTCTC |
| FgSidC-RT-R | TCTCAAACACGCTCACACTC |
| FgSidD-RT-F | TCACCATCATTTTCATCTCC |
| FgSidD-RT-R | ACTTTCTCTATGTCTTGCTC |
| FgSidF1-RT-F | TCACGAATGGCAAAACGATC |
| FgSidF1-RT-R | AAGCGTACATCACCCACAAG |
| FgSidG-RT-F | GTTTTGGGGTCAGGGTTACG |
| FgSidG-RT-R | CCACTTCACCCTCCTCAATG |
| FgAcoA-RT-F | TCACTGTCAAGGGTGGCACT |
| FgAcoA-RT-R | GTAAGAGCGGGCGAAGTCAC |
| FgCycA-RT-F | GTTACTCTTACACCGATGCC |
| FgCycA-RT-R | ATTTGGTGGAGTCCTTGAGG |
| FgHemA-RT-F | AGATTCACACTTCCAGCAAC |
| FgHemA-RT-R | GCTCCGTGTTGTAAAAGGTT |
| FgLysF-RT-F | GAGAAGGTTGAGGGCGAGAT |
| FgLysF-RT-R | CTGTGTCGTAGTTCTCCATG |
| FgHapX-RT-F | ACTCTTCTGTCGTCTCATGG |
| FgHapX-RT-R | AGGCATCATTGAACCAGGAG |
| FgSreA-RT-F | AGAACCAGTTCCTCTCGATG |
| FgSreA-RT-R | TTGATCCCCTCTCTGCATT |
| FgTri1-RT-F | CAGGAGAGTTATTGCCGAAG |
| FgTri1-RT-R | ACTTTTCAGGGTTCTCGTAG |
| FgTri6-RT-F | AAATGCCCATTCCCTAGTTG |
| FgTri6-RT-F | ATCTCGCATGTTATCCACCCT |
| FgActin-RT-F | ATCCACGTCACCACTTTCAA |
| FgActin-RT-R | TGCTTGGAGATCCACTTTG |
| FgHapX-Mu-P1^l^ | GTGTCTTGGTCCTTGACTTG |
| FgHapX-Mu-P2 | CAAGTCAAGGACCAAGACACGCTCTTCCCGACGGACGACC |
| FgHapX-Mu-P3 | GACGGGACGCTTGAGTTCCT |
| FgHapX-Mu-P4 | AGGAACTCAAGCGTCCCGTCGCACCGTCAGCAGATGTATC |
| FgHapX-ChIP-F^m^ | TCGGTCGTAATGTGGTAAAC |
| FgHapX-ChIP-R^m^ | AAAGAAGGGAAATGAAGCGC |
| FgSreA-ChIP-F | TTTTTCGTCTCATTCTGGGC |
| FgSreA-ChIP-R | CGTAAGTGTTGCTGTAGGCT |
| FgSidA-ChIP-F | CTCTCTTCTGCCGTTGTGAT |
| FgSidA-ChIP-R | AAGACCGCAACTAAACCCAC |
| FgLYSF-ChIP-F | GTAGGTGGATGCTCAGAAAC |
| FgLYSF-ChIP-R | TACTACACAACTTCCCCTCC |
| FgActin-ChIP-F | ATCCACGTCACCACTTTCAA |
| FgActin-ChIP-R | TGCTTGGAGATCCACTTTG |
| FgAcoA-EMSA-F^n^ | GTGAGATAACTCGTAACTACG |
| FgAcoA-EMSA-R^n^ | GGTAAGCTAACTGACAAGTCG |
| FgCycA-EMSA-F | CTGAGAAAGTGGGATGCTGAA |
| FgCycA-EMSA-R | ATGACAGCATGACACAACCG |
| FgHemA-EMSA-F | CGTTCAATGTGATAACAGAGC |
| FgHemA-EMSA-R | CGCAGTCTTCAGTTCCAAAG |
| FgLysF-EMSA-F | GTAGGTGGATGCTCAGAAAC |
| FgLysF-EMSA-R | CTTCTTTCCTTGAAGCCGG |
| FgSreA-EMSA-F | TGCCTACTGTACGCCTACCT |
| FgSreA-EMSA-R | AGGGCTGGACTTGTTGACAA |
| HAPX-pGEX4T-F^o^ | tccccgaattcccgggtcgacATGTCGGCATCTTCTCCTCAAC |
| HAPX-pGEX4T-R^o^ | gatgcggccgctcgagtcgacAGAGTGCTGAGCGCTCATAGTTC |
|  |  |
|  |  |
|  |  |
| ^a^"UP" represent PCR primers to amplify upstream fragment for the construction of gene deletion mutants. | |
| ^b^"DOWN" represent PCR primers to amplify downstream fragment for the construction of gene deletion mutants. | |
| ^c^"NEST" represent PCR primers to amplify upstream-HPH-downstream fragment for the construction of gene deletion mutants. | |
| ^d^"ID" represent PCR primers for identification of gene deletion transformants. | |
| ^e^"probe" represent PCR primers to amplify probe for Southern analysis. | |
| ^f^"GFP" represent PCR primers used for the construction of GFP-fusion cassettes. | |
| ^g^"mCherry" represent primers used for the construction of mCherry-fusion cassettes. | |
| ^h^"Flag" represent PCR primers used for the construction of Flag-fusion cassettes. | |
| ^i^"BD/AD" represent PCR primers used for the construction of BD/AD-fusion cassettes in yeast two-hybrid (Y2H) assays. | |
| j"68/65" represent PCR primers used for the construction of CYFP/NYFP-fusion cassettes in bimolecular fluorescence complementation (BiFC) assay. | |
| ^k^"RT" represent PCR primers used for quantitative reverse transcription PCR (qRT-PCR). | |
| ^l^"Mu" represent PCR primers to amplify *FgHAPX* fragment carrying point mutations. | |
| ^m^"ChIP" represent PCR primers uesd for Chromatin immunoprecipitation (ChIP)-qPCR. | |
| ^n^"ChIP" represent PCR primers uesd for electrophoretic mobility shift assay (EMSA). | |
| ^o^"pGEX4T" represent PCR primers used for the construction of GST-fusion cassettes. | |
