# Supplementary material for: A fungal ABC transporter FgAtm1 regulates iron homeostasis via the transcription factor cascade FgAreA-HapX
Source: PLoS Pathog. 2019 Sep 23;15(9):e1007791. doi: 10.1371/journal.ppat.1007791 (PMC6788720; doi:10.1371/journal.ppat.1007791)
Supplement: S2 Table — (DOCX) [file ppat.1007791.s015.docx]

| **S2 Table.** Serial analysis of gene expression (SAGE) for identification of differential expression (>2-folds) genes in *FgATM1* deletion mutant as compared with those in the wild type. | | | | | | | | | | | |
| --- | --- | --- | --- | --- | --- | --- | --- | --- | --- | --- | --- |
| **gene id** | **gene name** | **description** | **GO_id** | **GO_term** | **locus** | **G2** | **G1** | **log2FC** | **Pvalue** | **Qvalue** | **updown** |
|  |  |  |  |  |  |  |  |  |  |  |  |
| gene10030 | FGSG_11030 | hypothetical protein | GO:0000293  GO:0010106  GO:0006879 | ferric-chelate reductase activity  cellular response to iron ion starvation  cellular iron ion homeostasis | NC_026476.1:5912120-5914021 | 24.45848 | 4.66377 | 2.390766 | 3.37E-11 | 3.75E-10 | UP |
| gene5243 | FGSG_08121 | hypothetical protein | GO:0000293  GO:0010106  GO:0006879 | ferric-chelate reductase activity | NC_026475.1:2845566-2849155 | 56.25121 | 8.31632 | 2.757867 | 1.32E-17 | 2.84E-16 | UP |
|  |  |  |  | cellular response to iron ion starvation |  |  |  |  |  |  |  |
|  |  |  |  | cellular iron ion homeostasis |  |  |  |  |  |  |  |
| gene2107 | FGSG_01789 | hypothetical protein | GO:0000293  GO:0015891  GO:0006826 | ferric-chelate reductase activity | NC_026474.1:5877924-5880194 | 290.7883 | 0.116109 | 11.29027 | 2.63E-92 | 6.51E-89 | UP |
|  |  |  |  | siderophore transport |  |  |  |  |  |  |  |
|  |  |  |  | iron ion transport |  |  |  |  |  |  |  |
| gene7173 | FGSG_12485 | hypothetical protein | GO:0000293  GO:0015891  GO:0006826 | ferric-chelate reductase activity | NC_026475.1:7517092-7519272 | 0.937705 | 0 | Inf | 5.47E-05 | 0.000263 | UP |
|  |  |  |  | siderophore transport |  |  |  |  |  |  |  |
|  |  |  |  | iron ion transport |  |  |  |  |  |  |  |
| gene7871 | FGSG_04780 | hypothetical protein | GO:0000293  GO:0015891  GO:0006826 | ferric-chelate reductase activity  siderophore transport  iron ion transport | NC_026476.1:268810-270960 | 6.383916 | 277.3967 | -5.44137 | 2.1E-42 | 3.02E-40 | DOWN |
| gene9922 | FGSG_11119 | hypothetical protein | GO:0000293  GO:0015891  GO:0006826  GO:0006879 | ferric-chelate reductase activity | NC_026476.1:5680661-5683683 | 0.058052 | 3.326496 | -5.84051 | 1.39E-16 | 2.78E-15 | DOWN |
|  |  |  |  | siderophore transport |  |  |  |  |  |  |  |
|  |  |  |  | iron ion transport |  |  |  |  |  |  |  |
|  |  |  |  | cellular iron ion homeostasis |  |  |  |  |  |  |  |
| gene5071 | FGSG_13404 | hypothetical protein | GO:0000293 | ferric-chelate reductase activity | NC_026475.1:2415348-2417259 | 108.3758 | 299.972 | -1.46879 | 1.85E-07 | 1.32E-06 | DOWN |
| gene9351 | FGSG_06060 | hypothetical protein | GO:0000293 | ferric-chelate reductase activity | NC_026476.1:4175904-4177865 | 52.29504 | 510.8286 | -3.28809 | 2.48E-24 | 9.49E-23 | DOWN |
|  |  |  | GO:0015891 | siderophore transport |  |  |  |  |  |  |  |
| gene4702 | FGSG_08579 | hypothetical protein | GO:0000293 | ferric-chelate reductase activity | NC_026475.1:1382724-1385321 | 38.02935 | 239.6896 | -2.65598 | 3.76E-16 | 7.19E-15 | DOWN |
|  |  |  | GO:0006826 | iron ion transport |  |  |  |  |  |  |  |
| gene6530 | FGSG_03746 | hypothetical protein | GO:0010106 | cellular response to iron ion starvation | NC_026475.1:6088484-6089535 | 333.4307 | 10.14601 | 5.038403 | 4.4E-39 | 5.09E-37 | UP |
| gene2511 | FGSG_02143 | plasma membrane iron permease | GO:0010106 | cellular response to iron ion starvation | NC_026474.1:7009636-7010794 | 77.94785 | 15.34511 | 2.344731 | 2.08E-13 | 2.99E-12 | UP |
| gene4538 | FGSG_08721 | superoxide dismutase | GO:0010106 | cellular response to iron ion starvation | NC_026475.1:869730-871092 | 383.648 | 95.01321 | 2.013583 | 3.22E-08 | 2.55E-07 | UP |
| gene10034 | FGSG_11026 (FgSidC) | nonribosomal peptide synthetase | GO:0010106 | cellular response to iron ion starvation | NC_026476.1:5925029-5939403 | 2.866441 | 0.008992 | 8.316403 | 4.18E-36 | 4.11E-34 | UP |
| gene8316 | FGSG_05160 | hypothetical protein | GO:0010106 | cellular response to iron ion starvation | NC_026476.1:1344183-1345447 | 560.0261 | 53.33181 | 3.392426 | 2.21E-23 | 7.66E-22 | UP |
| gene6529 | FGSG_03747 (FgSidD) | nonribosomal peptide synthetase | GO:0010106 | cellular response to iron ion starvation | NC_026475.1:6080983-6087254 | 162.9593 | 11.97915 | 3.765915 | 4.55E-23 | 1.53E-21 | UP |
| gene2510 | FGSG_02142 | hypothetical protein | GO:0010106 | cellular response to iron ion starvation | NC_026474.1:7007272-7009207 | 115.755 | 6.256824 | 4.2095 | 1.68E-34 | 1.42E-32 | UP |
| gene8314 | FGSG_05159 | iron transport multicopper oxidase FET3 precursor | GO:0010106 | cellular response to iron ion starvation | NC_026476.1:1339324-1341541 | 615.9172 | 98.3367 | 2.646935 | 1.02E-11 | 1.22E-10 | UP |
| gene665 | FGSG_00540 (FgSidG) | gnat family acetyltransferase | GO:0008080 | N-acetyltransferase activity | NC_026474.1:1696600-1697304 | 0.33663 | 0 | Inf | 0.143162 | 0.26002 | UP |
| gene10704 | FGSG_11275 (FgMirB) | siderophore iron transporter mirb | GO:0055085  [GO:0016021](http://amigo.geneontology.org/amigo/term/GO:0016021) | transmembrane transport  integral component of membrane | NC_026476.1:7584938-7586825 | 569.0369 | 51.17974 | 3.474877 | 2E-23 | 7E-22 | UP |
| gene8552 | FGSG_05371 (FgSidA) | l-ornithine n5-oxygenase | [GO:0016491](http://www.ebi.ac.uk/ego/GTerm?id=GO:0016491)  [GO:0055114](http://www.ebi.ac.uk/ego/GTerm?id=GO:0055114) | oxidoreductase activity  oxidation-reduction process | NC_026476.1:2033838-2036149 | 1342.666 | 36.34423 | 5.20723 | 1.23E-32 | 9.1E-31 | UP |
| gene5838 | FGSG_04334 (FgSidF) | hypothetical protein | - | - | NC_026475.1:4365867-4367440 | 658.0231 | 18.96592 | 5.116657 | 1.23E-33 | 9.73E-32 | UP |
| gene6531 | FGSG_03745 (FgSidF) | hypothetical protein | - | - | NC_026475.1:6091653-6093266 | 1236.334 | 57.90664 | 4.416196 | 3.13E-31 | 2.1E-29 | UP |
| gene786 | FGSG_00646 | hypothetical protein | GO:0006879 | cellular iron ion homeostasis | NC_026474.1:2067007-2068348 | 485.4174 | 148.3814 | 1.709916 | 2.47E-06 | 1.49E-05 | UP |
| gene9115 | FGSG_05848 | hypothetical protein | GO:0006879 | cellular iron ion homeostasis | NC_026476.1:3550617-3552604 | 218.0598 | 16.33497 | 3.738688 | 8.15E-24 | 2.95E-22 | UP |
| gene2641 | FGSG_02252 | hypothetical protein | GO:0006879 | cellular iron ion homeostasis | NC_026474.1:7310573-7312333 | 158.7044 | 17.29528 | 3.197892 | 1.13E-21 | 3.41E-20 | UP |
| gene10170 | FGSG_10911 | iron-sulfur clusters transporter ATM1 | GO:0006879 | cellular iron ion homeostasis | NC_026476.1:6282831-6284977 | 11.84765 | 46.41799 | -1.97008 | 1.12E-12 | 1.5E-11 | DOWN |
| gene7871 | FGSG_04780 | hypothetical protein | GO:0006879 | cellular iron ion homeostasis | NC_026476.1:268810-270960 | 6.383916 | 277.3967 | -5.44137 | 2.1E-42 | 3.02E-40 | DOWN |
| gene67 | FGSG_11658 | hypothetical protein | GO:0006879 | cellular iron ion homeostasis | NC_026474.1:171341-172428 | 0.177128 | 13.39014 | -6.24024 | 6.22E-20 | 1.64E-18 | DOWN |
| gene4910 | FGSG_08403 | hypothetical protein | GO:0006879 | cellular iron ion homeostasis | NC_026475.1:1996911-2000088 | 77.39496 | 243.1555 | -1.65157 | 4.93E-06 | 2.85E-05 | DOWN |
| gene10203 | FGSG_10887 | hypothetical protein | GO:0006879 | cellular iron ion homeostasis | NC_026476.1:6384898-6386048 | 52.08368 | 206.3131 | -1.98593 | 5.23E-12 | 6.48E-11 | DOWN |
| gene12239 | FGSG_07679 | hypothetical protein | GO:0006879 | cellular iron ion homeostasis | NC_026477.1:4176257-4177194 | 13.40851 | 447.7496 | -5.06147 | 6.03E-44 | 9.96E-42 | DOWN |
| gene1522 | FGSG_01284 | hypothetical protein | GO:0005506  GO:0020037 | iron ion binding  heme binding | NC_026474.1:4234697-4236410 | 7.587272 | 0.719544 | 3.398427 | 2.1E-10 | 2.16E-09 | UP |
| gene2506 | FGSG_02138 | hypothetical protein | GO:0005506  GO:0020037 | iron ion binding  heme binding | NC_026474.1:6996999-6998704 | 13.79443 | 2.373076 | 2.539256 | 1.52E-09 | 1.42E-08 | UP |
| gene7418 | FGSG_12534 | hypothetical protein | GO:0005506  GO:0020037 | iron ion binding  heme binding | NC_026475.1:8076410-8078098 | 17.19308 | 212.6892 | -3.62885 | 3.65E-12 | 4.58E-11 | DOWN |
| gene2082 | FGSG_01767 | hypothetical protein | GO:0005506  GO:0020037 | iron ion binding  heme binding | NC_026474.1:5819241-5821146 | 12.4299 | 369.8278 | -4.89497 | 2.33E-41 | 3.17E-39 | DOWN |
| gene2929 | FGSG_02502 | C-5 sterol desaturase | GO:0005506 | iron ion binding | NC_026474.1:8049520-8051065 | 102.2698 | 409.2174 | -2.00049 | 0.000959 | 0.003561 | DOWN |
| gene2905 | FGSG_02482 | sulfite reductase subunit beta | GO:0020037 | heme binding | NC_026474.1:7980996-7986357 | 14.43545 | 59.93283 | -2.05373 | 2.56E-12 | 3.26E-11 | DOWN |
| gene5289 | FGSG_08079 | hypothetical protein | GO:0005506  GO:0020037 | iron ion binding  heme binding | NC_026475.1:2957359-2959105 | 0.114712 | 10.22108 | -6.47739 | 1.13E-24 | 4.49E-23 | DOWN |
| gene5282 | FGSG_13445 | benzoate 4-monooxygenase | GO:0005506  GO:0020037 | iron ion binding  heme binding | NC_026475.1:2942328-2943999 | 0.041373 | 0.585708 | -3.82341 | 0.002434 | 0.00821 | DOWN |
| gene1205 | FGSG_01000 | cytochrome P450 51 | GO:0005506  GO:0020037 | iron ion binding  heme binding | NC_026474.1:3291914-3293662 | 26.01158 | 77.89584 | -1.58239 | 1.28E-05 | 6.92E-05 | DOWN |
| gene10209 | FGSG_10881 (FgCycA) | cytochrome c | GO:0005506  GO:0020037 | iron ion binding  heme binding | NC_026476.1:6400293-6401506 | 653.7187 | 2183.515 | -1.73991 | 8.3E-08 | 6.21E-07 | DOWN |
| gene307 | FGSG_11720 | hypothetical protein | GO:0051539  GO:0051539 | 4 iron, 4 sulfur cluster binding  4 iron, 4 sulfur cluster binding | NC_026474.1:734949-736704 | 3.446528 | 1.003824 | 1.779638 | 0.001296 | 0.004665 | UP |
| gene3067 | FGSG_02624 | iron sulfur cluster assembly protein 1 | GO:0006879 | cellular iron ion homeostasis | NC_026474.1:8442991-8444155 | 369.7346 | 72.70528 | 2.346358 | 1.74E-11 | 2.02E-10 | UP |
| gene13316 | FGSG_09250 | NADH dehydrogenase flavoprotein 1 | GO:0051539  GO:0051539 | 4 iron, 4 sulfur cluster binding  4 iron, 4 sulfur cluster binding | NC_026477.1:7098911-7100704 | 163.8448 | 435.6589 | -1.41087 | 1.59E-06 | 9.87E-06 | DOWN |
| gene3652 | FGSG_10198 | aconitate hydratase | GO:0051539 | 4 iron, 4 sulfur cluster binding | NC_026474.1:10119936-10122517 | 15.35145 | 39.40992 | -1.36018 | 1.56E-07 | 1.12E-06 | DOWN |
| gene10124 | FGSG_10949 (FgLysF) | homoaconitase | GO:0020037 | heme binding | NC_026476.1:6171622-6174294 | 10.64799 | 28.79181 | -1.43508 | 3.03E-08 | 2.42E-07 | DOWN |
| gene7458 | FGSG_02974 | peroxidase/catalase 2 | GO:0020037 | heme binding | NC_026475.1:8164762-8167355 | 17.8565 | 346.3656 | -4.27777 | 4.59E-38 | 4.95E-36 | DOWN |
| gene13513 | FGSG_09086 | hypothetical protein | GO:0020037 | heme binding | NC_026477.1:7551210-7552989 | 5.258028 | 29.00679 | -2.4638 | 6.49E-05 | 0.000309 | DOWN |
| gene4653 | FGSG_08620 | hypothetical protein | GO:0020037 | heme binding | NC_026475.1:1237381-1238618 | 151.148 | 497.4743 | -1.71866 | 1.08E-07 | 7.9E-07 | DOWN |
| gene901 | FGSG_00743 | hypothetical protein | GO:0020037 | heme binding | NC_026474.1:2436714-2437724 | 189.8773 | 576.6073 | -1.60252 | 1.6E-08 | 1.33E-07 | DOWN |
| gene3606 | FGSG_10160 | hypothetical protein | [GO:0033014](http://amigo.geneontology.org/amigo/term/GO:0033014) | tetrapyrrole biosynthetic process | NC_026474.1:9983724-9984455 | 34.46596 | 128.0678 | -1.89366 | 1.68E-11 | 1.95E-10 | DOWN |
| gene11754 | FGSG_07266 (FgHemA) | 5-aminolevulinate synthase | [GO:0003870](http://amigo.geneontology.org/amigo/term/GO:0003870) | 5-aminolevulinate synthase activity | NC_026477.1:2949404-2951693 | 90.74537 | 119.2107 | -0.39362 | 0.056003 | 0.122592 | DOWN |
| gene12579 | FGSG_07953 (FgAcoA) | aconitate hydratase | [GO:0030170](http://amigo.geneontology.org/amigo/term/GO:0030170)  [GO:0051539](http://amigo.geneontology.org/amigo/term/GO:0051539)  [GO:0003994](http://amigo.geneontology.org/amigo/term/GO:0003994) | pyridoxal phosphate binding  4 iron, 4 sulfur cluster binding | NC_026477.1:4993771-4996644 | 198.1563 | 376.384 | -0.92557 | 0.002226 | 0.007567 | DOWN |
|  |  |  |  | aconitate hydratase activity |  |  |  |  |  |  |  |
| gene9206 | FGSG_05930 (FgHapX) | hypothetical protein | [GO:0006099](http://amigo.geneontology.org/amigo/term/GO:0006099)  GO:0010106  GO:0006879 | tricarboxylic acid cycle  cellular response to iron ion starvation | NC_026476.1:3798671-3800888 | 168.038 | 27.22425 | 2.625824 | 1.37E-15 | 2.47E-14 | UP |
|  |  |  |  | cellular iron ion homeostasis |  |  |  |  |  |  |  |
| gene12933 | FGSG_09565 (FgSreA) | hypothetical protein | GO:0003700 | transcription factor activity, sequence-specific DNA binding | NC_026477.1:6029811-6031588 | 0 | 16.73886 | #NAME? | 9.12E-39 | 1.04E-36 | DOWN |
| gene4637 | FGSG_08634 (FgAreA) | nitrogen regulatory protein areA |  |  | NC_026475.1:1188524-1191660 | 51.62151 | 30.43204 | 0.762381 | 0.043865 | 0.099853 | UP |
