# Supplementary material for: A fungal ABC transporter FgAtm1 regulates iron homeostasis via the transcription factor cascade FgAreA-HapX
Source: PLoS Pathog. 2019 Sep 23;15(9):e1007791. doi: 10.1371/journal.ppat.1007791 (PMC6788720; doi:10.1371/journal.ppat.1007791)
Supplement: S3 Table — (DOCX) [file ppat.1007791.s016.docx]

| **S3 Table. A list of putative FgHapX-interacting proteins identified by the yeast two-hybrid assay.** | | |
| --- | --- | --- |
| **Proteins** | **Homologs in *S. cerevisiae*** | **Putative functions** |
| FGSG_06257 | TDH3 YGR192C | Glyceraldehyde-3-phosphate dehydrogenase (GAPDH), isozyme 3; involved in glycolysis and gluconeogenesis; tetramer that catalyzes the reaction of glyceraldehyde-3-phosphate to 1,3 bis-phosphoglycerate; detected in the cytoplasm and cell wall; GAPDH-derived antimicrobial peptides secreted by S. cerevisiae are active against a wide variety of wine-related yeasts and bateria; binds AU-rich RNA; TDH3 has a paralog, TDH2, that arose from the whole genome duplication |
| FGSG_07129 | ERS1 / YCR075C | Protein involved in cystine transport; localizes to the vacuole, plasma membrane and endosome; similarity to human cystinosin, a H(+)-driven transporter involved in L-cystine export from lysosomes and implicated in the disease cystinosis; contains seven transmembrane domains; mutation is functionally complemented by human CTNS |
| FGSG_08122 | * |  |
| FGSG_00300 | ATP5 / YDR298C | Subunit 5 of the stator stalk of mitochondrial F1F0 ATP synthase; F1F0 ATP synthase is a large, evolutionarily conserved enzyme complex required for ATP synthesis; homologous to bovine subunit OSCP (oligomycin sensitivity-conferring protein); phosphorylated |
| FGSG_10855 | ADH1 / YOL086C | Alcohol dehydrogenase; fermentative isozyme active as homo- or heterotetramers; required for the reduction of acetaldehyde to ethanol, the last step in the glycolytic pathway; ADH1 has a paralog, ADH5, that arose from the whole genome duplication |
|  | ADH2 / YMR303C | Glucose-repressible alcohol dehydrogenase II; catalyzes the conversion of ethanol to acetaldehyde; involved in the production of certain carboxylate esters; regulated by ADR1 |
|  | ADH3 / YMR083W | Mitochondrial alcohol dehydrogenase isozyme III; involved in the shuttling of mitochondrial NADH to the cytosol under anaerobic conditions and ethanol production |
|  | ADH6 / YMR318C | NADPH-dependent medium chain alcohol dehydrogenase; has broad substrate specificity; member of the cinnamyl family of alcohol dehydrogenases; may be involved in fusel alcohol synthesis or in aldehyde tolerance; protein abundance increases in response to DNA replication stress |
|  | ADH7 / YCR105W | NADPH-dependent medium chain alcohol dehydrogenase; has broad substrate specificity; member of the cinnamyl family of alcohol dehydrogenases; may be involved in fusel alcohol synthesis or in aldehyde tolerance |
| FGSG_01322 | SMT3 YDR510W | Ubiquitin-like protein of the SUMO family; conjugated to lysine residues of target proteins; associates with transcriptionally active genes; regulates chromatid cohesion, chromosome segregation, APC-mediated proteolysis, DNA replication and septin ring dynamics; phosphorylated at Ser2 |
| FGSG_05930 | * |  |
| FGSG_10846 | COX11 / YPL132W | Protein required for delivery of copper to Cox1p; mitochondrial inner membrane protein; association with mitochondrial ribosomes suggests that copper delivery may occur during translation of Cox1p |
| FGSG_06526 | * |  |
| FGSG_00631 | RPS8A / YBL072C | Protein component of the small (40S) ribosomal subunit; homologous to mammalian ribosomal protein S8, no bacterial homolog; RPS8A has a paralog, RPS8B, that arose from the whole genome duplication |
| FGSG_01317 | GRX3 / YDR098C | Glutathione-dependent oxidoreductase; hydroperoxide and superoxide-radical responsive; monothiol glutaredoxin subfamily member along with Grx4p and Grx5p; protects cells from oxidative damage; with Grx4p, binds to Aft1p in iron-replete conditions, promoting its dissociation from promoters; evidence exists indicating that the translation start site is not Met1 as currently annotated, but rather Met36; GRX3 has a paralog, GRX4, that arose from the whole genome duplication |
|  | GRX4 / YER174C | Glutathione-dependent oxidoreductase; hydroperoxide and superoxide-radical responsive; monothiol glutaredoxin subfamily member along with Grx3p and Grx5p; protects cells from oxidative damage; with Grx3p, binds to Aft1p in iron-replete conditions, promoting its dissociation from promoters; mutant has increased aneuploidy tolerance; transcription regulated by Yap5p; GRX4 has a paralog, GRX3, that arose from the whole genome duplication |
| FGSG_04289 | HHF1 / YBR009C | Histone H4; core histone protein required for chromatin assembly and chromosome function; one of two identical histone proteins (see also HHF2); contributes to telomeric silencing; N-terminal domain involved in maintaining genomic integrity |
|  | HHF2 / YNL030W | Histone H4; core histone protein required for chromatin assembly and chromosome function; one of two identical histone proteins (see also HHF1); contributes to telomeric silencing; N-terminal domain involved in maintaining genomic integrity |
| FGSG_09618 | * |  |
| FGSG_11058 | * |  |
| FGSG_00802 | RPL23A / YBL087C | Ribosomal 60S subunit protein L23A; homologous to mammalian ribosomal protein L23 and bacterial L14; RPL23A has a paralog, RPL23B, that arose from the whole genome duplication |
|  | RPL23B / YER117W | Ribosomal 60S subunit protein L23B; homologous to mammalian ribosomal protein L23 and bacterial L14; RPL23B has a paralog, RPL23A, that arose from the whole genome duplication |
|  | MRPL38 / YKL170W | Mitochondrial ribosomal protein of the large subunit; appears as two protein spots (YmL34 and YmL38) on two-dimensional SDS gels; protein abundance increases in response to DNA replication stress |
| FGSG_03028 | * |  |
| FGSG_10212 | * |  |
| FGSG_07347 | ELO2 / YCR034W | Fatty acid elongase, involved in sphingolipid biosynthesis; acts on fatty acids of up to 24 carbons in length; mutations have regulatory effects on 1,3-beta-glucan synthase, vacuolar ATPase, and the secretory pathway; ELO2 has a paralog, ELO1, that arose from the whole genome duplication; lethality of the elo2 elo3 double null mutation is functionally complemented by human ELOVL1 and weakly complemented by human ELOVL3 or ELOV7 |
| FGSG_11064 | * |  |
| FGSG_00440 | * |  |
| FGSG_06938 | * |  |
| FGSG_00328 | MRPL16 / YBL038W | Mitochondrial ribosomal protein of the large subunit; homologous to bacterial L16 ribosomal protein; synthetic lethality with hac1 mutation suggests a possible role in synthesis of precursors for protein glycosylation |
| FGSG_00335 | * |  |
| FGSG_12955 | ATP16 / YDL004W | Delta subunit of the central stalk of mitochondrial F1F0 ATP synthase; F1F0 ATP synthase is a large, evolutionarily conserved enzyme complex required for ATP synthesis; F1 translationally regulates ATP6 and ATP8 expression to achieve a balanced output of ATP synthase genes encoded in nucleus and mitochondria; phosphorylated |
| FGSG_09906 | * |  |
| FGSG_06779 | * |  |
| FGSG_00769 | * |  |
| FGSG_00945 | CWC27 / YPL064C | Component of a complex containing Cef1p; putatively involved in pre-mRNA splicing; has similarity to S. pombe Cwf27p; protein abundance increases in response to DNA replication stress |
| FGSG_00362 | KIC1 / YHR102W | Protein kinase of the PAK/Ste20 family, required for cell integrity; physically interacts with Cdc31p (centrin), which is a component of the spindle pole body; part of the RAM network that regulates cellular polarity and morphogenesis |
| FGSG_02588 | RPS19A / YOL121C | Protein component of the small (40S) ribosomal subunit; required for assembly and maturation of pre-40 S particles; homologous to mammalian ribosomal protein S19, no bacterial homolog; mutations in human RPS19 are associated with Diamond Blackfan anemia; RPS19A has a paralog, RPS19B, that arose from the whole genome duplication |
|  | RPS19B / YNL302C | Protein component of the small (40S) ribosomal subunit; required for assembly and maturation of pre-40 S particles; homologous to mammalian ribosomal protein S19, no bacterial homolog; mutations in human RPS19 are associated with Diamond Blackfan anemia; RPS19B has a paralog, RPS19A, that arose from the whole genome duplication |
| FGSG_02507 | SIZ1 / YDR409W | SUMO E3 ligase; promotes attachment of small ubiquitin-related modifier sumo (Smt3p) to primarily cytoplasmic proteins; regulates Rsp5p ubiquitin ligase activity and is in turn itself regulated by Rsp5p; required for sumoylation of septins and histone H3 variant Cse4p, a prerequisite for STUbL-mediated Ub-dependent degradation; localizes to the septin ring; acts as an adapter between E2, Ubc9p and substrates; tends to compensate for survival of DNA damage in absence of Nfi1p |
| FGSG_01158 | * |  |
| FGSG_10772 | SED1 / YDR077W | Major stress-induced structural GPI-cell wall glycoprotein; associates with translating ribosomes, possible role in mitochondrial genome maintenance; ORF contains two distinct variable minisatellites; SED1 has a paralog, SPI1, that arose from the whole genome duplication |
| FGSG_06313 | CCT6 / YDR188W | Subunit of the cytosolic chaperonin Cct ring complex; related to Tcp1p, essential protein that is required for the assembly of actin and tubulins in vivo; contains an ATP-binding motif |
| FGSG_04072 | TMA20 / YER007C-A | Acetohydroxyacid reductoisomerase and mtDNA binding protein; involved in branched-chain amino acid biosynthesis and maintenance of wild-type mitochondrial DNA; found in mitochondrial nucleoids |
| FGSG_13190 | * |  |
| FGSG_10118 | ILV5 / YLR355C | Acetohydroxyacid reductoisomerase and mtDNA binding protein; involved in branched-chain amino acid biosynthesis and maintenance of wild-type mitochondrial DNA; found in mitochondrial nucleoids |
| FGSG_02077 | * |  |
| FGSG_12009 | * |  |
| FGSG_06692 | DDR48 / YMR173W | DNA damage-responsive protein; expression is increased in response to heat-shock stress or treatments that produce DNA lesions; contains multiple repeats of the amino acid sequence NNNDSYGS; protein abundance increases in response to DNA replication stress |
| FGSG_08560 | EGD2 / YHR193C | Alpha subunit of the nascent polypeptide-associated complex (NAC); involved in protein sorting and translocation; associated with cytoplasmic ribosomes |
| FGSG_06010 | * |  |
| FGSG_10246 | RPL10 / YLR075W | Ribosomal 60S subunit protein L10; homologous to mammalian ribosomal protein L10 and bacterial L16; responsible for joining the 40S and 60S subunits; regulates translation initiation; similar to members of the QM gene family; protein abundance increases under DNA replication stress; mutations in human homolog implicated in T-cell acute lymphoblastic leukemia and also autism spectrum disorders (ASD); human RPL10 can complement yeast null mutant |
| FGSG_13689 | * |  |
| FGSG_03969 | * |  |
| FGSG_07726 | * |  |
| FGSG_00514 | * |  |
| FGSG_10805 | UBC4 / YBR082C | Ubiquitin-conjugating enzyme (E2); key E2 partner with Ubc1p for the anaphase-promoting complex (APC); mediates degradation of abnormal or excess proteins, including calmodulin and histone H3; regulates levels of DNA Polymerase-{alpha} to promote efficient and accurate DNA replication; interacts with many SCF ubiquitin protein ligases; component of the cellular stress response; UBC4 has a paralog, UBC5, that arose from the whole genome duplication |
|  | UBC5 / YDR059C | Ubiquitin-conjugating enzyme; mediates selective degradation of short-lived, abnormal, or excess proteins, including histone H3; central component of the cellular stress response; expression is heat inducible; protein abundance increases in response to DNA replication stress; UBC5 has a paralog, UBC4, that arose from the whole genome duplication |
| FGSG_07480 | RPS1A / YLR441C | Ribosomal protein 10 (rp10) of the small (40S) subunit; homologous to mammalian ribosomal protein S3A, no bacterial homolog; RPS1A has a paralog, RPS1B, that arose from the whole genome duplication |
|  | RPS1B / YML063W | Ribosomal protein 10 (rp10) of the small (40S) subunit; homologous to mammalian ribosomal protein S3A, no bacterial homolog; RPS1B has a paralog, RPS1A, that arose from the whole genome duplication |
